# Control of NFAT Isoform Activation and NFAT-Dependent Gene Expression through Two Coincident and Spatially Segregated Intracellular Ca^2+^ Signals

**DOI:** 10.1016/j.molcel.2016.11.011

**Published:** 2016-11-17

**Authors:** Pulak Kar, Gary R. Mirams, Helen C. Christian, Anant B. Parekh

**Affiliations:** 1 Department of Physiology, Anatomy and Genetics, University of Oxford, Parks Road, Oxford, OX1 3PT, UK; 2 Computational Biology, Department of Computer Science, University of Oxford, Parks Road, Oxford, OX1 3QD, UK

## Abstract

Excitation-transcription coupling, linking stimulation at the cell surface to changes in nuclear gene expression, is conserved throughout eukaryotes. How closely related coexpressed transcription factors are differentially activated remains unclear. Here, we show that two Ca^2+^-dependent transcription factor isoforms, NFAT1 and NFAT4, require distinct sub-cellular InsP_3_ and Ca^2+^ signals for physiologically sustained activation. NFAT1 is stimulated by sub-plasmalemmal Ca^2+^ microdomains, whereas NFAT4 additionally requires Ca^2+^ mobilization from the inner nuclear envelope by nuclear InsP_3_ receptors. NFAT1 is rephosphorylated (deactivated) more slowly than NFAT4 in both cytoplasm and nucleus, enabling a more prolonged activation phase. Oscillations in cytoplasmic Ca^2+^, long considered the physiological form of Ca^2+^ signaling, play no role in activating either NFAT protein. Instead, effective sustained physiological activation of NFAT4 is tightly linked to oscillations in nuclear Ca^2+^. Our results show how gene expression can be controlled by coincident yet geographically distinct Ca^2+^ signals, generated by a freely diffusible InsP_3_ message.

## Highlights

• NFAT1 is activated by local Ca^2+^ entry, whereas NFAT4 also needs nuclear Ca^2+^
• Nuclear Ca^2+^ increases via InsP_3_ receptors located on the inner nuclear membrane
• NFAT1 and NFAT4 show very different rephosphorylation (deactivation) kinetics
• Slow deactivation of NFAT1 affords a form of short-term memory to gene expression

## Graphical Abstract

**Figure:**
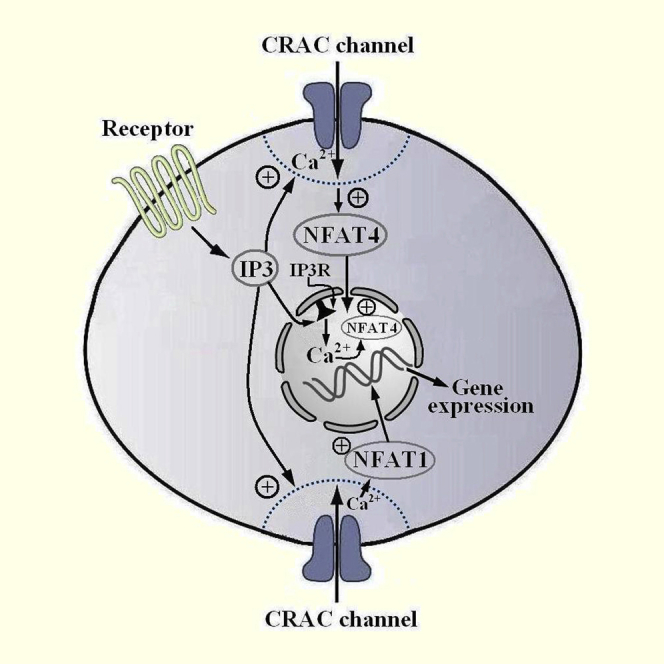

## Teaser

NFAT1 and NFAT4 are widely coexpressed transcription factors. NFAT1 is activated by Ca^2+^ microdomains near CRAC channels, whereas NFAT4 additionally requires nuclear Ca^2+^. Kar et al. now show that nuclear Ca^2+^ is raised by InsP_3_ receptors on the inner nuclear membrane. The dependence of NFAT isoforms on distinct sub-cellular Ca^2+^ signals regulates gene expression in cell- and stimulus-specific contexts.

## Introduction

Proteins of the NFAT family of transcription factors regulate expression of a multitude of genes that are essential for vertebrate development and function (Müller and Rao, 2010). The family consists of five members, of which four (NFAT1–NFAT4) are activated by intracellular Ca^2+^ signals. In resting cells, NFAT proteins are extensively phosphorylated and thereby trapped within the cytoplasm. Upon stimulation of cell-surface receptors that increase cytoplasmic Ca^2+^, the proteins are dephosphorylated by Ca^2+^-calmodulin-activated calcineurin, the target for immunosuppressants cyclosporine A and tacrolimus. Dephosphorylation of cytoplasmic NFAT exposes a lysine-rich nuclear localization sequence, enabling the transcription factor to migrate into the nucleus, in combination with the protein importin β (Rao et al., 1997). Nuclear export occurs after rephosphorylation by nuclear resident protein kinases, which unmasks a nuclear export sequence (Gwack et al., 2006), enabling CRM1 and Ran-GTP nuclear shuttle proteins to transport NFAT across the nuclear envelope via the nuclear pore complex (Kehlenbach et al., 1998).

Once within the nucleus, NFATs regulate gene transcription, both alone and in combination with other transcription factors such as the AP-1 complex and FOXP3 (Bettelli et al., 2005, Macián et al., 2001). Whether NFAT proteins cooperate with AP-1 in particular has important functional consequences. Studies with an engineered NFAT1 protein that was unable to interact with AP-1 led to anergy and exhaustion in CD4+ and CD8+ T cells (Macián et al., 2002, Martinez et al., 2015). By contrast, NFAT/AP-1 cooperation resulted in a productive immune response. In addition to this form of protein-protein crosstalk, we have recently found that NFAT1 and NFAT4, two homologs that are often coexpressed in cells and within the same spatial domain, are regulated by different patterns of cytoplasmic Ca^2+^ signal (Kar and Parekh, 2015). Ca^2+^ microdomains near open store-operated Ca^2+^ release-activated Ca^2+^ (CRAC) channels activate NFAT1 without the need for a rise in either bulk cytoplasmic or nuclear Ca^2+^ (Kar et al., 2011, Kar and Parekh, 2015). On the other hand, NFAT4 requires both CRAC channel-generated Ca^2+^ microdomains and a nuclear Ca^2+^ rise for sustained nuclear residency following stimulation with thapsigargin (Kar and Parekh, 2015), a widely used non-physiological agent that inhibits SERCA pumps and leads to store depletion and subsequent opening of CRAC channels. The nuclear Ca^2+^ increase that occurs upon stimulation with thapsigargin is a two-step process: Ca^2+^ first enters the cytoplasm through plasmalemmal CRAC channels and then diffuses into the nucleus, likely through nuclear pores. In this study, we asked what was the source of nuclear Ca^2+^ following stimulation with a physiological trigger. Unexpectedly, we find that the nuclear Ca^2+^ needed to activate NFAT4 is mobilized from the nuclear membrane itself. Our results reveal that two geographically distinct Ca^2+^ signals, one at the cell periphery and the other constrained within the nucleoplasm, are necessary and sufficient to drive NFAT4 activation and gene expression independent of the interspersed cytoplasmic Ca^2+^ signal.

## Results

### Physiological Stimulation Activates NFAT4 Independent of Bulk Cytoplasmic Ca^2+^

**Figure 1:**
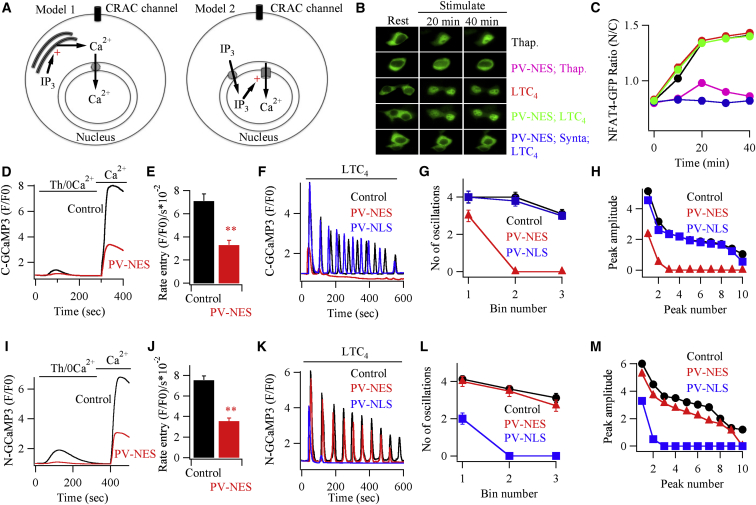
Buffering Cytoplasmic Ca^2+^ Does Not Impair Agonist-Evoked NFAT4 Nuclear Migration (A) Cartoons summarize the two possible models for increasing nuclear Ca^2+^ following cysLT1 receptor activation (see text for details). (B) Images show NFAT4-GFP nuclear migration for the different conditions indicated. (C) Graphs summarize aggregate data from between 7 and 9 cells for each condition. Colors of graphs correspond to labels in (B). Time denotes time after stimulation. (D) Cytoplasmic Ca^2+^ signals, measured with C-GCaMP3, following thapsigargin stimulation are shown for a control cell and one expressing PV-NES. (E) Histogram compares rate of rise of the cytoplasmic Ca^2+^ signal following Ca^2+^ readdition to thapsigargin-treated cells, as in (D). Bars are averages of 19 (control) and 11 (PV-NES) cells. (F) Cytoplasmic Ca^2+^ oscillations to 160 nM LTC_4_ are shown for the conditions indicated. Untagged PV constructs were used here. (G) Number of oscillations per 200 s bin after stimulation are compared. Each point is the mean of 10–21 cells. (H) Peak amplitude of each oscillation is compared between the different conditions. Peak amplitude represents (F/F0) − 1. (I–M) As in (D)–(H), but now nuclear Ca^2+^ was measured instead, using N-GCaMP3. In (J), control is mean from 22 cells and PV-NES from 19 cells. In (L), each point is the mean of between 11 and 26 cells. For most graphs, error bars are contained within the symbols. For the graphs and histograms, data are represented as mean ± SEM. See also Figures S1 and S2.

Activation of cell-surface G protein-coupled cysteinyl leukotriene type I (cysLT1) receptors with the pro-inflammatory molecule leukotriene C_4_ (LTC_4_) evokes repetitive baseline cytoplasmic Ca^2+^ oscillations that are similar to those widely observed in other cells types and in response to different phospholipase C-coupled agonists (Thomas et al., 1996). In principal, these receptors can raise nuclear Ca^2+^ and thus maintain activation of NFAT4 in one of two ways: by an increase in bulk cytoplasmic Ca^2+^, arising mainly from Ca^2+^ release through inositol 1,4,5-trisphosphate (InsP_3_) receptors embedded in the endoplasmic reticulum (with a contribution from Ca^2+^ entry through CRAC channels), which then invades the nucleus (Model 1; Figure 1A), or by Ca^2+^ release directly into the nucleoplasm from nuclear InsP_3_ receptor channels (Model 2; Figure 1A).

Model 1 predicts that an increase in cytoplasmic Ca^2+^ buffering should prevent NFAT4 activation by cysLT1 receptor stimulation because this manipulation would reduce the rate and extent of Ca^2+^ diffusion from the cytoplasm into the nucleus. By contrast, the expectation from Model 2 is that NFAT4 activation will be unaffected by changes in cytoplasmic Ca^2+^ buffering capacity. To test these predictions, we overexpressed the cytoplasmic Ca^2+^ buffer parvalbumin that had been genetically manipulated to express only in the cytoplasm through addition of a nuclear export sequence (PV-NES) (Kar and Parekh, 2015). Although stimulation with thapsigargin led to robust migration of NFAT4-GFP from the cytoplasm into the nucleus in control cells, expression of PV-NES largely suppressed NFAT4 migration (Figures 1B and 1C) (Kar and Parekh, 2015). PV-NES therefore buffers cytoplasmic Ca^2+^ sufficiently well to prevent a rise in bulk cytoplasmic Ca^2+^ following CRAC channel opening from invading the nucleus to maintain active NFAT4. Activation of cysLT1 receptors with LTC_4_ also resulted in robust nuclear accumulation of NFAT4 (Figures 1B and 1C). However, this was unaffected by the presence of PV-NES (Figures 1B and 1C). Ca^2+^ microdomains near open CRAC channels remained essential for NFAT4 activation by LTC_4_ in the presence of PV-NES because channel block with Synta66 prevented nuclear NFAT4 migration (Figures 1B and 1C).

To confirm that PV-NES indeed reduced stimulus-evoked Ca^2+^ signals, we measured bulk cytoplasmic Ca^2+^ with the fluorescent Ca^2+^ indicator dye Fura-2. The Ca^2+^ response was significantly decreased by the presence of PV-NES following stimulation with thapsigargin (Figure S1). The intracellular Ca^2+^ signal measured with Fura-2 includes contributions from the cytoplasm and the nucleus. To measure Ca^2+^ in each compartment, we used the genetically encoded fluorescent indicator protein GCaMP3, engineered to express either a nuclear export sequence (GCaMP3-NES) or nuclear localization sequence (GCaMP3-NLS), to report Ca^2+^ in the cytoplasm or nucleus, respectively. In control cells expressing GCaMP3-NES, thapsigargin activated a robust Ca^2+^ signal due to Ca^2+^ influx, but the response was reduced by ∼60% following expression of PV-NES (Figures 1D and 1E). Similarly, cytoplasmic Ca^2+^ oscillations to LTC_4_ ran down more quickly (Figures 1F and 1G) and were smaller in size (Figures 1F and 1H) in the presence of PV-NES. The nuclear Ca^2+^ rise induced by thapsigargin (measured with GCaMP3-NLS) was also substantially reduced by PV-NES (Figures 1I and 1J), as expected since this increase is secondary to the cytoplasmic Ca^2+^ rise.

LTC_4_ evoked numerous nuclear Ca^2+^ oscillations in control cells, and these were largely unaffected by the presence of PV-NES (Figures 1K–1M), as was the case for NFAT4 migration (Figures 1B and 1C). When parvalbumin was targeted to the nucleus instead (PV-NLS), NFAT4 migration in response to LTC_4_ challenge was suppressed (Figure S2) (Kar and Parekh, 2015), as were the nuclear Ca^2+^ oscillations (Figures 1K–1M). The cytoplasmic Ca^2+^ oscillations evoked by LTC_4_ were unaffected by the presence of PV-NLS (Figures 1F–1H).

**Figure 2:**
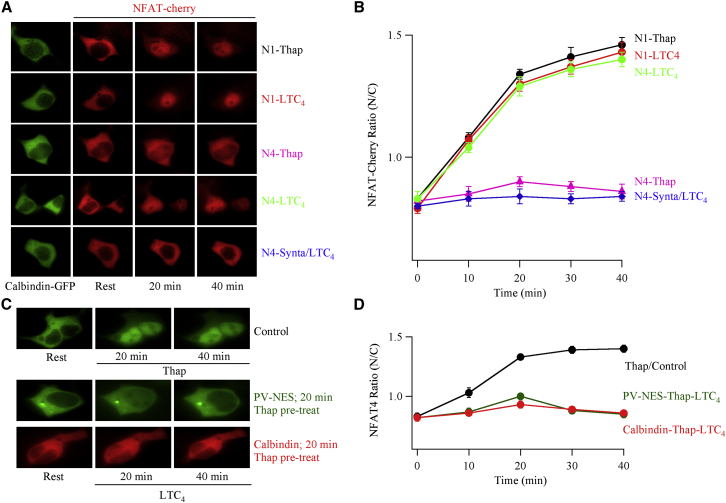
The Cytoplasmic Ca^2+^ Binding Protein Calbindin D_28K_ Prevents Nuclear Migration of NFAT4, but Not NFAT1 (A) Images compare NFAT1- or NFAT4-cherry movement in cells expressing calbindin D_28K_-GFP and were taken at different times after stimulation, as indicated. NFAT constructs used are shown on the right of each row of images, where N1 and N4 denote NFAT1 and NFAT4. Stimuli are also shown on the right. (B) Aggregate data from experiments as in (A) are summarized. Each point is the mean of between 8 and 13 individual cells. The N1-Thap curve has been offset upward by 0.03, to increase clarity. (C) In cells expressing PV-NES (untagged) or calbindin D_28K_-GFP (indicated on the right of each image), stimulation with LTC_4_ after thapsigargin (1 μM) failed to show any NFAT4 movement. LTC_4_ was applied 20 min after pre-treatment with thapsigargin. Upper images show rest and then 20 and 40 min after thapsigargin stimulation (control recording from the same cell preparation). In the control and PV-NES experiments, NFAT4-GFP was expressed. In coexpression with calbindin D_28K_-GFP, NFAT4-cherry was used instead. (D) Aggregate data are compared for the conditions shown. Each point is the mean of between 9 and 15 cells. For the graphs, data are represented as mean ± SEM.

Equivalent results were obtained when we overexpressed a different cytoplasmic Ca^2+^-binding protein, calbindin D_28K_-GFP: NFAT4-cherry failed to accumulate in the nucleus after stimulation with thapsigargin (middle set of images in Figure 2A; Figure 2B). By contrast, activation of cysLT1 receptors triggered robust migration of NFAT4-cherry into the nucleus in cells expressing calbindin D_28K_-GFP, which was prevented by Synta66 (Figures 2A and 2B).

NFAT1 activation, which is driven only by local Ca^2+^ entry through CRAC channels, was unaffected by the presence of PV-NES (Kar and Parekh, 2015) or calbindin D_28K_-GFP (upper set of images in Figure 2A; Figure 2B), regardless of whether the stimulus was thapsigargin or LTC_4_.

Collectively, these data show that cysLT1 receptor stimulation activates NFAT4 even when a rise in bulk cytoplasmic Ca^2+^ has been impaired. The findings are therefore incompatible with the various scenarios embodied in Model 1 (Figure 1), where nuclear Ca^2+^ is totally contingent upon a cytoplasmic Ca^2+^ rise. Instead, our data are consistent with Model 2, where the nuclear Ca^2+^ increase required for maintained NFAT4 activation is independent of cytoplasmic Ca^2+^. Model 2 posits that nuclear Ca^2+^ is increased by activation of nuclear InsP_3_ receptors and is therefore causally attendant to a rise in nucleoplasmic InsP_3_. The nuclear envelope is a functional Ca^2+^ store due to the presence of SERCA pumps on the outer membrane and InsP_3_ receptors on both the inner and outer membranes (Alonso and García-Sancho, 2011, Gerasimenko et al., 1996), with InsP_3_ releasing stored Ca^2+^ directly into the nucleoplasm (Echevarría et al., 2003, Humbert et al., 1996, Zima et al., 2007). Although LTC_4_ triggered NFAT4 accumulation in the nucleus in PV-NES- and calbindin-expressing cells, it failed to do so if cells were pre-exposed to thapsigargin (Figures 2C and 2D). This supports Model 2 in that sustained activation of NFAT4 by receptor stimulation requires a functional nuclear Ca^2+^ store.

### Buffering Nuclear InsP_3_ Prevents the Nuclear Ca^2+^ Rise to Agonist and Subsequent Sustained NFAT4 Activation

**Figure 3:**
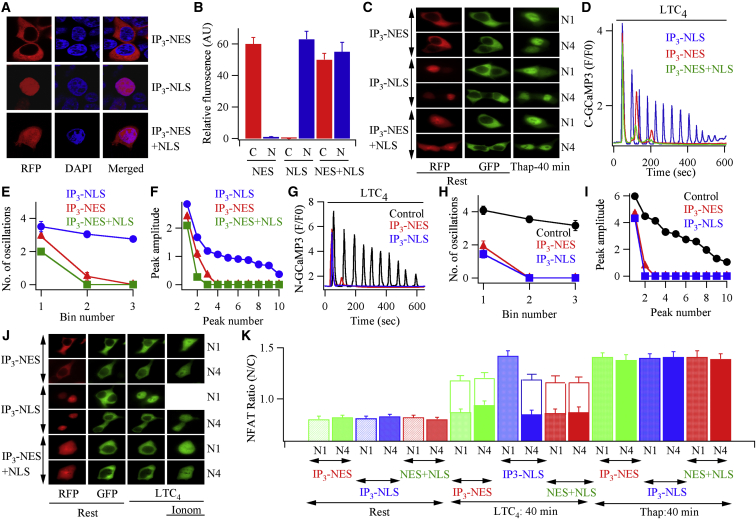
Buffering Nuclear InsP_3_ Prevents Nuclear Accumulation of NFAT4 (A) Confocal images compare sub-cellular distribution of RFP-tagged InsP_3_ buffer containing either a nuclear export sequence (IP_3_-NES; upper panel), InsP_3_ buffer containing a nuclear localization sequence (IP_3_-NLS, middle panel), or both (lower panel). DAPI was used to stain the nucleus. (B) Fluorescence intensities of the InsP_3_ buffers are compared between the cytoplasmic and nuclear compartments, denoted C and N, respectively. Each bar is the mean of between 9 and 15 cells. (C) Migration of NFAT1-GFP (denoted N1) and NFAT4-GFP (N4) into the nucleus is compared following stimulation with thapsigargin for 40 min in the presence of the different IP_3_ buffers (RFP-tagged), indicated on the left. (D) Cytoplasmic Ca^2+^ oscillations are compared between cells expressing RFP-tagged IP_3_-NLS, IP_3_-NES, or both. (E) The number of oscillations to LTC_4_ produced each 200 s bin are compared for the different conditions. Each point is the mean of between 8 and 11 cells. (F) The amplitude of each Ca^2+^ oscillation is compared for the conditions shown. Data for (E) and (F) were extracted from experiments as in (D). (G) Nuclear Ca^2+^ oscillations are compared for the different conditions indicated. (H) The number of nuclear Ca^2+^ oscillations per 200 s bin is shown for each condition. Each point is the mean of between 10 and 26 cells. (I) The peak amplitude of each nuclear Ca^2+^ oscillation is compared. Data for (H) and (I) were extracted from experiments as in (G). In (G)–(I), control denotes mock-transfected cells. (J) Images compare movement of NFAT1-GFP or NFAT4-GFP for the conditions shown. IP_3_ buffers were tagged with RFP. LTC_4_ was applied at 160 nM for 40 min and ionomycin (ionom) was 2 μM. (K) Histogram summarizes data for the various conditions shown. Rest denotes the unstimulated state. N1 and N4 indicate NFAT1-GFP and NFAT4-GFP. Open bars above filled histograms denote the rescue of NFAT movement following stimulation with ionomycin for 20 min. Each bar is the mean from three independent experiments. In (B), (E), (F), (H), (I), and (K), data are represented as mean ± SEM. See also Figure S3.

We designed experiments to test Model 2 more directly. A key prediction is that buffering nuclear InsP_3_ should impair InsP_3_ from activating InsP_3_ receptors in the inner nuclear membrane, preventing the rise in nuclear Ca^2+^ and thus sustained nuclear NFAT4 activation. To address this, we dampened InsP_3_ increases in either the cytoplasm or the nucleus by exploiting genetically encoded InsP_3_ buffers. We expressed a construct containing the InsP_3_ binding site of the InsP_3_ receptor tagged with RFP and engineered to express either a cytoplasmic or nuclear localization sequence, which we refer to as IP_3_ buffer-NES-RFP and IP_3_ buffer-NLS-RFP, respectively. Expression of IP_3_ buffer-NES-RFP was localized to the cytoplasm (Figures 3A and 3B), whereas IP_3_ buffer-NLS-RFP was restricted to the nucleus (Figures 3A and 3B). Coexpression of both buffers resulted in a similar distribution between nucleus and cytoplasm. Stimulation with thapsigargin, which evokes Ca^2+^ signals independent of InsP_3_, resulted in robust nuclear accumulation of NFAT1-GFP or NFAT4-GFP despite the presence of IP_3_ buffer-NES-RFP, IP_3_ buffer-NLS-RFP, or both (Figure 3C; aggregate data are summarized in Figure 3K). Therefore these buffers per se do not interfere with NFAT migration.

**Figure 4:**
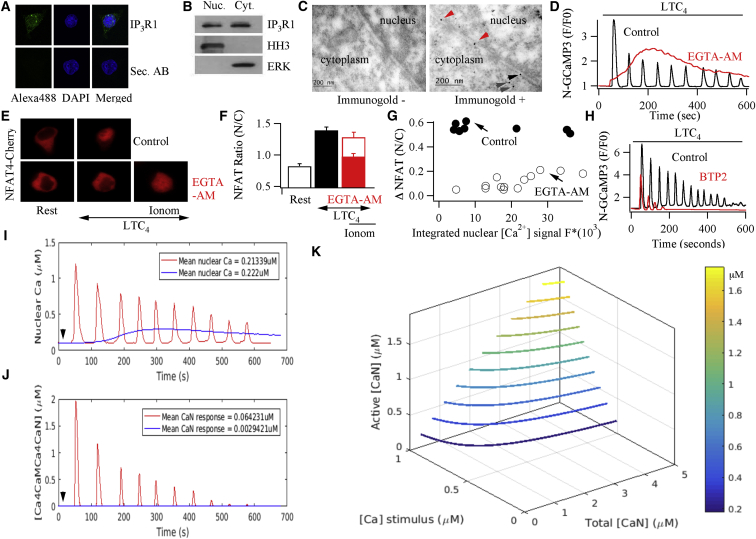
Nuclear Ca^2+^ Oscillations Are Generated by Nuclear InsP_3_ Receptors and Are Effective in Maintaining NFAT4 Activity (A) Immunostaining reveals the presence of type I InsP_3_ receptors in the cytoplasm and nucleus. No staining is seen when secondary antibody (Sec. AB) alone is used. Secondary antibody was conjugated with Alexa 488 for visualization. (B) Western blots of cytoplasmic and nuclear extract show the presence of type I InsP_3_ receptors in both compartments. Similar results were obtained in two independent experiments. (C) Immunogold labeling reveals the presence of type I InsP_3_ receptors in the inner nuclear membrane. Left-hand panel denotes a control micrograph and the right-hand one after immunogold labeling of type I InsP_3_ receptors. Red arrows denote InsP_3_R on the inner nuclear membrane, black arrow shows receptor on the outer nuclear membrane, and gray ones denote receptor in the endoplasmic reticulum. (D) Comparison of nuclear Ca^2+^ signals to LTC_4_ in a control cell and in one in which the cytoplasm and nucleoplasm had been loaded with EGTA via the form EGTA-AM. (E) Loading the cell with EGTA reduces NFAT4-cherry nuclear migration in response to LTC_4_. Migration can be rescued by stimulating cells with ionomycin (2 μM; 20 min). (F) Aggregate data from several experiments as in (E) are summarized. Open bar above EGTA-AM denotes the extent of rescue by ionomycin. Data are represented as mean ± SEM. (G) Graph plots NFAT4-cherry nuclear accumulation against the integrated nuclear Ca^2+^ signal in control cells and cells loaded with EGTA. Each point depicts NFAT4 movement and integrated nuclear Ca^2+^ from the same cell. The y axis represents peak NFAT nuclear/cytoplasmic ratio after stimulation with LTC_4_ minus the NFAT nuclear/cytoplasmic ratio before stimulation. (H) Nuclear Ca^2+^ oscillations evoked by LTC_4_ run down quickly when Ca^2+^ entry through CRAC channels is blocked with 10 μM BTP2. (I) Estimated nuclear Ca^2+^ concentration for an oscillatory response in a control cell is compared with a plateau-type response in an EGTA-loaded cell, following stimulation with 160 nM LTC_4_. To calibrate the nuclear Ca^2+^ signal in intact cells, following expression of NGCaMP3, Rmin and Rmax were obtained by stimulating cells with 5 μM ionomycin either in Ca^2+^-free external solution (with fluorescence measured ∼15 min later) or 10 mM Ca^2+^ external solution, respectively. The K_D_ for nuclear GCaMP3 was taken as 660 nM from the literature. The estimated Ca^2+^ concentrations are, at best, only an approximation (see text). Arrow denotes LTC_4_ application. (J) The graph plots the predicted build-up of nuclear Ca^2+^-calmodulin-calcineurin following stimulation with LTC_4_ in a control cell (red trace) and in one loaded with EGTA (blue trace). (K) Contour plot depicts active nuclear calcineurin as a function of nuclear Ca^2+^ concentration and total nuclear calcineurin available. See also Figure S4, Table S1, and description of the model in Supplemental information.

After expression of InsP_3_ buffer-NES-RFP, cytoplasmic (Figures 3D–3F) and nuclear (Figures 3G–3I) Ca^2+^ signals to cysLT1 receptor activation were significantly reduced. Activation of either NFAT1 or NFAT4 was also suppressed by InsP_3_ buffer-NES-RFP (Figures 3J and 3K), as expected since cytoplasmic InsP_3_ is required to deplete the endoplasmic reticulum Ca^2+^ store for CRAC channel activation following cysLT1 receptor stimulation. Movement could be rescued by a dose of ionomycin that raises bulk cytoplasmic Ca^2+^ to high levels independent of store-operated Ca^2+^ influx (Figures 3J and 3K; Kar and Parekh 2015). Markedly different results were obtained when InsP_3_ buffer-NLS-RFP was expressed instead. Cytoplasmic Ca^2+^ oscillations to LTC_4_ were now largely unaffected (Figures 3D–3F), whereas the nuclear Ca^2+^ signals were suppressed (Figures 3G–3I). Despite cytoplasmic Ca^2+^ signals being normal in the presence of IP_3_ buffer-NLS, activation of cysLT1 receptors now failed to stimulate NFAT4 accumulation in the nucleus (Figures 3J and 3K). Movement could subsequently be rescued by ionomycin. Simultaneous measurements of cytoplasmic and nuclear Ca^2+^ in the same cells confirmed that Ca^2+^ oscillated in both compartments after stimulation with LTC_4_, but only the nuclear Ca^2+^ oscillations were abolished by InsP_3_ buffer-NLS (Figure S3). Oscillations in cytoplasmic and nuclear Ca^2+^ in both compartments were suppressed by coexpression of InsP_3_ buffer-NES and InsP_3_ buffer-NLS (Figure S3). Migration of NFAT1 into the nucleus in response to LTC_4_ was unaffected by InsP_3_ buffer-NLS-RFP (Figures 3J and 3K), consistent with it requiring only local Ca^2+^ entry through CRAC channels for activation. Nuclear migration of either NFAT1 or NFAT4 was suppressed by coexpression of both InsP_3_ buffers (Figures 3J and 3K). In aggregate, these data suggest that the nuclear Ca^2+^ rise required for NFAT4 residency within the nucleus is accomplished through Ca^2+^ release by nuclear InsP_3_. In support of this, immunostaining revealed that type I InsP_3_ receptors localized to the nucleus as well as the cytoplasm (Figure 4A). InsP_3_ receptors were also detected in both nuclear and cytoplasmic extract (Figure 4B).

In order for InsP_3_ buffer-NLS to suppress nuclear Ca^2+^ oscillations, leaving cytoplasmic ones intact, InsP_3_ receptors must be embedded within the inner nuclear membrane facing the nucleoplasm. To assess this, we used electron microscopy with immunogold labeling of type I InsP_3_ receptors, an approach that provides the spatial resolution to resolve the specific nuclear location of proteins. Whereas no detectable immunogold labeling was seen in control cells not exposed to InsP_3_ receptor antibody (Figure 4C, left-hand panel, labeled immunogold−), the inner nuclear membrane revealed particles when InsP_3_ antibody was present (Figure 4C, right-hand panel, labeled immunogold+).

In some studies, the nuclear Ca^2+^ rise is secondary to the cytoplasmic Ca^2+^ increase, which rapidly propagates into the nucleus (Lin et al., 1994, Lipp et al., 1997). On the other hand, nuclear Ca^2+^ can increase independently of cytoplasmic Ca^2+^ (Echevarría et al., 2003, Humbert et al., 1996, Zima et al., 2007). In our experiments with cysLT1 receptor activation, cytoplasmic Ca^2+^ oscillations did not invade the nucleoplasm to the extent needed to maintain NFAT4 residency because InsP_3_ buffer-NLS-RFP greatly reduced the nuclear Ca^2+^ signals (Figures 3G–3I) and suppressed transcription factor accumulation (Figure 3K), despite cytoplasmic Ca^2+^ signaling remaining intact (Figures 3D–3F; Figure S3). Furthermore, buffering cytoplasmic Ca^2+^ with parvalbumin-NES abolished cytoplasmic calcium oscillations to LTC_4_ (Figures 1F–1H), yet nuclear Ca^2+^ signaling (Figures 1K–1M) and NFAT4 activation (Figure 1C) were unaffected. We also considered the possibility that InsP_3_ released Ca^2+^ into the cytoplasm from receptors located in the outer nuclear membrane and Ca^2+^ then diffused into the nucleus through nuclear pores. Because PV-NES had no inhibitory effect on nuclear Ca^2+^ signals evoked by agonist, this possibility is unlikely.

How does cysLT1 receptor activation increase nuclear Ca^2+^ independently of cytoplasmic Ca^2+^? Our finding that InsP_3_ buffer-NES-RFP suppressed the nuclear Ca^2+^ rise to LTC_4_ demonstrates that InsP_3_ is first produced in the cytoplasm and then diffuses into the nucleus, rather than local generation of InsP_3_ within the nucleus itself. The lifetime of InsP_3_ in the cytoplasm is sufficiently long for it to diffuse many times across a cell with dimensions similar to that of a HEK cell before it is broken down (Allbritton et al., 1992).

### For the Same Integrated Nuclear Ca^2+^ Signal, Nuclear Ca^2+^ Oscillations Are More Effective in Maintaining NFAT4 Activation than a Bulk Nuclear Ca^2+^ Rise

We asked whether nuclear Ca^2+^ oscillations conferred a signaling advantage to sustained NFAT4 activation compared with a non-oscillatory Ca^2+^ increase that raised nucleoplasmic Ca^2+^ to a similar extent over the same stimulation period (integrated Ca^2+^ signal). Simultaneous measurements of nuclear Ca^2+^ using GCaMP3-NLS and nuclear accumulation of NFAT4-cherry showed that oscillatory nuclear Ca^2+^ signals induced by LTC_4_ (Figure 4D) evoked robust nuclear accumulation of NFAT4 (Figures 4E and 4F) for only a small integrated nuclear Ca^2+^ signal (Figure 4G; labeled control). In cells loaded with the slow Ca^2+^ chelator EGTA, which increases the buffering capacity of the nucleoplasm (Figure S3 in Kar and Parekh, 2015), nuclear Ca^2+^ oscillations to LTC_4_ were replaced by a steady increase in nuclear Ca^2+^ (Figure 4D). Integration of these signals showed similar or greater total nuclear Ca^2+^ over the same time period compared with oscillatory responses (Figure 4G), yet NFAT4 accumulation was substantially less (Figures 4E–4G).

### The Nuclear Ca^2+^ Store Is Replenished by Store-Operated Ca^2+^ Entry

Ca^2+^ entry through CRAC channels is required to refill the endoplasmic reticulum with Ca^2+^. Since the endoplasmic reticulum is contiguous with the outer nuclear membrane, we asked whether Ca^2+^ entry through CRAC channels was likewise essential for repletion of the nuclear store. Whereas numerous nuclear Ca^2+^ oscillations were seen in control cells stimulated with LTC_4_, the Ca^2+^ signals ran down quickly in the presence of the CRAC channel blocker BTP2 (Figure 4H; Figure S4). Similar results were obtained when cells were stimulated either in the presence of another CRAC channel blocker (Synta66) or in the absence of external Ca^2+^ (data not shown).

Ca^2+^ influx contributes little to the size or decay rate of each cytoplasmic Ca^2+^ oscillation evoked by LTC_4_ (Di Capite et al., 2009). The finding that the nuclear store is replenished by Ca^2+^ flux through CRAC channels at the cell surface is therefore a remarkable example of Ca^2+^ tunneling (Mogami et al., 1997), where local Ca^2+^ entry can be transported to distant cytoplasmic regions via uptake into the endoplasmic reticulum.

### Mathematical Modeling of Nuclear Calcineurin Activation

We simulated active calcineurin build-up in the nucleus (Figures 4I–4K) for different patterns of nuclear Ca^2+^ signal, using a reduction of the Saucerman and Bers model (Saucerman and Bers, 2008), as suggested by Bazzazi et al. (Bazzazi et al., 2015; see Supplemental Information for further details). To model active calcineurin levels, an estimate of nuclear Ca^2+^ concentration was needed. We therefore attempted to calibrate the GCaMP3 signal, as described in the legend to Figure 4I. The concentrations shown in Figure 4I are, however, only a rough estimate because of the inherent difficulties in calibrating a fluorescent probe within the nucleoplasm of an intact cell (Bootman et al., 2009), coupled with GCaMP3 being a non-ratiometric probe. Notwithstanding these limitations, the model suggests calcineurin activity oscillates in the nucleus (Figure 4J) with a pattern similar to the nuclear Ca^2+^ oscillations (Figure 4I). By contrast, nuclear calcineurin activity is very low when a small-amplitude but long-lasting nuclear Ca^2+^ rise occurs. A contour plot comparing active calcineurin with Ca^2+^ concentration and total nuclear calcineurin (Figure 4K) revealed that the same level of active calcineurin could be achieved by having either high total nuclear calcineurin combined with low nuclear Ca^2+^ or low total nuclear calcineurin coupled with high nuclear Ca^2+^. The contour plot also shows that large nuclear Ca^2+^ spikes enable robust activation of a small number of nuclear calcineurin molecules, whereas more sustained smaller Ca^2+^ rises are much less effective. This suggests an advantage to large oscillatory Ca^2+^ signaling: in an individual cell having a fixed total number of effector molecules like calcineurin, a small number of effectors will be activated strongly by large-amplitude Ca^2+^ oscillations, compared with weak recruitment of a greater number of effectors in response to smaller-amplitude but more prolonged Ca^2+^ signals. For an effector that is an enzyme like calcineurin, a small fraction of active molecules should be sufficient to engender a strong downstream response.

### NFAT4 Is Rephosphorylated within the Nucleus Much Faster than NFAT1

**Figure 5:**
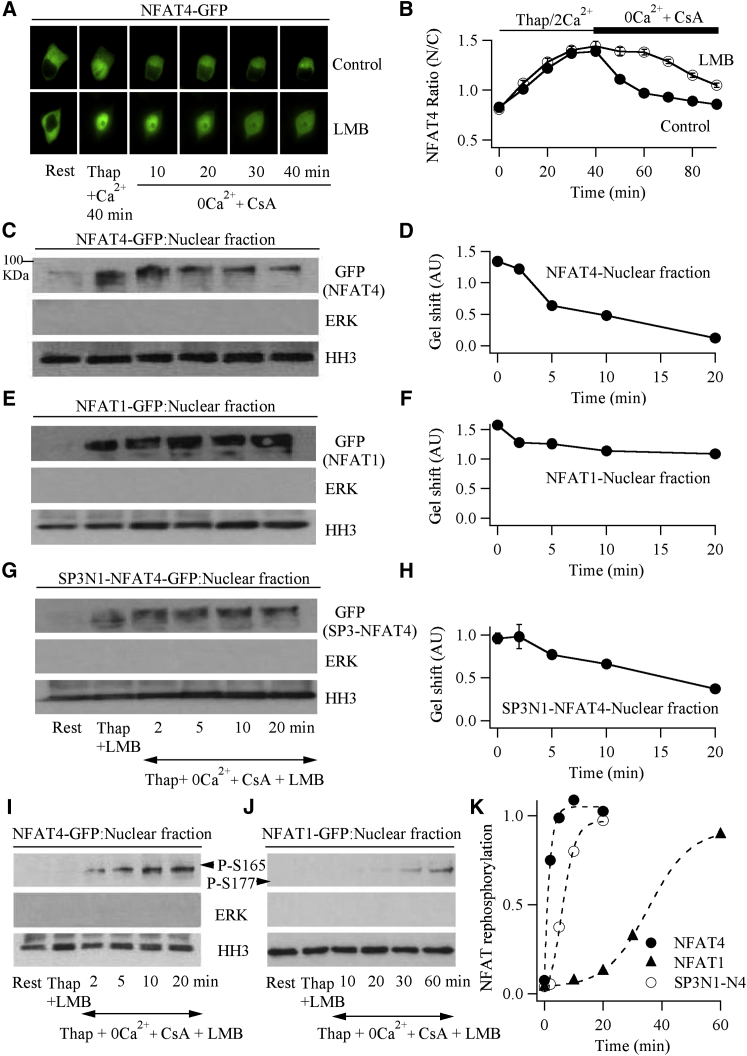
NFAT4 Is Rephosphorylated within the Nucleus Considerably More Quickly Than NFAT1 (A) Images compare nuclear export of NFAT4-GFP between a control cell and one exposed to leptomycin B (LMB; 100 nM). Nuclear export was initiated by removal of external Ca^2+^ in the presence of cyclosporine A. (B) Aggregate data from several experiments are compared. (C) Western blot shows gel shift of NFAT4-GFP from nuclear extract after stimulation and then at different times (2–20 min) after initiation of export (see bottom of G for timings). (D) Graph plots relative gel shift as a function of time up to 20 min after the initiation of nuclear export. A decreased gel shift denotes rephosphorylation. (E) Gel shift for nuclear NFAT1-GFP under conditions identical to (C). (F) Graph shows relative gel shift for NFAT1-GFP, as in (D). (G) Gel shift for the SP3NFAT1-NFAT4-GFP construct. (H) Graph plots relative gel shift for SP3NFAT1-NFAT4-GFP, as in (D). For the gels in (C), (E), and (G), conditions for each lane are shown only in (G). For the resting condition (indicated as Rest), no detectable NFAT was seen in any of the gels because the gels show nuclear extract and, at rest, there is very little NFAT in the nucleus. (I) Blot compares kinetics of phosphorylation of serine 165 in NFAT4 from the nuclear fraction, denoted as P-S165. (J) As in (I), but phosphorylation of serine 177 (P-S177) in NFAT1 was measured. (K) Graphs plot the kinetics of phosphorylation of serine 165 NFAT4 and SP3N1-N4 or serine 177 in NFAT1. In (C), (E), (G), (I), and (J), HH3 (histone H3) was used as a nuclear marker and ERK was taken as a cytoplasmic marker. Rest denotes the non-stimulated state. Thap + LMB represents the gel shift seen 40 min after stimulation with thapsigargin in the presence of LMB and external Ca^2+^, when NFAT had fully accumulated in the nucleus. Nuclear extract was isolated at the different times indicated. In all panels, 100 nM leptomycin B was added 10 min before thapsigargin and then maintained throughout. In the graphs, data are represented as mean ± SEM. See also Figure S5.

NFAT4 is exported out of the nucleus ∼8 times faster than NFAT1 (Kar and Parekh, 2015, Yissachar et al., 2013), an effect due partly to differences in the respective SP-3 motifs (Kar and Parekh, 2015). The nuclear export of each isoform is a two-step process involving rephosphorylation within the nucleoplasm followed by exit through the nuclear pores in a complex with shuttle proteins CRM1 and Ran-GTP. The relative contributions of these steps are unknown. We hypothesized that the main difference in NFAT nuclear export rates was due to differences in nuclear rephosphorylation rather than nuclear transport, and this was why nuclear Ca^2+^ and thus active calcineurin was so important for NFAT4 accumulation. To address this, we exposed cells to leptomycin B, an effective inhibitor of CRM1-dependent export (Fornerod et al., 1997). In the presence of leptomycin B, NFAT4 accumulated within the nucleus following CRAC channel activation at a similar rate to control cells (Figures 5A and 5B). Following nuclear accumulation, we triggered nuclear export by termination of Ca^2+^ influx combined with inhibition of calcineurin with cyclosporine A (Kar et al., 2011). Compared with control cells, where NFAT4 exited the nucleus with a time constant of ∼10 min (Kar and Parekh, 2015), nuclear export was suppressed for ∼20 min in leptomycin B-treated cells (Figures 5A and 5B). Thereafter, NFAT4 export proceeded gradually, reflecting either delayed leptomycin B-insensitive transport or loss of leptomycin B activity over time. Regardless, the 20 min hiatus in NFAT4 export in leptomycin B enabled us to measure the kinetics of NFAT4 rephosphorylation within the nucleus in the absence of export. Gel shifts from western blots of nuclear extract revealed that NFAT4 was rephosphorylated quickly (Figure 5C), with a half-time of ∼4.5 min (Figure 5D). By contrast, only modest nuclear phosphorylation of NFAT1 occurred within 20 min (Figures 5E and 5F). An NFAT4 construct in which the SP-3 region of NFAT4 had been replaced with that from NFAT1 was rephosphorylated more slowly than NFAT4 but faster than NFAT1 (Figures 5G and 5H; half-time of ∼15 min), supporting the view that the SP-3 region is an important determinant of nuclear NFAT rephosphorylation (Kar and Parekh, 2015). For the 20 min that NFAT-GFP export was suppressed (Figure 5B), no increase in cytoplasmic NFAT-GFP was detectable (Figures S5A–S5C). Hence, over this time frame, leptomycin B fully inhibits NFAT nuclear export.

In the Ar-5 T cell clone, significant rephosphorylation of NFAT1 occurs within 15 min (Loh et al., 1996), which is considerably faster than the rate we find in HEK293 cells (Figure 5F). In RBL-2H3 cells, NFAT1 was exported out of the nucleus with a rate constant of ∼0.06 min^−1^ (Yissachar et al., 2013), which is ∼4-fold faster than the rate constant (∼0.014 min^−1^) for the same isoform in HEK293 cells. Hence, the kinetics of NFAT1 export varies between different cell types, perhaps reflecting the activities of nuclear kinases.

Although gel-shift analysis is widely used to measure NFAT phosphorylation status, shifts can be induced by other post-translational modifications. Additionally, since multiple serines are phosphorylated and then dephosphorylated (Okamura et al., 2000), the shift is a smear rather than a discrete change, which renders quantification somewhat selective. Because we analyzed the shifts for NFAT1 and NFAT4 in the same way, the relative difference in dephosphorylation/rephosphorylation kinetics seems valid. Nevertheless, we utilized an alternative approach to compare the kinetics of NFAT4 rephosphorylation/dephosphorylation with that of NFAT1. Serine 165 on NFAT4 is close to serine 177 on NFAT1; both are phosphorylated at rest and dephosphorylated by calcineurin, and commercial antibodies to these phosphorylated residues are available. We therefore tracked the phosphorylated state of each NFAT isoform using phospho-specific antibodies. At rest, both isoforms were phosphorylated and restricted to the cytoplasmic fraction (Figures S5D and S5E), with no detectable phosphorylated form in the nucleus (Figures 5I and 5J). Stimulation with thapsigargin for 30 min in the presence of leptomycin B led to a marked decrease in phosphorylation of both NFAT isoforms in cytoplasmic extract (Figures S5D and S5E), with no detectable change in the phosphorylated nuclear levels (Figures 5I and 5J). This is because only dephosphorylated NFAT migrates into the nucleus, and we are tracking phosphorylated residues. However, subsequent removal of external Ca^2+^ coupled with addition of cyclosporine A in the continued presence of leptomycin B led to rapid rephosphorylation of nuclear NFAT4 (Figure 5I) with a half-time of ∼1.1 min (Figure 5K). Serine 177 on NFAT1, by contrast, was phosphorylated considerably more slowly (Figures 5J and 5K), with a half-time of ∼35 min. In the SP3N1-NFAT4 chimera, serine 165 was rephosphorylated more slowly than NFAT4 but faster than NFAT1 (Figure 5K and Figures S5F and S5G; half-time of ∼6.4 min), consistent with the gel-shift data in Figure 5H.

It is important to note that these phosphorylation studies detect only one phosphorylated residue in each NFAT isoform and do not represent the kinetics of total rephosphorylation. It is possible that serine 165 is the first residue in NFAT4 to be rephosphorylated, whereas serine 177 in NFAT1 is among the last, which could explain the different kinetics. The fact that serine 165 in the SP3N1-NFAT4 chimera was phosphorylated ∼6-fold more slowly than in NFAT4, which is broadly similar to the 3.5-fold slower kinetics seen in the gel-shift assay, suggests NFAT4 is indeed phosphorylated more quickly than NFAT1 within the nucleus.

### Differences in Cytoplasmic Rephosphorylation Rates of NFAT Isoforms

**Figure 6:**
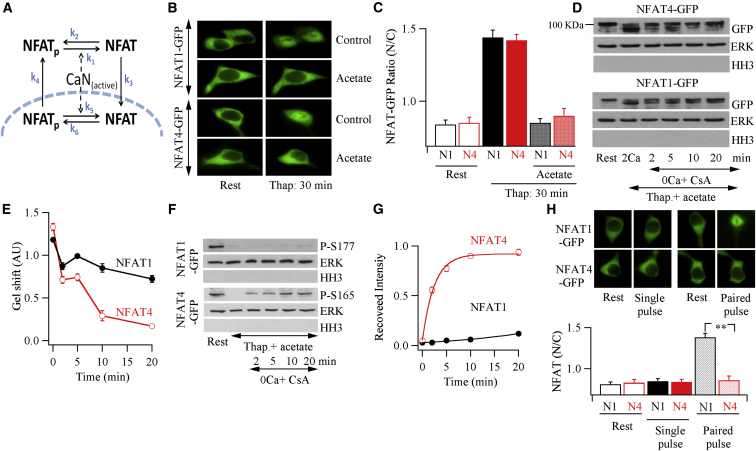
Cytoplasmic Rephosphorylation of NFAT Isoforms (A) The various steps regulating NFAT dynamics are shown. (B) Treatment with acetate (5 mM) inhibits movement of NFAT1-GFP or NFAT4-GFP to the nucleus in response to thapsigargin stimulation. (C) Aggregate data are compared. Each bar represents mean data from three independent experiments. Acetate was applied 10 min before stimulation and then maintained throughout. (D) NFAT isoform rephosphorylation was measured in cytoplasmic extract at different times after termination of calcineurin activity. (E) Graphs summarize averaged data from two independent experiments, as in (D). (F) Blots compare the kinetics of reposhosphorylation of Ser165 on NFAT4 and Ser177 on NFAT1 in cytoplasmic extract. (G) The graph summarizes data from two independent gels, as in (F). Zero intensity at time 0 corresponds to full dephosphorylation of NFAT after thapsigargin stimulation (F). (H) Images compare NFAT1-GFP and NFAT4-GFP nuclear accumulation in experiments using a two-pulse protocol. In the single-pulse protocol, LTC_4_ was applied for 2 min in Ca^2+^-containing external solution (pulse 1) and then washed for 38 min in Ca^2+^-free solution (when the image was taken). In the paired-pulse protocol, after the first pulse, cells were washed for 8 min in Ca^2+^-free solution and then a second identical LTC_4_ pulse was applied for 2 min in external Ca^2+^. Cells were then exposed to Ca^2+^-free solution for a further 28 min before images were taken. Aggregate data from several independent experiments are shown in the histogram. Each bar is the mean of between 7 and 15 cells. In (C), (E), (G), and (H), data are represented as mean ± SEM. See also Figure S6.

Taking advantage of leptomcyin B, our new data in Figure 5 have enabled us to quantify the kinetics of NFAT rephosphorylation within the nucleus (k6 reaction in Figure 6A). We therefore designed experiments to measure NFAT rephosphorylation kinetics in the cytoplasm (k_2_ reaction in Figure 6A). Acetate inhibits NFAT migration into the nucleus by preventing association of the NFAT-importin β complex with tubulin α (Ishiguro et al., 2011). NFAT dephosphorylation proceeded normally, but the dephosphorylated protein was trapped within the cytoplasm (Ishiguro et al., 2011). We reasoned that this approach might enable us to measure the kinetics of NFAT rephosphorylation within the cytoplasm, independent of any nuclear flux. We first confirmed that acetate inhibited translocation of NFAT1 and NFAT4 into the nucleus (Figures 6B and 6C). We then activated CRAC channels with thapsigargin in the presence of acetate to drive NFAT dephosphorylation. After 20 min, a time sufficient for NFAT dephosphorylation (Kar et al., 2011), we rapidly inhibited calcineurin activity by terminating Ca^2+^ influx in the presence of cyclosporine A. This enabled the measure of NFAT rephosphorylation within the cytoplasm (k_2_). NFAT4 was rephosphorylated quickly, with a half-time of ∼4 min (Figures 6D and 6E). By contrast, NFAT1 was rephosphorylated considerably more slowly, with only ∼40% recovery by 20 min (Figures 6D and 6E).

Similar results were seen with the phospho-serine antibodies against NFAT1 and NFAT4. NFAT4 was rephosphorylated significantly more quickly than NFAT1 (Figures 6F and 6G), consistent with the gel-shift data (Figure 6E).

### NFAT1 but Not NFAT4 Nuclear Accumulation Shows Paired-Pulse Facilitation

A consequence of the faster k_2_ reaction for NFAT4 than NFAT1 is that NFAT4 will be rephosphorylated in the cytoplasm considerably more quickly following deactivation of calcineurin. NFAT proteins are extensively phosphorylated by protein kinases (Okamura et al., 2000). A slower k_2_ for NFAT1 means that, following a brief pulse of Ca^2+^ that is insufficient for complete NFAT1 dephosphorylation, the rephosphorylation process would proceed slowly. Hence, a second identical Ca^2+^ pulse a few minutes later should result in further net dephosphorylation and thus complete activation of NFAT1, imparting a form of paired-pulse facilitation to NFAT1 nuclear migration. Our previous experiments demonstrated robust paired-pulse facilitation to NFAT1 (Kar et al., 2012), although the underlying molecular basis was unclear. On the other hand, the faster rate of rephosphorylation of NFAT4 would result in more extensive rephosphorylation during the interpulse interval, and this would not result in additional dephosphorylation when a second identical Ca^2+^ pulse is applied, preventing paired-pulse facilitation. To test this, we measured NFAT4 nuclear dynamics following brief single or paired pulses of LTC_4_. Activation of cysLT1 receptors to a single 2 min pulse failed to induce any detectable NFAT1 or NFAT4 nuclear migration over a subsequent 38 min period (Kar et al., 2012; Figure 6H). However, two identical LTC_4_ pulses, applied 8 min apart, resulted in prominent NFAT1 but not NFAT4 nuclear accumulation 28 min later (38 min after the first pulse; Figure 6H). Similar paired-pulse facilitation was obtained when two identical Ca^2+^ pulses were applied to thapsigargin-treated cells instead (Figure S6).

### Cytokine Expression Is Differentially Regulated by Nuclear Ca^2+^ in a Cell-Specific Context

**Figure 7:**
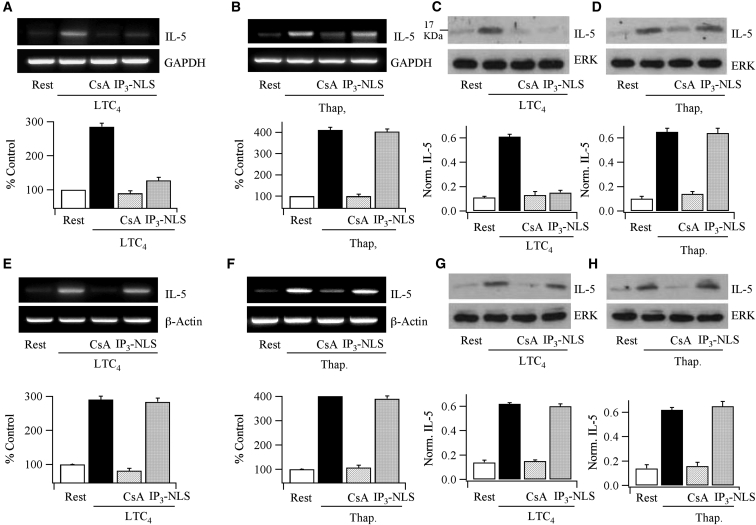
Cytokine Expression Is Regulated By nuclear Ca^2+^ in a Cell-Specific Context Data in (A)–(D) are from 16-HBE bronchial epithelial cells. (A) RT-PCR shows IL-5 expression is increased by LTC_4_ and this is prevented by cyclosporine A or after expression of the IP_3_-NLS buffer. Data have been normalized to rest levels (% control). (B) As in (A), but now thapsigargin is the stimulus. (C) Western blot compares IL-5 protein expression following stimulation with LTC_4_. IL-5 expression has been normalized to ERK levels. (D) As in (C), but the stimulus is thapsigargin. Data in (E)–(H) are from RBL-1 mast cells. (E) LTC_4_ increases IL-5 transcription, and this is unaffected by IP_3_-NLS but suppressed by cyclosporine A. (F) As in (E), but thapsigargin is the stimulus. (G) Western blot compares IL-5 protein expression following stimulation with LTC_4_. (H) As in (F), but thapsigargin is the stimulus. In all panels, histograms summarize data from three independent experiments. In all histograms, data are represented as mean ± SEM.

An important physiological ramification of our findings is that expression of the same gene induced by the same agonist should be differentially regulated by nuclear Ca^2+^ in different cell types, depending on which NFAT protein dominates. 16-HBE bronchial airway epithelial cells express significantly more NFAT4 than NFAT1 (Samanta et al., 2014), whereas the converse is the case in RBL-1 mast cells (data not shown). We therefore stimulated 16-HBE and RBL-1 mast cells with LTC_4_ at a concentration that evoked oscillatory Ca^2+^ signals and measured expression of the interleukin-5 (IL-5) gene, which contains the GGAA core nucleotides for an NFAT binding site within the promoter (De Boer et al., 1999), in the absence or presence of nuclear InsP_3_ buffer. In 16-HBE cells, LTC_4_ stimulation led to a robust increase in transcription of IL-5 (Figure 7A) as well as in protein expression (Figure 7C). Pre-treatment with the calcineurin inhibitor cyclosporine A suppressed these responses (Figures 7A and 7C), demonstrating transcription was through the calcineurin-NFAT pathway. Expression of nuclear InsP_3_ buffer prevented LTC_4_-induced IL-5 transcription and subsequent protein expression (Figures 7A and 7C), consistent with a major role for nuclear Ca^2+^ signaling. By contrast, thapsigargin-evoked expression of IL-5 was unaffected by nuclear InsP_3_ buffer but remained sensitive to cyclosporine A (Figures 7B and 7D). Therefore InsP_3_ nuclear buffer does not interfere with the transcriptional process itself.

In RBL-1 cells, where NFAT1 dominates, LTC_4_ also increased expression of IL-5 (Figures 7E and 7G). These responses were unaffected by nuclear InsP_3_ buffer but were suppressed by cyclosporine A (Figures 7E and 7G). Thapsigargin-induced IL-5 expression was also unaffected by the presence of nuclear InsP_3_ buffer (Figures 7F and 7H).

Although NFAT and c-*fos* often interact synergistically to regulate gene expression, block of the non-receptor tyrosine kinase Syk, a maneuver that abolishes cysLT1 receptor activation of c-*fos* expression (Ng et al., 2012), had no effect on IL-5 gene expression to LTC_4_ in RBL-1 cells (3.73-fold ± 0.10-fold increase in IL-5 mRNA relative to basal following LTC_4_ stimulation in control cells and 3.69-fold ± 0.13-fold increase in the presence of Syk inhibitor, p > 0.1; corresponding changes in protein expression were 6.14 ± 0.42 and 6.29 ± 0.57; p > 0.1). Collectively, the data indicate that NFAT-driven gene expression in different cell types can be differentially regulated by nuclear Ca^2+^. Moreover, the same gene (IL-5) can be differentially controlled by nuclear Ca^2+^, depending on the NFAT isoform present.

### Conclusion

Stimulation of cysLT1 receptors increases cytoplasmic InsP_3_, which lowers the endoplasmic reticulum Ca^2+^ content sufficiently for CRAC channels in the plasma membrane to open, a step necessary and sufficient for NFAT1 activation (Kar et al., 2011, Kar and Parekh, 2015). The closely related NFAT4 protein additionally requires a nuclear Ca^2+^ rise (Kar and Parekh, 2015). Our new data demonstrate that cytoplasmic InsP_3_ diffuses into the nucleus to activate InsP_3_ receptors spanning the inner nuclear membrane, which then increase nucleoplasmic Ca^2+^, enabling prolonged NFAT4 activation. NFAT4 activation is therefore dependent on two spatially segregated Ca^2+^ signals: one at the plasma membrane and the other in the nucleus several micrometers away. The different requirements for nuclear Ca^2+^ and thus nuclear Ca^2+^-calcineurin reflect the different rates of rephosphorylation of NFAT1 and NFAT4 in the nucleus, with rephosphorylation of the latter proceeding several-fold more quickly. The slower rephosphorylation rate of NFAT1 suggests this family member would be considerably better suited for imparting short-term memory to gene expression (Kar et al., 2012). Similar differences in cytoplasmic rephosphorylation rates are also important, playing a major role in the development of paired-pulse facilitation for NFAT1 only.

Remarkably, the spatially restricted Ca^2+^ signals activate NFAT4 without a role for bulk oscillatory Ca^2+^ signals in the relatively vast expanse of the enveloping cytoplasm. A corollary of this is that cytoplasmic Ca^2+^ oscillations, long considered the universal currency of physiological Ca^2+^ signaling, are not essential for the form of receptor-driven excitation-transcription coupling we describe. The oscillatory cytoplasmic Ca^2+^ signal here may be a consequence of the need to deplete the Ca^2+^ stores sufficiently for CRAC channels to activate (Bird et al., 2009, Brandman et al., 2007, Parekh et al., 1997), coupled with other roles such as the enhancement of mitochondrial ATP production that is needed to support energy-dependent Ca^2+^ responses (Hajnóczky et al., 1995).

Our data also show that expression of the same gene in response to the same agonist is controlled differently, depending on which NFAT protein dominates. Whereas NFAT1 activation is governed only by Ca^2+^ microdomains near CRAC channels, a stronger stimulus is needed for NFAT4 nuclear accumulation, because InsP_3_ has to overcome breakdown by cytoplasmic enzymes to enter the nucleus and release nuclear Ca^2+^. The dependence of different NFAT proteins on different stimulus intensities (Kar and Parekh, 2015) will both increase the bandwidth of excitation-transcription coupling and recruit different gene expression profiles. Finally, our results demonstrate how a ubiquitous and globally diffusible second messenger like InsP_3_ can nevertheless be used to activate different transcription factor isoforms through generation of coordinated yet spatially distinct sub-cellular Ca^2+^ signals.

## Experimental Procedures

### Cells

HEK293 (Kar et al., 2011), RBL-1 (Kar et al., 2012), and 16-HBE (Samanta et al., 2014) cells were cultured as described. HEK293 cells were used in Figure 1, Figure 2, Figure 3, Figure 4, Figure 5, Figure 6 and RBL-1 and 16-HBE cells in Figure 7.

### cDNA Constructs and Transfection

HEK293 and 16-HBE cells were transfected using lipofectamine, as described (Kar et al., 2011, Samanta et al., 2014). RBL-1 cells were transfected using the Amaxa system (Kar et al., 2012). cDNA for NFAT4-GFP, parvalbumin constructs, and cytoplasmic GCaMP3 were from Addgene (deposited by Dr. Anjana Rao, Dr. Anton Bennett, and Dr. Jin Zhang, respectively). Nuclear and cytoplasmic InsP_3_ buffer were gifts from Dr. Maria de Fatima Leite, Federal University of Minas Gerais, Brazil. NFAT1-GFP was a gift from Dr. Jennings Worley. Alterations to these constructs were done by Mutagenex. All plasmids were used at 1 μg, and experiments commenced 24–48 hr after transfection.

### NFAT Migration

Tagged NFAT protein levels in the cytoplasm and nucleus were measured using the IMAGO charge-coupled device camera-based system from TILL Photonics, with a × 100 oil immersion objective (numerical aperture 1.3) (Kar and Parekh, 2015). Regions of interest of identical size were drawn in the cytoplasm and nucleus of each cell, and fluorescence was computed. Nuclear localization was confirmed by costaining with the nuclear dye DAPI. Unless otherwise indicated, we calculated the nuclear/cytoplasmic ratio of tagged NFAT as a function of stimulus time.

### Cytoplasmic and Nuclear Ca^2+^ Measurements

Cytoplasmic and nuclear Ca^2+^ measurements, using GCaMP3 constructs, were carried out at room temperature by using the IMAGO charge-coupled device camera-based system from TILL Photonics (Kar et al., 2012). Cells were placed in standard external solution composed of 145 mM NaCl, 2.8 mM KCl, 2 mM CaCl_2_, 2 mM MgCl_2_, 10 mM D-glucose, and 10 mM HEPES at pH 7.4, with NaOH and were excited at 489 nm (typically 100 ms exposure), at 0.5 Hz. Fura-2 experiments (Figure S1) were carried out as described (Kar et al., 2012). Ca^2+^ signals are plotted either as F/F0, which denotes the increase in fluorescence divided by the resting level for the GCaMP3 reporters, or *R*, which denotes the 356/380 nm ratio, for Fura-2. Simultaneous measurements of cytoplasmic and nuclear Ca^2+^ were performed with fluo-4, as described (Kar and Parekh, 2015).

### Immunogold Electron Microscopy

Briefly, cells were fixed with 3% paraformaldehyde/0.05% glutaraldehyde in 0.1 M phosphate buffer for 10 min, stained with uranyl acetate (2% w/v in distilled water), dehydrated through increasing concentrations of methanol (70%–100%), and embedded in LR Gold (London Resin Company). Ultrathin sections (50–80 nm) were prepared by use of a Reichert Ultracut S ultramicrotome (Leica), mounted on 200-mesh nickel grids, incubated at room temperature for 2 hr with a monoclonal anti-InsP_3_ receptor type I antibody (dilution 1:100; Abcam) and for 1 hr with anti-mouse IgG −15 nm gold complex (British Biocell International). All antisera were diluted in 0.1 M phosphate buffer containing 0.1% egg albumin (pH 7.2). As a control the primary antibody was replaced with non-immune sera. After immunolabeling, sections were lightly counterstained with lead citrate and uranyl acetate and examined with a JEOL transmission electron microscope (JEM-1010, JEOL), and images were collected with a Gatan Orius digital camera (Gatan).

### Cytoplasmic Staining

HEK293 cells were fixed with paraformaldehyde (4%) at room temperature and permeabilized with PBS/Triton X-100 (0.5%). Cells were then stained overnight with anti-InsP_3_ receptor type I antibody (Abcam), used at 1:100 dilution. Primary antibody was then visualized with Alexa Fluor 488 goat anti-mouse secondary antibody (1:500)

### Western Blot

Total cell lysates (40 μg) were analyzed by SDS-PAGE on a 10% or 12% (IL-5) gel. Membranes were blocked with 5% nonfat dry milk in PBS plus 0.1% Tween 20 (PBST) buffer for 1 hr at room temperature (Kar and Parekh, 2015). For phospho-NFAT blots, we used TBST (Tris buffer saline with Tween 20). Membranes were washed with PBST three times and then incubated with primary antibody for 24 hr at 4°C. Antibodies against ERK (1:5,000, rabbit polyclonal; Santa Cruz Biotechnology), GFP (1:2,000, rabbit polyclonal; Cell Signaling), HH3 (Histone H3; 1:5,000, rabbit polyclonal; Abcam), InsP_3_ type I receptor (1:1,000, mouse monoclonal; Abcam), anti-phospho Ser177 NFAT1 (Santa Cruz; 1:2,000 dilution), anti-phospho Ser165 NFAT4 (Abcam; 1:2,000), and IL-5 (Santa Cruz; 1:500) were used. Secondary antibodies for rabbit or mouse were used at 1:5,000 or 1:3,000 dilutions. The membranes were then washed with PBST again and incubated with peroxidase-linked anti-rabbit IgG from Santa Cruz Biotechnology or anti-mouse IgG from BD Bioscience for 1 hr at room temperature. After washing with PBST, the bands were detected by an enhanced chemiluminescence (ECL) plus western blotting detection system (Amersham Biosciences). Blots were analyzed by UN-Scan software. Nuclear and cytoplasmic extracts were separated as before (Kar and Parekh, 2015) and assessed by the presence of HH3 in nuclear but not cytoplasmic extract and ERK2 in the cytoplasmic but not nuclear extract.

Gel shifts were quantified in ImageJ. Intensity of the region corresponding to the dephosphorylated state (lower band; obtained after stimulation with thapsigargin in 2 mM external Ca^2+^ for 20 min; see relevant figure legends) was measured and the region (same dimensions and position) was transposed to adjacent lanes. Data are presented relative to the intensity of the dephosphorylated state. Gel analysis was carried out on blots from at least two independent experiments.

### RT-PCR

16-HBE cells transfected with cys LT1 receptors or wild-type RBL-1 cells were stimulated with thapsigargin or LTC_4_ in DMEM for 20 min, washed five times with DMEM (without thapsigargin or LTC_4_), and then kept in an incubator at 37°C for 6–8 hr. Cells were then washed with PBS, and total RNA was extracted using an RNeasy Mini Kit (QIAGEN). RNA was quantified spectrophotometrically by absorbance at 260 nm. Total RNA (1 μg) was reverse transcribed using the iScriptTM cDNA Synthesis Kit (Bio-Rad), according to the manufacturer’s instructions. Two-microliter samples (cDNA), from RT-PCR, were subjected to polymerase chain reaction (PCR) with the specific primers and GoTaq Green master mix (Promega). The mRNA levels were normalized to GAPDH for 16-HBE cell and β-actin for RBL-1 cells.

IL-5 (human): Forward-5′-CTGAGGATTCCTGTTCCTGT-3′; Reverse-5′-CAACTTTCTATTATCCACTC-3′
GAPDH (human): Forward-5′-GGTATCGTGGAAGGACTCAT-3′; Reverse-5′-CCACCCTGTTGCTGTAGCCAAATTC-3′
IL-5 (rat): Forward-5′-AGACCGGTCATACATGCACA-3′; Reverse-5′-TCGTCTCATTGCTCGTCAAC-3′
β-actin (rat): Forward- 5′-TTGTAACCAACTGGGACGATATG-3′; Reverse- 5′-GATCTTGATCTTCATGGTGCTAGG-3′

### Mathematical Modeling

The details of mathematical modeling are explained in the Supplemental Information.

### Statistical Analysis

Data are presented as the mean ± SEM. Statistical significance was determined by using Student’s t test. ^∗^p < 0.05 and ^∗∗^p < 0.01.

## Author Contributions

P.K. and A.B.P. designed experiments. P.K. carried out the experiments. Electron microscopy was conducted by H.C.C. Analyses of data were performed by P.K. and A.B.P. Discussion of the model and how to identify various rate constants involved P.K., G.R.M., and A.B.P. The modeling was executed by G.R.M. A.B.P. wrote the manuscript.

## Accession Numbers

Original imaging data have been deposited to Mendeley Data and are available at http://dx.doi.org/10.17632/v8sfxkh9pw.1.

## Supplemental Information

Document S1. Figures S1–S6, Table S1, and Supplemental Equations

Data S1. Calcineurin Code

Document S2. Article plus Supplemental Information

## References

[bib1] Allbritton N.L., Meyer T., Stryer L. (1992). Range of messenger action of calcium ion and inositol 1,4,5-trisphosphate. Science.

[bib2] Alonso M.T., García-Sancho J. (2011). Nuclear Ca(2+) signalling. Cell Calcium.

[bib3] Bazzazi H., Sang L., Dick I.E., Joshi-Mukherjee R., Yang W., Yue D.T. (2015). Novel fluorescence resonance energy transfer-based reporter reveals differential calcineurin activation in neonatal and adult cardiomyocytes. J. Physiol..

[bib4] Bettelli E., Dastrange M., Oukka M. (2005). Foxp3 interacts with nuclear factor of activated T cells and NF-kappa B to repress cytokine gene expression and effector functions of T helper cells. Proc. Natl. Acad. Sci. USA.

[bib5] Bird G.S., Hwang S.Y., Smyth J.T., Fukushima M., Boyles R.R., Putney J.W.J. (2009). STIM1 is a calcium sensor specialized for digital signaling. Curr. Biol..

[bib6] Bootman M.D., Fearnley C., Smyrnias I., MacDonald F., Roderick H.L. (2009). An update on nuclear calcium signalling. J. Cell Sci..

[bib7] Brandman O., Liou J., Park W.S., Meyer T. (2007). STIM2 is a feedback regulator that stabilizes basal cytosolic and endoplasmic reticulum Ca2+ levels. Cell.

[bib8] De Boer M.L., Mordvinov V.A., Thomas M.A., Sanderson C.J. (1999). Role of nuclear factor of activated T cells (NFAT) in the expression of interleukin-5 and other cytokines involved in the regulation of hemopoetic cells. Int. J. Biochem. Cell Biol..

[bib9] Di Capite J., Ng S.-W., Parekh A.B. (2009). Decoding of cytoplasmic Ca(2+) oscillations through the spatial signature drives gene expression. Curr. Biol..

[bib10] Echevarría W., Leite M.F., Guerra M.T., Zipfel W.R., Nathanson M.H. (2003). Regulation of calcium signals in the nucleus by a nucleoplasmic reticulum. Nat. Cell Biol..

[bib11] Fornerod M., Ohno M., Yoshida M., Mattaj I.W. (1997). CRM1 is an export receptor for leucine-rich nuclear export signals. Cell.

[bib12] Gerasimenko O.V., Gerasimenko J.V., Tepikin A.V., Petersen O.H. (1996). Calcium transport pathways in the nucleus. Pflugers Arch..

[bib13] Gwack Y., Sharma S., Nardone J., Tanasa B., Iuga A., Srikanth S., Okamura H., Bolton D., Feske S., Hogan P.G., Rao A. (2006). A genome-wide Drosophila RNAi screen identifies DYRK-family kinases as regulators of NFAT. Nature.

[bib14] Hajnóczky G., Robb-Gaspers L.D., Seitz M.B., Thomas A.P. (1995). Decoding of cytosolic calcium oscillations in the mitochondria. Cell.

[bib15] Humbert J.P., Matter N., Artault J.C., Köppler P., Malviya A.N. (1996). Inositol 1,4,5-trisphosphate receptor is located to the inner nuclear membrane vindicating regulation of nuclear calcium signaling by inositol 1,4,5-trisphosphate. Discrete distribution of inositol phosphate receptors to inner and outer nuclear membranes. J. Biol. Chem..

[bib16] Ishiguro K., Ando T., Maeda O., Watanabe O., Goto H. (2011). Cutting edge: tubulin α functions as an adaptor in NFAT-importin β interaction. J. Immunol..

[bib17] Kar P., Parekh A.B. (2015). Distinct spatial Ca2+ signatures selectively activate different NFAT transcription factor isoforms. Mol. Cell.

[bib18] Kar P., Nelson C., Parekh A.B. (2011). Selective activation of the transcription factor NFAT1 by calcium microdomains near Ca2+ release-activated Ca2+ (CRAC) channels. J. Biol. Chem..

[bib19] Kar P., Nelson C., Parekh A.B. (2012). CRAC channels drive digital activation and provide analog control and synergy to Ca(2+)-dependent gene regulation. Curr. Biol..

[bib20] Kehlenbach R.H., Dickmanns A., Gerace L. (1998). Nucleocytoplasmic shuttling factors including Ran and CRM1 mediate nuclear export of NFAT In vitro. J. Cell Biol..

[bib21] Lin C., Hajnóczky G., Thomas A.P. (1994). Propagation of cytosolic calcium waves into the nuclei of hepatocytes. Cell Calcium.

[bib22] Lipp P., Thomas D., Berridge M.J., Bootman M.D. (1997). Nuclear calcium signalling by individual cytoplasmic calcium puffs. EMBO J..

[bib23] Loh C., Shaw K.T.-Y., Carew J., Viola J.P.B., Luo C., Perrino B.A., Rao A. (1996). Calcineurin binds the transcription factor NFAT1 and reversibly regulates its activity. J. Biol. Chem..

[bib24] Macián F., López-Rodríguez C., Rao A. (2001). Partners in transcription: NFAT and AP-1. Oncogene.

[bib25] Macián F., García-Cózar F., Im S.H., Horton H.F., Byrne M.C., Rao A. (2002). Transcriptional mechanisms underlying lymphocyte tolerance. Cell.

[bib26] Martinez G.J., Pereira R.M., Äijö T., Kim E.Y., Marangoni F., Pipkin M.E., Togher S., Heissmeyer V., Zhang Y.C., Crotty S. (2015). The transcription factor NFAT promotes exhaustion of activated CD8^+^ T cells. Immunity.

[bib27] Mogami H., Nakano K., Tepikin A.V., Petersen O.H. (1997). Ca2+ flow via tunnels in polarized cells: recharging of apical Ca2+ stores by focal Ca2+ entry through basal membrane patch. Cell.

[bib28] Müller M.R., Rao A. (2010). NFAT, immunity and cancer: a transcription factor comes of age. Nat. Rev. Immunol..

[bib29] Ng S.W., Bakowski D., Nelson C., Mehta R., Almeyda R., Bates G., Parekh A.B. (2012). Cysteinyl leukotriene type I receptor desensitization sustains Ca2+-dependent gene expression. Nature.

[bib30] Okamura H., Aramburu J., García-Rodríguez C., Viola J.P., Raghavan A., Tahiliani M., Zhang X., Qin J., Hogan P.G., Rao A. (2000). Concerted dephosphorylation of the transcription factor NFAT1 induces a conformational switch that regulates transcriptional activity. Mol. Cell.

[bib31] Parekh A.B., Fleig A., Penner R. (1997). The store-operated calcium current I(CRAC): nonlinear activation by InsP3 and dissociation from calcium release. Cell.

[bib32] Rao A., Luo C., Hogan P.G. (1997). Transcription factors of the NFAT family: regulation and function. Annu. Rev. Immunol..

[bib33] Samanta K., Bakowski D., Parekh A.B. (2014). Key role for store-operated Ca2+ channels in activating gene expression in human airway bronchial epithelial cells. PLoS ONE.

[bib34] Saucerman J.J., Bers D.M. (2008). Calmodulin mediates differential sensitivity of CaMKII and calcineurin to local Ca2+ in cardiac myocytes. Biophys. J..

[bib35] Thomas A.P., Bird G.S., Hajnóczky G., Robb-Gaspers L.D., Putney J.W.J. (1996). Spatial and temporal aspects of cellular calcium signaling. FASEB J..

[bib36] Yissachar N., Sharar Fischler T., Cohen A.A., Reich-Zeliger S., Russ D., Shifrut E., Porat Z., Friedman N. (2013). Dynamic response diversity of NFAT isoforms in individual living cells. Mol. Cell.

[bib37] Zima A.V., Bare D.J., Mignery G.A., Blatter L.A. (2007). IP3-dependent nuclear Ca2+ signalling in the mammalian heart. J. Physiol..

