# Supplementary material for: Control of NFAT Isoform Activation and NFAT-Dependent Gene Expression through Two Coincident and Spatially Segregated Intracellular Ca2+ Signals
Source: Mol Cell. 2016 Nov 17;64(4):746–59. doi: 10.1016/j.molcel.2016.11.011 (PMC5128683; doi:10.1016/j.molcel.2016.11.011)
Supplement: Document S1. Figures S1–S6, Table S1, and Supplemental Equations [file mmc1.pdf]

Molecular Cell, Volume *64*

Supplemental Information

Control of NFAT Isoform Activation and NFAT-Dependent Gene Expression through Two Coincident and Spatially Segregated Intracellular Ca^{2+} Signals

Pulak Kar, Gary R. Mirams, Helen C. Christian, and Anant B. Parekh

Figure S1

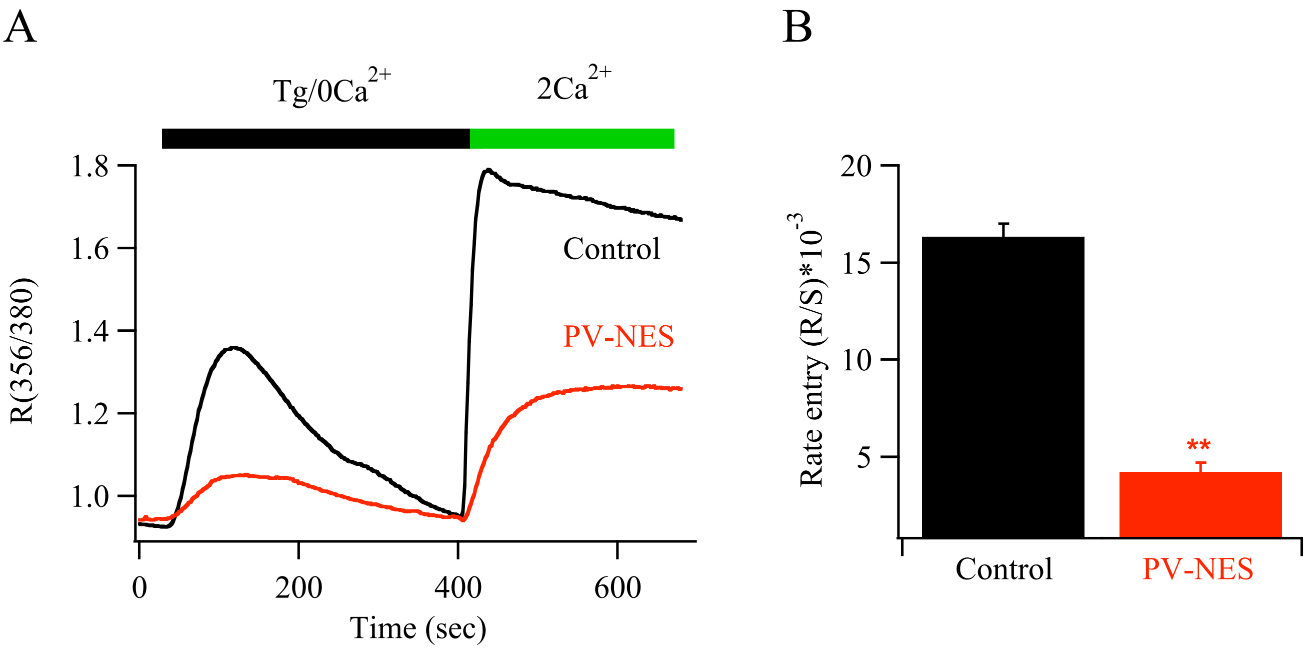
A
Tg/0Ca2+
2Ca2+
1.8
1.6
1.4
1.2
1.0
R(356/380)
Control
PV-NES
0
200
400
600
Time (sec)
B
20
15
10
5
Rate entry (R/S)*10-3
**
Control
PV-NES

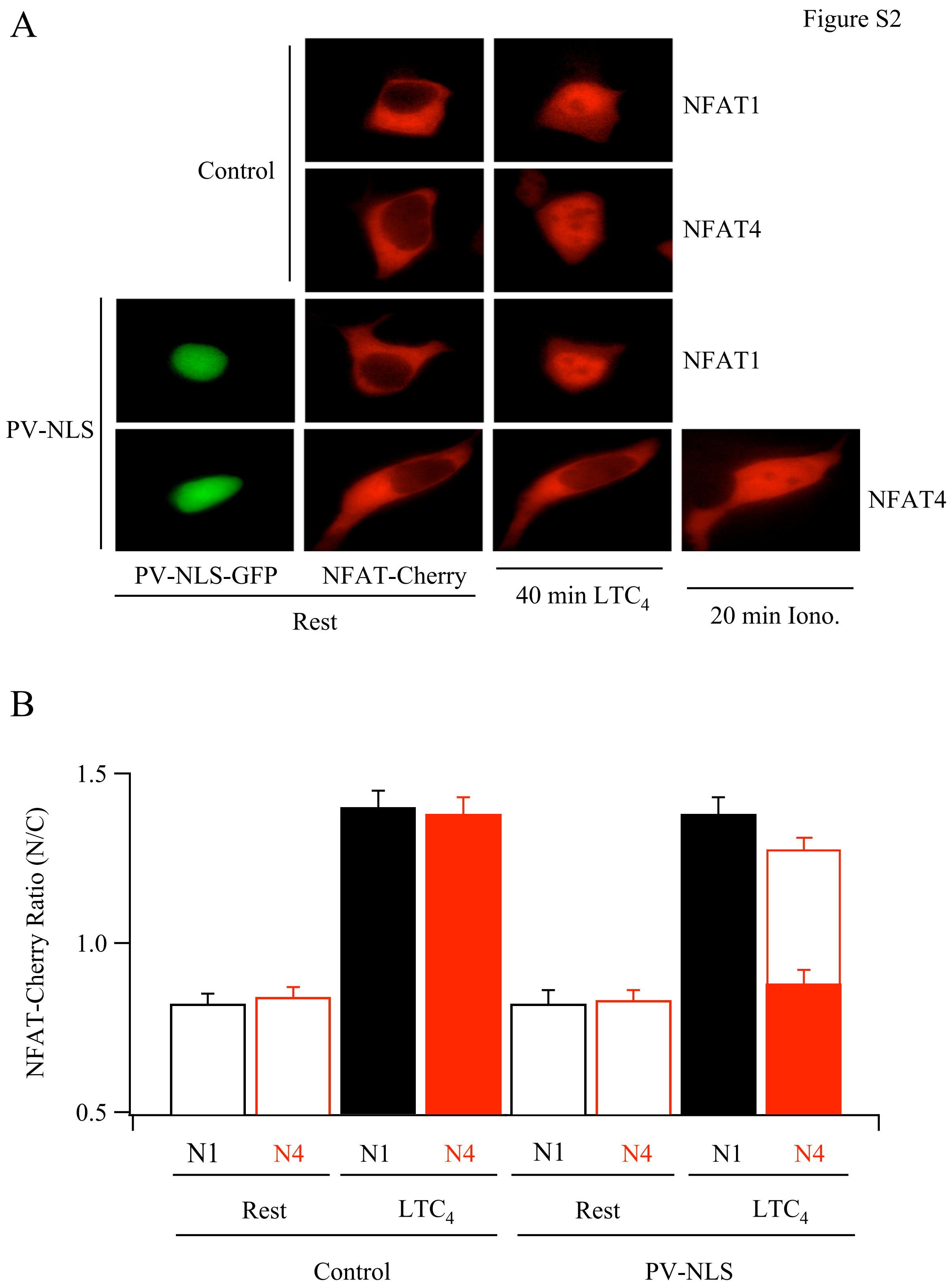

A
Control
NFAT1
NFAT4
PV-NLS
NFAT1
NFAT4
PV-NLS-GFP
NFAT-Cherry
Rest
40 min LTC4
20 min Iono.
B
NFAT-Cherry Ratio (N/C)
1.5
1.0
0.5
N1
N4
N1
N4
N1
N4
N1
N4
Rest
LTC4
Rest
LTC4
Control
PV-NLS

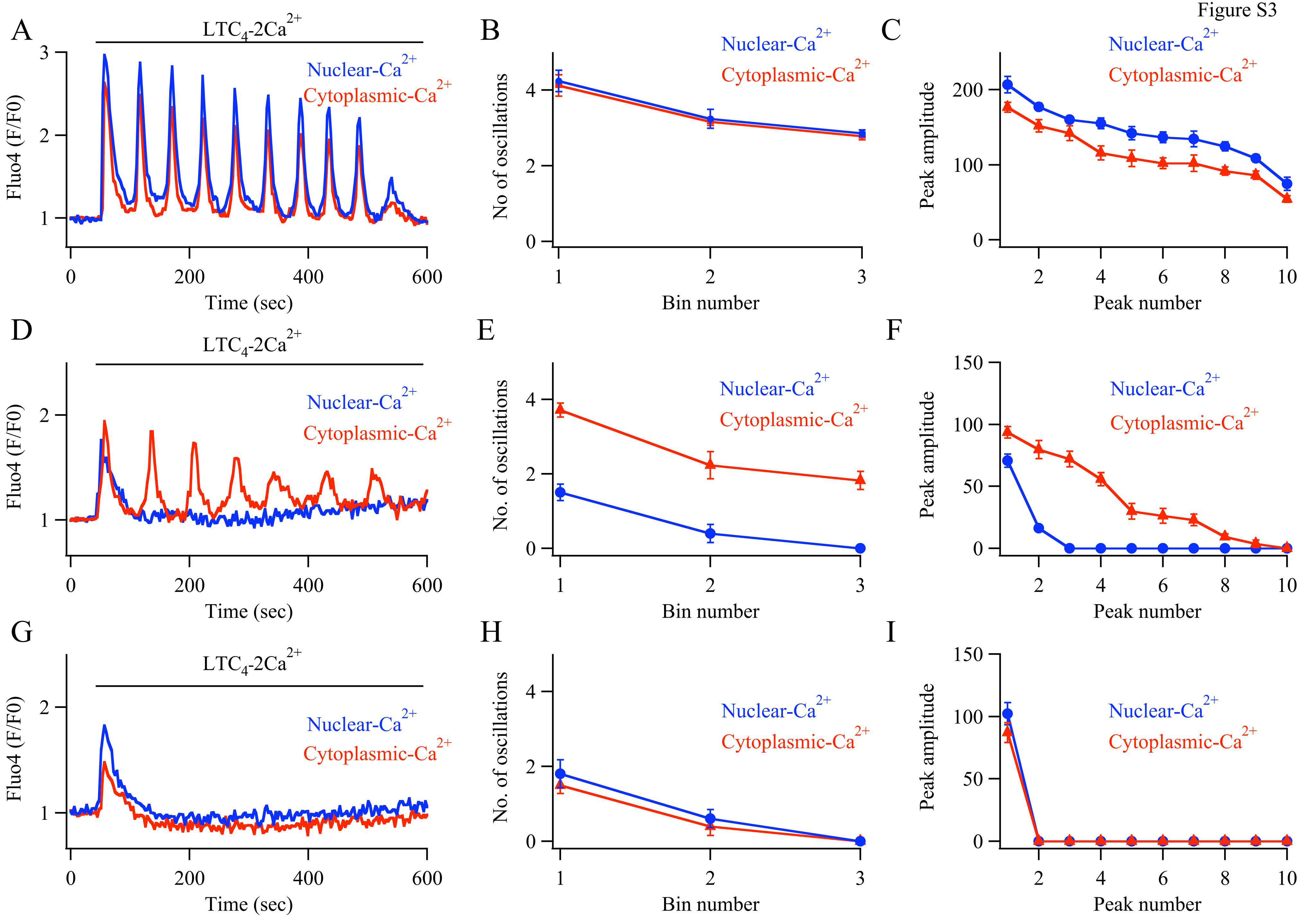
A
LTC$_4$-2Ca^{2+}
Nuclear-Ca^{2+}
Cytoplasmic-Ca^{2+}
Fluo4 (F/F0)
Time (sec)
B
No of oscillations
Bin number
C
Peak amplitude
Peak number
D
E
No. of oscillations
F
G
H
I

Figure S4

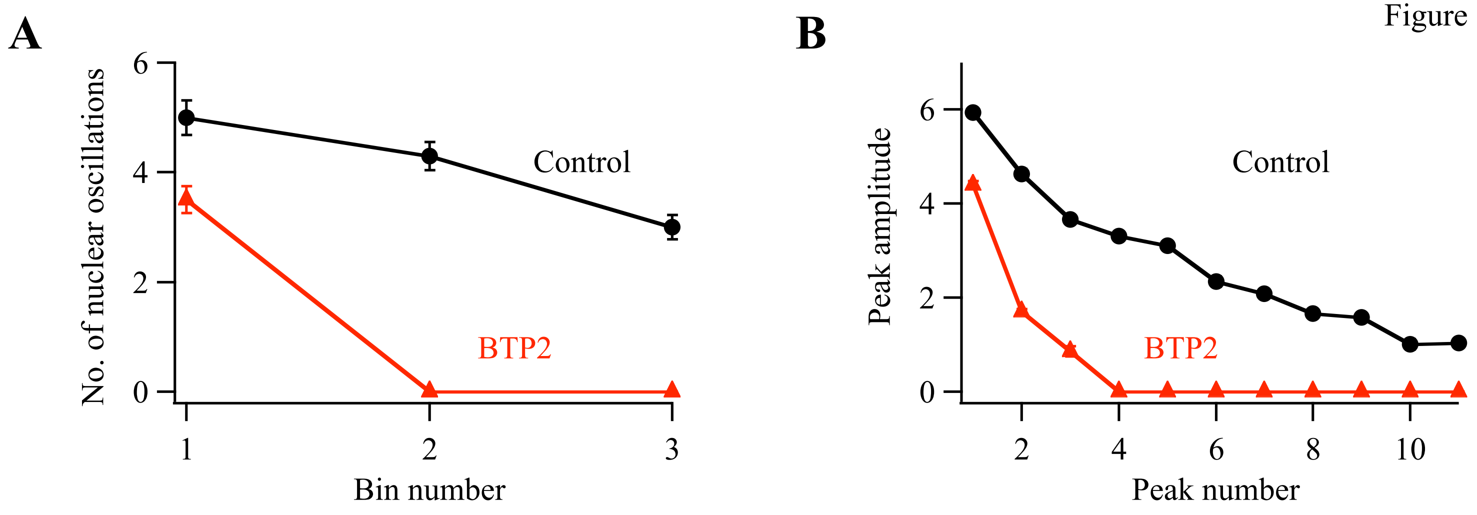
A
No. of nuclear oscillations
6
4
2
0
1
2
3
Bin number
Control
BTP2
B
Peak amplitude
6
4
2
0
2
4
6
8
10
Peak number
Control
BTP2

Figure S5

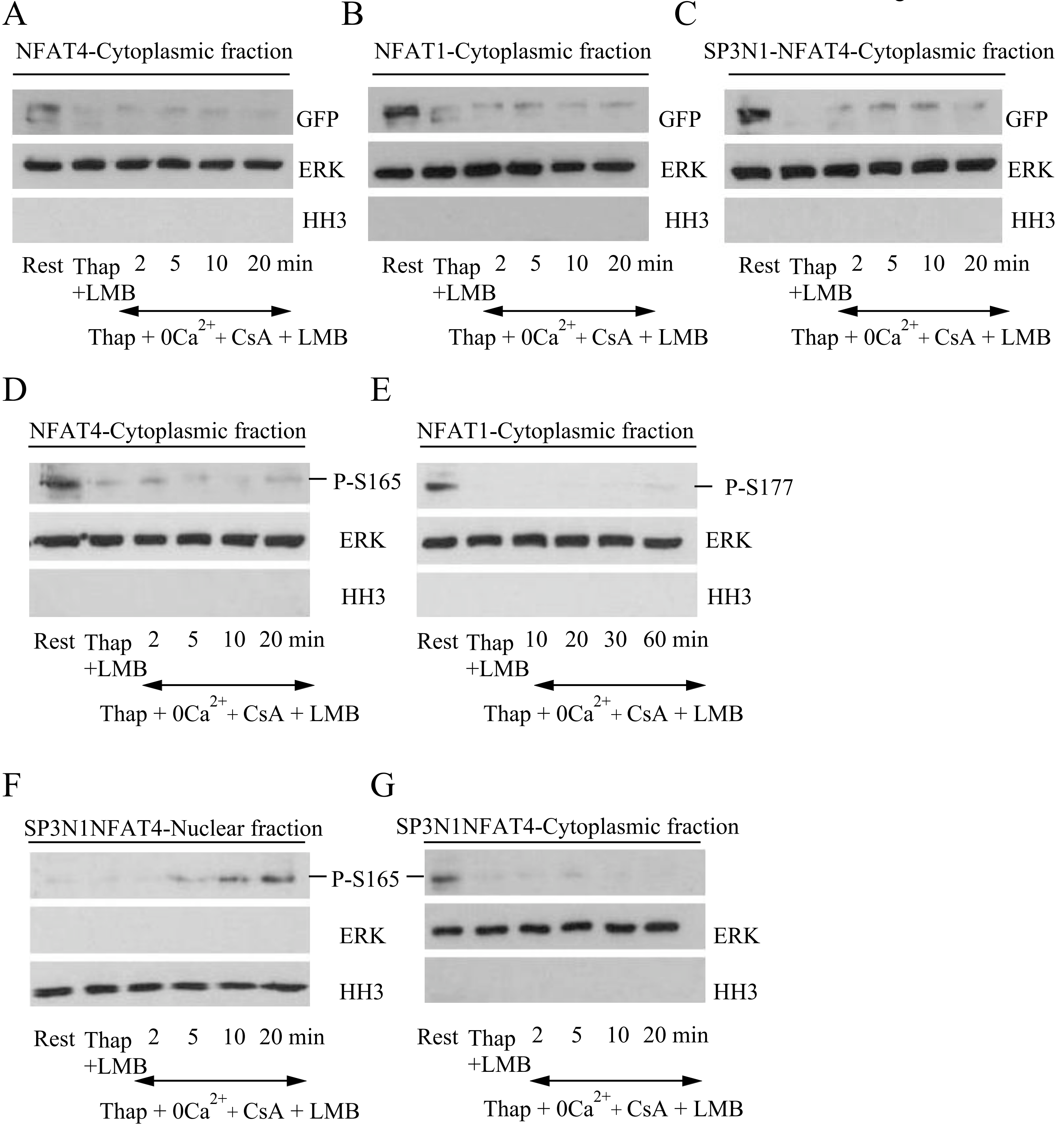
A
NFAT4-Cytoplasmic fraction
GFP
ERK
HH3
Rest Thap +LMB 2 5 10 20 min
Thap + 0Ca2+ + CsA + LMB
B
NFAT1-Cytoplasmic fraction
GFP
ERK
HH3
Rest Thap +LMB 2 5 10 20 min
Thap + 0Ca2+ + CsA + LMB
C
SP3N1-NFAT4-Cytoplasmic fraction
GFP
ERK
HH3
Rest Thap +LMB 2 5 10 20 min
Thap + 0Ca2+ + CsA + LMB
D
NFAT4-Cytoplasmic fraction
P-S165
ERK
HH3
Rest Thap +LMB 2 5 10 20 min
Thap + 0Ca2+ + CsA + LMB
E
NFAT1-Cytoplasmic fraction
P-S177
ERK
HH3
Rest Thap +LMB 10 20 30 60 min
Thap + 0Ca2+ + CsA + LMB
F
SP3N1NFAT4-Nuclear fraction
P-S165
ERK
HH3
Rest Thap +LMB 2 5 10 20 min
Thap + 0Ca2+ + CsA + LMB
G
SP3N1NFAT4-Cytoplasmic fraction
ERK
HH3
Rest Thap +LMB 2 5 10 20 min
Thap + 0Ca2+ + CsA + LMB

Figure S6

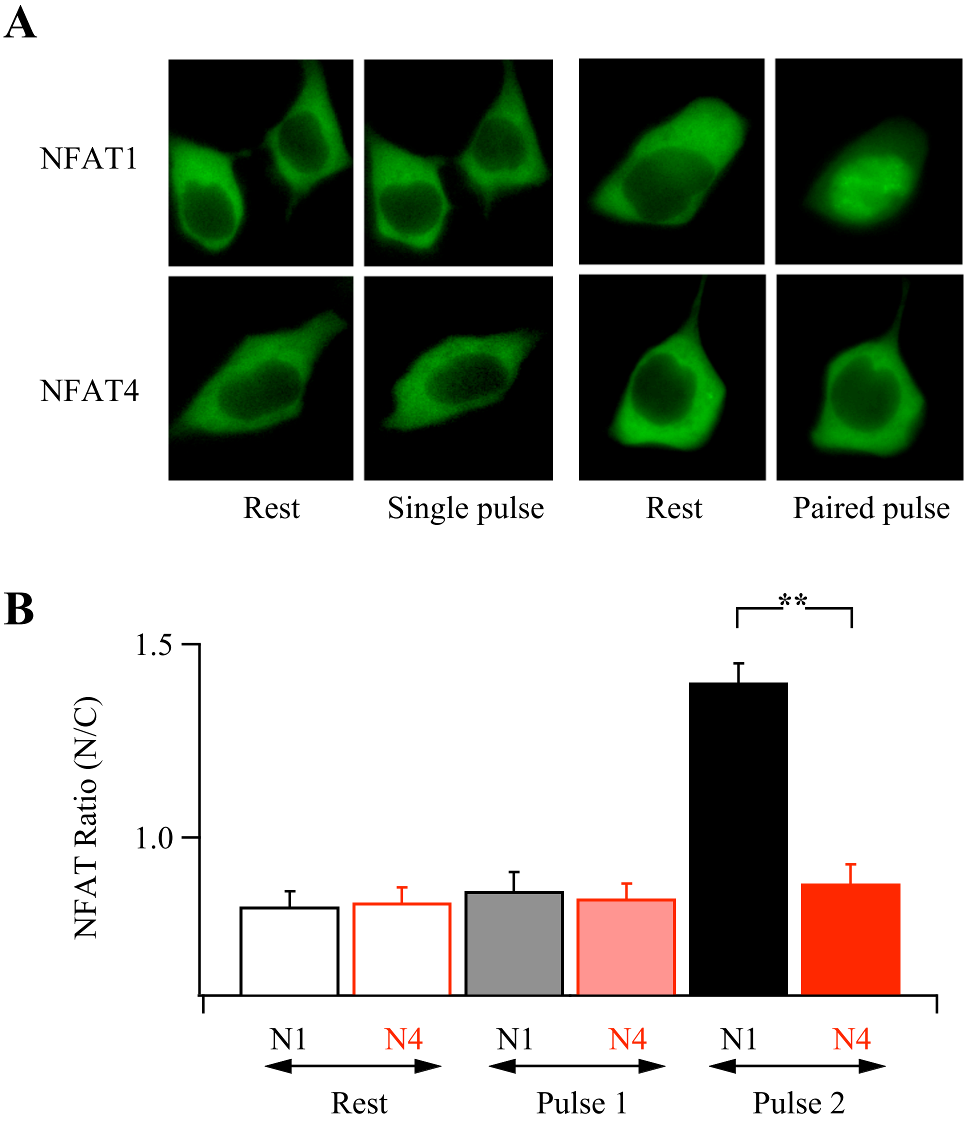
A
NFAT1
NFAT4
Rest
Single pulse
Rest
Paired pulse
B
NFAT Ratio (N/C)
1.5
1.0
**
N1
N4
N1
N4
N1
N4
Rest
Pulse 1
Pulse 2

Figure S1, related to Figure 1. PV-NES reduces intracellular Ca^{2+} signals in fura 2-loaded cells. A, Expression of parvalbumin tagged with a nuclear export sequence (PV-NES) reduces the cytoplasmic Ca^{2+} rise following both Ca^{2+} release and then Ca^{2+} influx after thapsigargin stimulation. Thapsigargin was applied in Ca^{2+}-free solution (labelled Tg/0Ca^{2+}). B, The rate of rise of cytoplasmic Ca^{2+} following Ca^{2+} readmission to cells pre-treated with thapisgargin (2 μM) for 10 minutes in Ca^{2+}-free external solution is compared between control (mock-transfected) cells and cells expressing PV-NES. Each bar is the average of > 26 cells. ** denotes $p<0.01$. In Panel B, data are represented as mean±SEM.

Figure S2, related to Figure 1. Nuclear-targeted parvalbumin (PV-NLS) inhibits NFAT4 but not NFAT1 nuclear accumulation. A, Images compare NFAT nuclear migration between control cells and cells expressing PV-NLS. Upper images, marked control, show the distribution of NFAT1- or NFAT4-cherry at rest, and then after stimulation with LTC_4 (160 nM for 40 minutes). Nuclear migration occurs for both NFAT proteins. The lower images show the effects of PV-NLS-GFP on NFAT-cherry migration. PV-NLS-GFP localized to the nucleus (left hand panels) and NFAT1-cherry or NFAT4-cherry was cytoplasmic at rest. LTC_4 induced movement of NFAT1 but not NFAT4 in cells expressing PV-NLS, both measured 40 minutes after stimulation. Movement of NFAT4 was subsequently recovered by ionomycin application (20 minutes; 2 μM). B, Aggregate data from several experiments as in Panel A are summarised. N1 denotes NFAT1 and N4 NFAT4. Each bar is the mean of between 9 and 17 cells. Open bar above LTC_4/N4 bar in PV-NLS shows the extent of rescue by ionomycin in these cells, applied 40 minutes after LTC_4. In Panel B, data are represented as mean±SEM.

Figure S3, related to Figure 3. Simultaneous measurements of cytoplasmic and nuclear Ca^{2+} signals in fluo-4-loaded cells. A, Ca^{2+} oscillations in the cytoplasm and nucleus to LTC_4 in a control cell are shown. B-C, Aggregate data comparing the number of oscillations per 200 seconds bin (B) and peak amplitude of each oscillation (C) are

depicted. D-F, As in panels A-C but cells now expressed IP_3-NLS. G-I, As in panels A-C but cells expressed both IP_3-NES and IP_3-NLS. In the graphs, data are represented as mean±SEM.

Figure S4, related to Figure 4. Nuclear Ca^{2+} oscillations induced by LTC_4 run down quickly when CRAC channels are blocked. A, Aggregate data compare the number of oscillations per 200 seconds bin for the conditions shown. B, The peak amplitude of each oscillation in control cells and cells exposed to BTP2 are compared. Each point is the mean of between 17 and 25 cells. Cells were exposed to 10 μM BTP2 for 10 minutes prior to stimulation with LTC_4. In the graphs, data are represented as mean±SEM.

Figure S5, related to Figure 5. Leptomycin B (LMB) traps NFAT in the nucleus for up to 20 minutes. A, Gel shows that after stimulation with thapsigargin in LMB, very little NFAT4-GFP remains in the cytoplasmic fraction. Even after initiation of nuclear export (Ca^{2+}-free external solution containing cyclosporine A), very little NFAT4-GFP returns to the cytoplasm in the presence of LMB for up to 20 minutes. B, As in panel A but NFAT1-GFP was expressed instead. C, As in panel A but the SP3N1-NFAT4-GFP construct was expressed instead. D, Phosphorylated serine 165 on NFAT4 is prominent in the cytoplasmic fraction at rest but very little remains in the cytoplasm after stimulation with thapsigargin in LMB and no detectable recovery of cytoplasmic phosphorylated serine 165 occurs after nuclear export is initiated in the presence of LMB. E, As in panel D but NFAT1 was expressed and phosphorylation was assessed on serine 177. F, Nuclear extract from cells expressing SP3N1-NFAT4 shows undetectable phosphorylated serine 165 at rest or after thapsigargin stimulation (30 minutes in external Ca^{2+}), but a time-dependent increase following exposure to Ca^{2+}-free external solution supplemented with cyclosporine A in LMB. G, As in panel F but the cytoplasmic fraction is shown. Note the lack of SP3N1-NFAT4 in the cytoplasm for up to 20 minutes in the presence of LMB after nuclear export had been initiated, confirming LMB blocks nuclear exit.

Figure S6, related to Figure 6. NFAT1 but not NFAT4 exhibits paired pulse facilitation to Ca^{2+} pulses following thapsigargin stimulation. A, Images compare NFAT1-GFP and NFAT4-GFP distribution at rest,

after one Ca^{2+} pulse (single pulse) and after a paired pulse protocol. Cells were pre-treated with thapsigargin in Ca^{2+}-free solution for 15 minutes to deplete stores. Single pulse denotes NFAT distribution following a Ca^{2+} pulse for two minutes followed by exposure to Ca^{2+}-free solution for 38 minutes. Paired pulse represents two identical pulses (two minutes each), spaced 8 minutes apart. Cells were in Ca^{2+}-free solution during the interpulse interval and then for 28 minutes after the second pulse had been applied (total of 38 minutes after the first pulse). B, Aggregate data for the conditions indicated are compared. ** denotes $p < 0.01$. N1 denotes NFAT1 and N4 NFAT4. Data are represented as mean±SEM.

Table S1, related to Figure 4: Parameter values for mathematical model, these are taken from Saucerman et al. (2008) and we keep the same notation, that paper also provides literature references for these values. Note $[CaN]_{Total}$ is varied in some of our simulations, and $[Ca^{2+}]$ varies as an input into all simulations.

Rate constant	Value	Units
k_{20}	10	s^{-1}
K_{d02}	3.1	μM
k_{02}	k_{20}/K_{d02}	$\mu M^{-2}\ s^{-1}$
k_{42}	500	s^{-1}
K_{d24}	24	μM
k_{24}	k_{42}/K_{d24}	$\mu M^{-2}\ s^{-1}$
$k_{CaN4Off}$	2.0	s^{-1}
k_{CaN4On}	46	$\mu M^{-1}\ s^{-1}$
$k_{CaNCaOff}$	1.0	s^{-1}
$k_{CaNCaOn}$	$k_{CaNCaOff}/0.5$	$\mu M^{-2}\ s^{-1}$
$[CaN]_{Total}$	3.62	μM
$[CaM]_{Total}$	6	μM

Zipped data file (related to Figure 4) executes the model based on the parameters in Table 1.The model, in cartoon form, is:

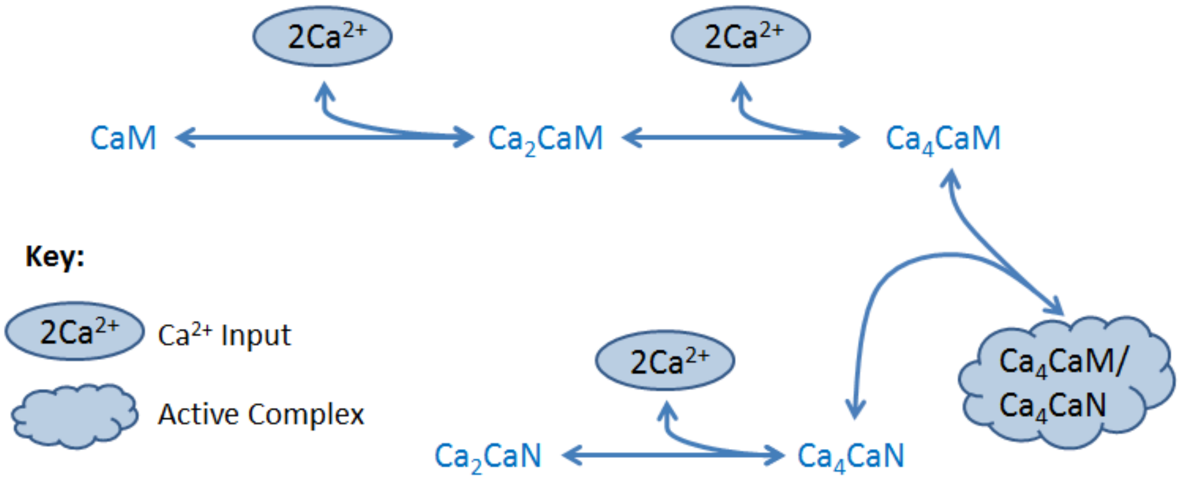

This schematic is the reduced version of the Saucerman et al. (2008), as used by Bazzazi et al. (2015).

The following equations were proposed by Bazzazi et al. (2015) as a simplification of those found in an earlier model of calcineurin activation (Saucerman & Bers 2008). We have followed Bazzazi et al. in using these simpler equations. The equations are derived using mass-action kinetics, with two conservation relationships eliminating the ordinary differential equations (ODEs) for [CaM] and [Ca_2CaN], leaving the following differential & algebraic equation (DAE) system:

$$
\begin{aligned}
[\mathrm{CaM}] &= [\mathrm{CaM}]_{\mathrm{Total}} - \left([\mathrm{Ca_2CaM}] + [\mathrm{Ca_4CaM}] + [\mathrm{Ca_4CaMCa_4CaN}]\right), \\
[\mathrm{Ca_2CaN}] &= [\mathrm{CaN}]_{\mathrm{Total}} - \left([\mathrm{Ca_4CaN}] + [\mathrm{Ca_4CaMCa_4CaN}]\right), \\
\frac{\mathrm{d}[\mathrm{Ca_2CaM}]}{\mathrm{d}t} &= k_{02}[\mathrm{CaM}][\mathrm{Ca^{2+}}]^2 - k_{20}[\mathrm{Ca_2CaM}] + k_{42}[\mathrm{Ca_4CaM}] - k_{24}[\mathrm{Ca_2CaM}][\mathrm{Ca^{2+}}]^2, \\
\frac{\mathrm{d}[\mathrm{Ca_4CaM}]}{\mathrm{d}t} &= k_{24}[\mathrm{Ca^{2+}}]^2[\mathrm{Ca_2CaM}] - k_{42}[\mathrm{Ca_4CaM}] + k_{CaN4Off}[\mathrm{Ca_4CaMCa_4CaN}] \\
&\quad - k_{CaN4On}[\mathrm{Ca_4CaM}][\mathrm{Ca_4CaN}], \\
\frac{\mathrm{d}[\mathrm{Ca_4CaN}]}{\mathrm{d}t} &= k_{CaNCaOn}[\mathrm{Ca^{2+}}]^2[\mathrm{Ca_2CaN}] - k_{CaNCaOff}[\mathrm{Ca_4CaN}] \\
&\quad + k_{CaN4Off}[\mathrm{Ca_4CaMCa_4CaN}] - k_{CaN4On}[\mathrm{Ca_4CaM}][\mathrm{Ca_4CaN}], \\
\frac{\mathrm{d}[\mathrm{Ca_4CaMCa_4CaN}]}{\mathrm{d}t} &= k_{CaN4On}[\mathrm{Ca_4CaM}][\mathrm{Ca_4CaN}] - k_{CaN4Off}[\mathrm{Ca_4CaMCa_4CaN}].
\end{aligned}
$$

Here, square brackets denote concentrations, and rate parameter values are given in Table S1, using the same notation and parameter values as Saucerman & Bers (2008).

References

Bazzazi, H. et al., (2015). Novel fluorescence resonance energy transfer-based reporter reveals differential calcineurin activation in neonatal and adult cardiomyocytes. *The Journal of Physiology* 593, 3865–84.

Kar, P. and Parekh, A. B. (2015). Distinct spatial Ca2+ signatures selectively activate different NFAT transcription factor isoforms. *Molecular Cell* 58, 232-243.

Saucerman, J.J. & Bers, D.M. (2008). Calmodulin mediates differential sensitivity of CaMKII and calcineurin to local Ca2+ in cardiac myocytes. *Biophysical Journal* 95, 4597–612.

Tomida T, Hirose K, Takizawa A, Shibasaki F, Iino M. (2003). NFAT functions as a working memory of Ca2+ signals in decoding Ca2+ oscillation. *EMBO Journal* 22, 3825–32.
