# Supplementary material for: Control of NFAT Isoform Activation and NFAT-Dependent Gene Expression through Two Coincident and Spatially Segregated Intracellular Ca2+ Signals
Source: Mol Cell. 2016 Nov 17;64(4):746–59. doi: 10.1016/j.molcel.2016.11.011 (PMC5128683; doi:10.1016/j.molcel.2016.11.011)
Supplement: Document S2. Article plus Supplemental Information [file mmc3.pdf]

Molecular Cell

Article

Control of NFAT Isoform Activation and NFAT-Dependent Gene Expression through Two Coincident and Spatially Segregated Intracellular Ca^{2+} Signals

Graphical Abstract

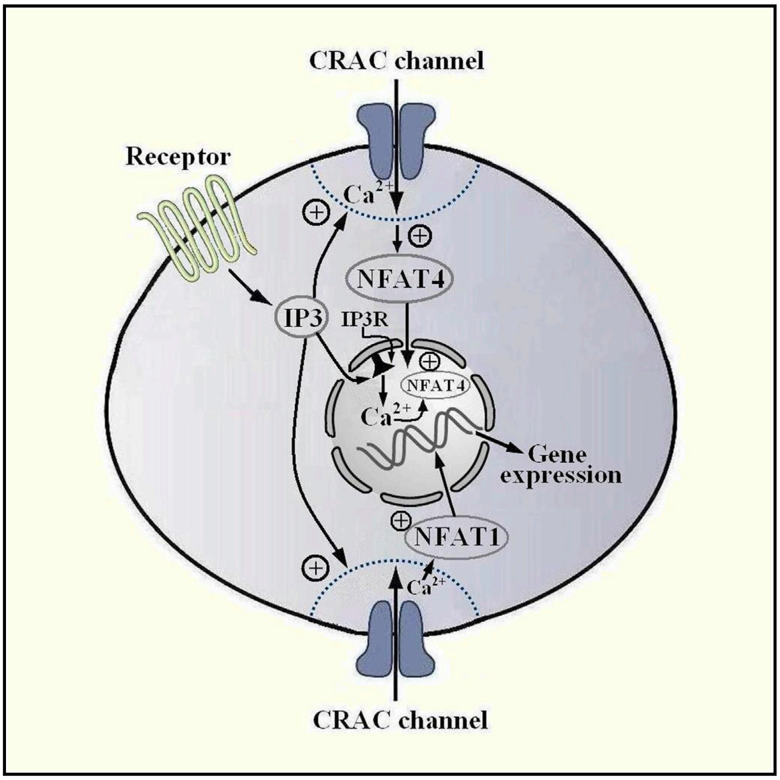

Highlights

- NFAT1 is activated by local Ca^{2+} entry, whereas NFAT4 also needs nuclear Ca^{2+}
- Nuclear Ca^{2+} increases via $InsP_3$ receptors located on the inner nuclear membrane
- NFAT1 and NFAT4 show very different rephosphorylation (deactivation) kinetics
- Slow deactivation of NFAT1 affords a form of short-term memory to gene expression

Authors

Pulak Kar, Gary R. Mirams, Helen C. Christian, Anant B. Parekh

Correspondence

anant.parekh@dpag.ox.ac.uk

In Brief

NFAT1 and NFAT4 are widely coexpressed transcription factors. NFAT1 is activated by Ca^{2+} microdomains near CRAC channels, whereas NFAT4 additionally requires nuclear Ca^{2+}. Kar et al. now show that nuclear Ca^{2+} is raised by $InsP_3$ receptors on the inner nuclear membrane. The dependence of NFAT isoforms on distinct sub-cellular Ca^{2+} signals regulates gene expression in cell- and stimulus-specific contexts.

CrossMark

Kar et al., 2016, Molecular Cell *64*, 746–759
November 17, 2016 © 2016 The Author(s). Published by Elsevier Inc.
http://dx.doi.org/10.1016/j.molcel.2016.11.011

CellPress

CellPress

Molecular Cell
Article

Control of NFAT Isoform Activation and NFAT-Dependent Gene Expression through Two Coincident and Spatially Segregated Intracellular Ca^{2+} Signals

Pulak Kar,[1] Gary R. Mirams,[2] Helen C. Christian,[1] and Anant B. Parekh[1,3,*]
[1]Department of Physiology, Anatomy and Genetics, University of Oxford, Parks Road, Oxford, OX1 3PT, UK
[2]Computational Biology, Department of Computer Science, University of Oxford, Parks Road, Oxford, OX1 3QD, UK
[3]Lead Contact
*Correspondence: anant.parekh@dpag.ox.ac.uk
http://dx.doi.org/10.1016/j.molcel.2016.11.011

SUMMARY

Excitation-transcription coupling, linking stimulation at the cell surface to changes in nuclear gene expression, is conserved throughout eukaryotes. How closely related coexpressed transcription factors are differentially activated remains unclear. Here, we show that two Ca^{2+}-dependent transcription factor isoforms, NFAT1 and NFAT4, require distinct subcellular $InsP_3$ and Ca^{2+} signals for physiologically sustained activation. NFAT1 is stimulated by subplasmalemmal Ca^{2+} microdomains, whereas NFAT4 additionally requires Ca^{2+} mobilization from the inner nuclear envelope by nuclear $InsP_3$ receptors. NFAT1 is rephosphorylated (deactivated) more slowly than NFAT4 in both cytoplasm and nucleus, enabling a more prolonged activation phase. Oscillations in cytoplasmic Ca^{2+}, long considered the physiological form of Ca^{2+} signaling, play no role in activating either NFAT protein. Instead, effective sustained physiological activation of NFAT4 is tightly linked to oscillations in nuclear Ca^{2+}. Our results show how gene expression can be controlled by coincident yet geographically distinct Ca^{2+} signals, generated by a freely diffusible $InsP_3$ message.

INTRODUCTION

Proteins of the NFAT family of transcription factors regulate expression of a multitude of genes that are essential for vertebrate development and function (Müller and Rao, 2010). The family consists of five members, of which four (NFAT1–NFAT4) are activated by intracellular Ca^{2+} signals. In resting cells, NFAT proteins are extensively phosphorylated and thereby trapped within the cytoplasm. Upon stimulation of cell-surface receptors that increase cytoplasmic Ca^{2+}, the proteins are dephosphorylated by Ca^{2+}-calmodulin-activated calcineurin, the target for immunosuppressants cyclosporine A and tacrolimus. Dephosphorylation of cytoplasmic NFAT exposes a lysine-rich nuclear localization sequence, enabling the transcription factor to migrate into the nucleus, in combination with the protein importin β (Rao et al., 1997). Nuclear export occurs after rephosphorylation by nuclear resident protein kinases, which unmasks a nuclear export sequence (Gwack et al., 2006), enabling CRM1 and Ran-GTP nuclear shuttle proteins to transport NFAT across the nuclear envelope via the nuclear pore complex (Kehlenbach et al., 1998).

Once within the nucleus, NFATs regulate gene transcription, both alone and in combination with other transcription factors such as the AP-1 complex and FOXP3 (Bettelli et al., 2005; Macián et al., 2001). Whether NFAT proteins cooperate with AP-1 in particular has important functional consequences. Studies with an engineered NFAT1 protein that was unable to interact with AP-1 led to anergy and exhaustion in CD4+ and CD8+ T cells (Macián et al., 2002; Martinez et al., 2015). By contrast, NFAT/AP-1 cooperation resulted in a productive immune response. In addition to this form of protein-protein crosstalk, we have recently found that NFAT1 and NFAT4, two homologs that are often coexpressed in cells and within the same spatial domain, are regulated by different patterns of cytoplasmic Ca^{2+} signal (Kar and Parekh, 2015). Ca^{2+} microdomains near open store-operated Ca^{2+} release-activated Ca^{2+} (CRAC) channels activate NFAT1 without the need for a rise in either bulk cytoplasmic or nuclear Ca^{2+} (Kar et al., 2011; Kar and Parekh, 2015). On the other hand, NFAT4 requires both CRAC channel-generated Ca^{2+} microdomains and a nuclear Ca^{2+} rise for sustained nuclear residency following stimulation with thapsigargin (Kar and Parekh, 2015), a widely used non-physiological agent that inhibits SERCA pumps and leads to store depletion and subsequent opening of CRAC channels. The nuclear Ca^{2+} increase that occurs upon stimulation with thapsigargin is a two-step process: Ca^{2+} first enters the cytoplasm through plasmalemmal CRAC channels and then diffuses into the nucleus, likely through nuclear pores. In this study, we asked what was the source of nuclear Ca^{2+} following stimulation with a physiological trigger. Unexpectedly, we find that the nuclear Ca^{2+} needed to activate NFAT4 is mobilized from the nuclear membrane itself. Our results reveal that two geographically distinct Ca^{2+} signals, one at the cell periphery and the other constrained within the nucleoplasm, are necessary and sufficient to drive NFAT4 activation and gene expression independent of the interspersed cytoplasmic Ca^{2+} signal.

 © 2016 The Author(s). Published by Elsevier Inc.
This is an open access article under the CC BY license (http://creativecommons.org/licenses/by/4.0/).

CellPress

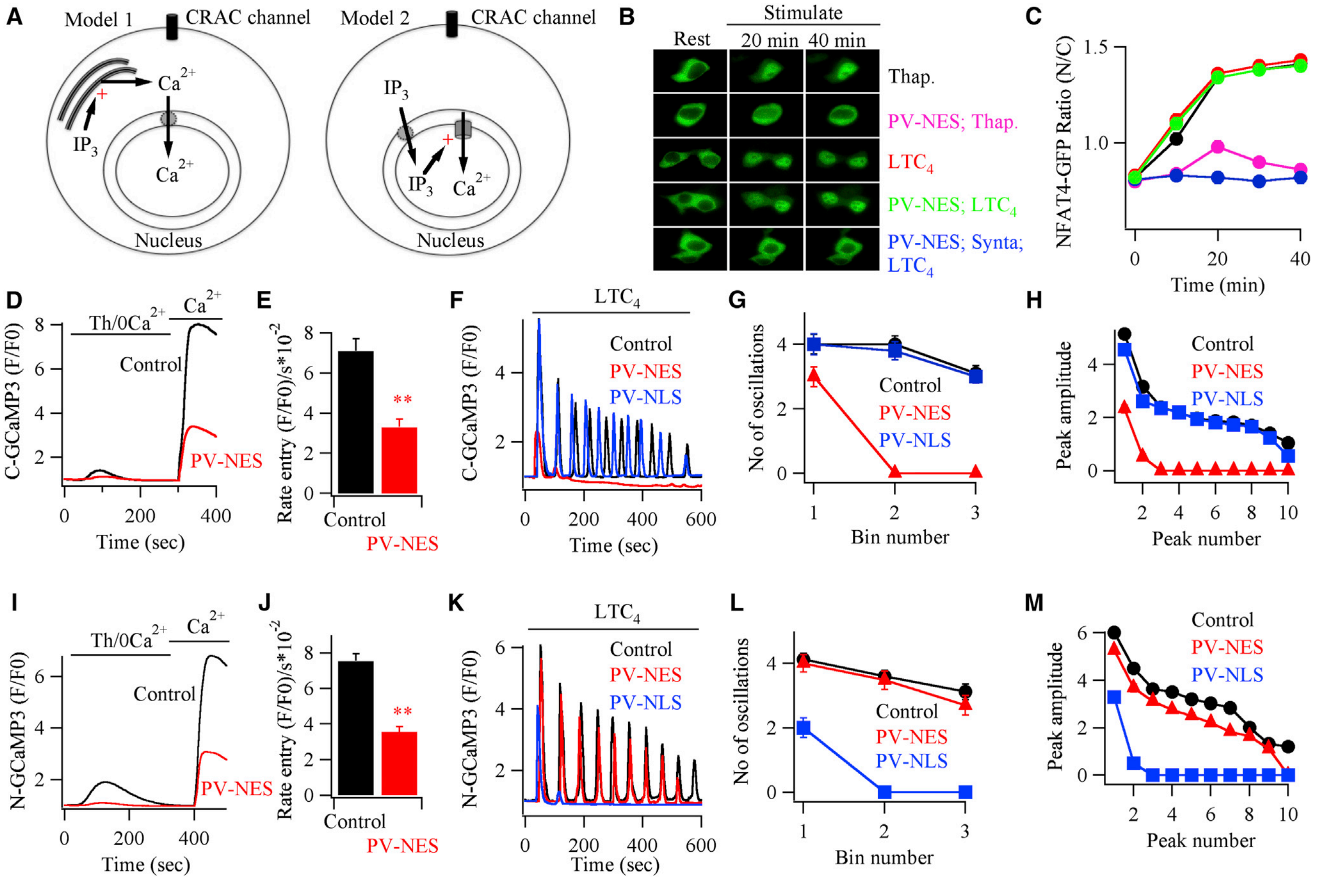

Figure 1. Buffering Cytoplasmic Ca^{2+} Does Not Impair Agonist-Evoked NFAT4 Nuclear Migration

(A) Cartoons summarize the two possible models for increasing nuclear Ca^{2+} following cysLT1 receptor activation (see text for details).

(B) Images show NFAT4-GFP nuclear migration for the different conditions indicated.

(C) Graphs summarize aggregate data from between 7 and 9 cells for each condition. Colors of graphs correspond to labels in (B). Time denotes time after stimulation.

(D) Cytoplasmic Ca^{2+} signals, measured with C-GCaMP3, following thapsigargin stimulation are shown for a control cell and one expressing PV-NES.

(E) Histogram compares rate of rise of the cytoplasmic Ca^{2+} signal following Ca^{2+} readdition to thapsigargin-treated cells, as in (D). Bars are averages of 19 (control) and 11 (PV-NES) cells.

(F) Cytoplasmic Ca^{2+} oscillations to 160 nM LTC_4 are shown for the conditions indicated. Untagged PV constructs were used here.

(G) Number of oscillations per 200 s bin after stimulation are compared. Each point is the mean of 10–21 cells.

(H) Peak amplitude of each oscillation is compared between the different conditions. Peak amplitude represents (F/F0) − 1.

(I–M) As in (D)–(H), but now nuclear Ca^{2+} was measured instead, using N-GCaMP3. In (J), control is mean from 22 cells and PV-NES from 19 cells. In (L), each point is the mean of between 11 and 26 cells. For most graphs, error bars are contained within the symbols. For the graphs and histograms, data are represented as mean ± SEM. See also Figures S1 and S2.

RESULTS

Physiological Stimulation Activates NFAT4 Independent of Bulk Cytoplasmic Ca^{2+}

Activation of cell-surface G protein-coupled cysteinyl leukotriene type I (cysLT1) receptors with the pro-inflammatory molecule leukotriene C_4 (LTC_4) evokes repetitive baseline cytoplasmic Ca^{2+} oscillations that are similar to those widely observed in other cells types and in response to different phospholipase C-coupled agonists (Thomas et al., 1996). In principal, these receptors can raise nuclear Ca^{2+} and thus maintain activation of NFAT4 in one of two ways: by an increase in bulk cytoplasmic Ca^{2+}, arising mainly from Ca^{2+} release through inositol 1,4,5-trisphosphate ($InsP_3$) receptors embedded in the endoplasmic reticulum (with a contribution from Ca^{2+} entry through CRAC channels), which then invades the nucleus (Model 1; Figure 1A), or by Ca^{2+} release directly into the nucleoplasm from nuclear $InsP_3$ receptor channels (Model 2; Figure 1A).

Model 1 predicts that an increase in cytoplasmic Ca^{2+} buffering should prevent NFAT4 activation by cysLT1 receptor stimulation because this manipulation would reduce the rate and extent of Ca^{2+} diffusion from the cytoplasm into the nucleus. By contrast, the expectation from Model 2 is that NFAT4 activation will be unaffected by changes in cytoplasmic Ca^{2+} buffering capacity. To test these predictions, we overexpressed the

cytoplasmic Ca^{2+} buffer parvalbumin that had been genetically manipulated to express only in the cytoplasm through addition of a nuclear export sequence (PV-NES) (Kar and Parekh, 2015). Although stimulation with thapsigargin led to robust migration of NFAT4-GFP from the cytoplasm into the nucleus in control cells, expression of PV-NES largely suppressed NFAT4 migration (Figures 1B and 1C) (Kar and Parekh, 2015). PV-NES therefore buffers cytoplasmic Ca^{2+} sufficiently well to prevent a rise in bulk cytoplasmic Ca^{2+} following CRAC channel opening from invading the nucleus to maintain active NFAT4. Activation of cysLT1 receptors with LTC_4 also resulted in robust nuclear accumulation of NFAT4 (Figures 1B and 1C). However, this was unaffected by the presence of PV-NES (Figures 1B and 1C). Ca^{2+} microdomains near open CRAC channels remained essential for NFAT4 activation by LTC_4 in the presence of PV-NES because channel block with Synta66 prevented nuclear NFAT4 migration (Figures 1B and 1C).

To confirm that PV-NES indeed reduced stimulus-evoked Ca^{2+} signals, we measured bulk cytoplasmic Ca^{2+} with the fluorescent Ca^{2+} indicator dye Fura-2. The Ca^{2+} response was significantly decreased by the presence of PV-NES following stimulation with thapsigargin (Figure S1). The intracellular Ca^{2+} signal measured with Fura-2 includes contributions from the cytoplasm and the nucleus. To measure Ca^{2+} in each compartment, we used the genetically encoded fluorescent indicator protein GCaMP3, engineered to express either a nuclear export sequence (GCaMP3-NES) or nuclear localization sequence (GCaMP3-NLS), to report Ca^{2+} in the cytoplasm or nucleus, respectively. In control cells expressing GCaMP3-NES, thapsigargin activated a robust Ca^{2+} signal due to Ca^{2+} influx, but the response was reduced by $\sim$60% following expression of PV-NES (Figures 1D and 1E). Similarly, cytoplasmic Ca^{2+} oscillations to LTC_4 ran down more quickly (Figures 1F and 1G) and were smaller in size (Figures 1F and 1H) in the presence of PV-NES. The nuclear Ca^{2+} rise induced by thapsigargin (measured with GCaMP3-NLS) was also substantially reduced by PV-NES (Figures 1I and 1J), as expected since this increase is secondary to the cytoplasmic Ca^{2+} rise.

LTC_4 evoked numerous nuclear Ca^{2+} oscillations in control cells, and these were largely unaffected by the presence of PV-NES (Figures 1K–1M), as was the case for NFAT4 migration (Figures 1B and 1C). When parvalbumin was targeted to the nucleus instead (PV-NLS), NFAT4 migration in response to LTC_4 challenge was suppressed (Figure S2) (Kar and Parekh, 2015), as were the nuclear Ca^{2+} oscillations (Figures 1K–1M). The cytoplasmic Ca^{2+} oscillations evoked by LTC_4 were unaffected by the presence of PV-NLS (Figures 1F–1H).

Equivalent results were obtained when we overexpressed a different cytoplasmic Ca^{2+}-binding protein, calbindin D_{28K}-GFP: NFAT4-cherry failed to accumulate in the nucleus after stimulation with thapsigargin (middle set of images in Figure 2A; Figure 2B). By contrast, activation of cysLT1 receptors triggered robust migration of NFAT4-cherry into the nucleus in cells expressing calbindin D_{28K}-GFP, which was prevented by Synta66 (Figures 2A and 2B).

NFAT1 activation, which is driven only by local Ca^{2+} entry through CRAC channels, was unaffected by the presence of PV-NES (Kar and Parekh, 2015) or calbindin D_{28K}-GFP (upper set of images in Figure 2A; Figure 2B), regardless of whether the stimulus was thapsigargin or LTC_4.

Collectively, these data show that cysLT1 receptor stimulation activates NFAT4 even when a rise in bulk cytoplasmic Ca^{2+} has been impaired. The findings are therefore incompatible with the various scenarios embodied in Model 1 (Figure 1), where nuclear Ca^{2+} is totally contingent upon a cytoplasmic Ca^{2+} rise. Instead, our data are consistent with Model 2, where the nuclear Ca^{2+} increase required for maintained NFAT4 activation is independent of cytoplasmic Ca^{2+}. Model 2 posits that nuclear Ca^{2+} is increased by activation of nuclear $InsP_3$ receptors and is therefore causally attendant to a rise in nucleoplasmic $InsP_3$. The nuclear envelope is a functional Ca^{2+} store due to the presence of SERCA pumps on the outer membrane and $InsP_3$ receptors on both the inner and outer membranes (Alonso and García-Sancho, 2011; Gerasimenko et al., 1996), with $InsP_3$ releasing stored Ca^{2+} directly into the nucleoplasm (Echevarría et al., 2003; Humbert et al., 1996; Zima et al., 2007). Although LTC_4 triggered NFAT4 accumulation in the nucleus in PV-NES- and calbindin-expressing cells, it failed to do so if cells were pre-exposed to thapsigargin (Figures 2C and 2D). This supports Model 2 in that sustained activation of NFAT4 by receptor stimulation requires a functional nuclear Ca^{2+} store.

Buffering Nuclear $InsP_3$ Prevents the Nuclear Ca^{2+} Rise to Agonist and Subsequent Sustained NFAT4 Activation

We designed experiments to test Model 2 more directly. A key prediction is that buffering nuclear $InsP_3$ should impair $InsP_3$ from activating $InsP_3$ receptors in the inner nuclear membrane, preventing the rise in nuclear Ca^{2+} and thus sustained nuclear NFAT4 activation. To address this, we dampened $InsP_3$ increases in either the cytoplasm or the nucleus by exploiting genetically encoded $InsP_3$ buffers. We expressed a construct containing the $InsP_3$ binding site of the $InsP_3$ receptor tagged with RFP and engineered to express either a cytoplasmic or nuclear localization sequence, which we refer to as IP_3 buffer-NES-RFP and IP_3 buffer-NLS-RFP, respectively. Expression of IP_3 buffer-NES-RFP was localized to the cytoplasm (Figures 3A and 3B), whereas IP_3 buffer-NLS-RFP was restricted to the nucleus (Figures 3A and 3B). Coexpression of both buffers resulted in a similar distribution between nucleus and cytoplasm. Stimulation with thapsigargin, which evokes Ca^{2+} signals independent of $InsP_3$, resulted in robust nuclear accumulation of NFAT1-GFP or NFAT4-GFP despite the presence of IP_3 buffer-NES-RFP, IP_3 buffer-NLS-RFP, or both (Figure 3C; aggregate data are summarized in Figure 3K). Therefore these buffers per se do not interfere with NFAT migration.

After expression of $InsP_3$ buffer-NES-RFP, cytoplasmic (Figures 3D–3F) and nuclear (Figures 3G–3I) Ca^{2+} signals to cysLT1 receptor activation were significantly reduced. Activation of either NFAT1 or NFAT4 was also suppressed by $InsP_3$ buffer-NES-RFP (Figures 3J and 3K), as expected since cytoplasmic $InsP_3$ is required to deplete the endoplasmic reticulum Ca^{2+} store for CRAC channel activation following cysLT1 receptor stimulation. Movement could be rescued by a dose of ionomycin that raises bulk cytoplasmic Ca^{2+} to high levels independent of store-operated Ca^{2+} influx (Figures 3J and 3K; Kar and Parekh 2015). Markedly different results were obtained when $InsP_3$

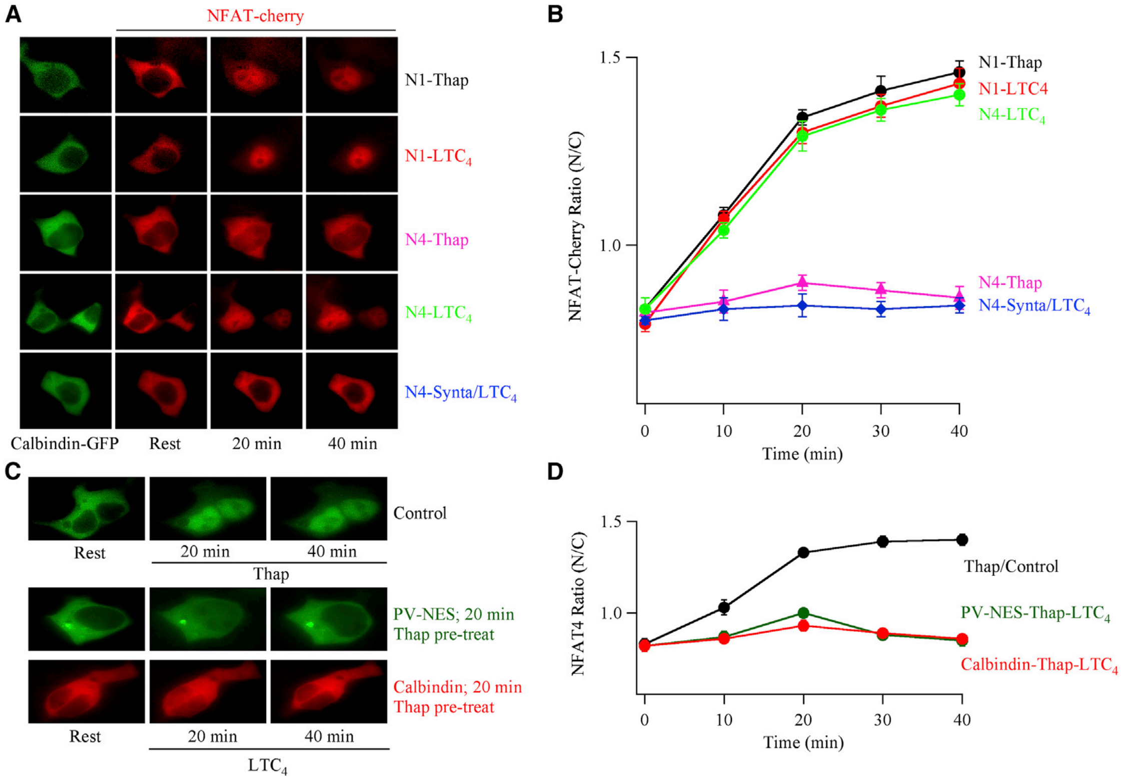

Figure 2. The Cytoplasmic Ca^{2+} Binding Protein Calbindin D_{28K} Prevents Nuclear Migration of NFAT4, but Not NFAT1

(A) Images compare NFAT1- or NFAT4-cherry movement in cells expressing calbindin D_{28K}-GFP and were taken at different times after stimulation, as indicated. NFAT constructs used are shown on the right of each row of images, where N1 and N4 denote NFAT1 and NFAT4. Stimuli are also shown on the right.

(B) Aggregate data from experiments as in (A) are summarized. Each point is the mean of between 8 and 13 individual cells. The N1-Thap curve has been offset upward by 0.03, to increase clarity.

(C) In cells expressing PV-NES (untagged) or calbindin D_{28K}-GFP (indicated on the right of each image), stimulation with LTC_4 after thapsigargin (1 μM) failed to show any NFAT4 movement. LTC_4 was applied 20 min after pre-treatment with thapsigargin. Upper images show rest and then 20 and 40 min after thapsigargin stimulation (control recording from the same cell preparation). In the control and PV-NES experiments, NFAT4-GFP was expressed. In coexpression with calbindin D_{28K}-GFP, NFAT4-cherry was used instead.

(D) Aggregate data are compared for the conditions shown. Each point is the mean of between 9 and 15 cells. For the graphs, data are represented as mean ± SEM.

buffer-NLS-RFP was expressed instead. Cytoplasmic Ca^{2+} oscillations to LTC_4 were now largely unaffected (Figures 3D–3F), whereas the nuclear Ca^{2+} signals were suppressed (Figures 3G–3I). Despite cytoplasmic Ca^{2+} signals being normal in the presence of IP_3 buffer-NLS, activation of cysLT1 receptors now failed to stimulate NFAT4 accumulation in the nucleus (Figures 3J and 3K). Movement could subsequently be rescued by ionomycin. Simultaneous measurements of cytoplasmic and nuclear Ca^{2+} in the same cells confirmed that Ca^{2+} oscillated in both compartments after stimulation with LTC_4, but only the nuclear Ca^{2+} oscillations were abolished by $InsP_3$ buffer-NLS (Figure S3). Oscillations in cytoplasmic and nuclear Ca^{2+} in both compartments were suppressed by coexpression of $InsP_3$ buffer-NES and $InsP_3$ buffer-NLS (Figure S3). Migration of NFAT1 into the nucleus in response to LTC_4 was unaffected by $InsP_3$ buffer-NLS-RFP (Figures 3J and 3K), consistent with it requiring only local Ca^{2+} entry through CRAC channels for activation. Nuclear migration of either NFAT1 or NFAT4 was suppressed by coexpression of both $InsP_3$ buffers (Figures 3J and 3K). In aggregate, these data suggest that the nuclear Ca^{2+} rise required for NFAT4 residency within the nucleus is accomplished through Ca^{2+} release by nuclear $InsP_3$. In support of this, immunostaining revealed that type I $InsP_3$ receptors localized to the nucleus as well as the cytoplasm (Figure 4A). $InsP_3$ receptors were also detected in both nuclear and cytoplasmic extract (Figure 4B).

In order for $InsP_3$ buffer-NLS to suppress nuclear Ca^{2+} oscillations, leaving cytoplasmic ones intact, $InsP_3$ receptors must be embedded within the inner nuclear membrane facing the nucleoplasm. To assess this, we used electron microscopy with immunogold labeling of type I $InsP_3$ receptors, an approach that provides the spatial resolution to resolve the specific nuclear location of proteins. Whereas no detectable immunogold labeling was seen in control cells not exposed to $InsP_3$ receptor antibody (Figure 4C, left-hand panel, labeled immunogold−), the inner nuclear membrane revealed particles when $InsP_3$ antibody was present (Figure 4C, right-hand panel, labeled immunogold+).

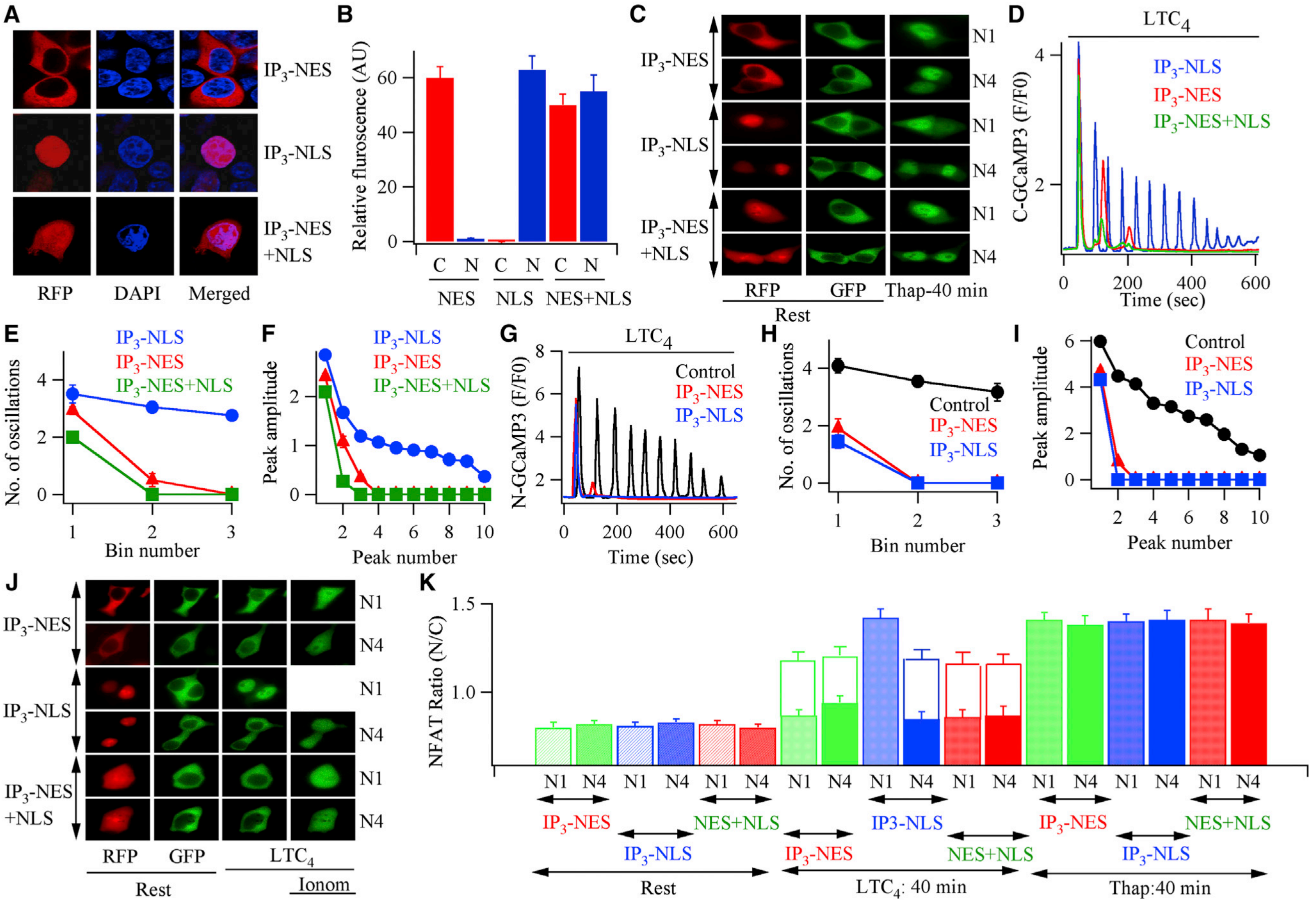

Figure 3. Buffering Nuclear InsP$_3$ Prevents Nuclear Accumulation of NFAT4

(A) Confocal images compare sub-cellular distribution of RFP-tagged InsP$_3$ buffer containing either a nuclear export sequence (IP$_3$-NES; upper panel), InsP$_3$ buffer containing a nuclear localization sequence (IP$_3$-NLS, middle panel), or both (lower panel). DAPI was used to stain the nucleus.

(B) Fluorescence intensities of the InsP$_3$ buffers are compared between the cytoplasmic and nuclear compartments, denoted C and N, respectively. Each bar is the mean of between 9 and 15 cells.

(C) Migration of NFAT1-GFP (denoted N1) and NFAT4-GFP (N4) into the nucleus is compared following stimulation with thapsigargin for 40 min in the presence of the different IP$_3$ buffers (RFP-tagged), indicated on the left.

(D) Cytoplasmic Ca^{2+} oscillations are compared between cells expressing RFP-tagged IP$_3$-NLS, IP$_3$-NES, or both.

(E) The number of oscillations to LTC$_4$ produced each 200 s bin are compared for the different conditions. Each point is the mean of between 8 and 11 cells.

(F) The amplitude of each Ca^{2+} oscillation is compared for the conditions shown. Data for (E) and (F) were extracted from experiments as in (D).

(G) Nuclear Ca^{2+} oscillations are compared for the different conditions indicated.

(H) The number of nuclear Ca^{2+} oscillations per 200 s bin is shown for each condition. Each point is the mean of between 10 and 26 cells.

(I) The peak amplitude of each nuclear Ca^{2+} oscillation is compared. Data for (H) and (I) were extracted from experiments as in (G). In (G)–(I), control denotes mock-transfected cells.

(J) Images compare movement of NFAT1-GFP or NFAT4-GFP for the conditions shown. IP$_3$ buffers were tagged with RFP. LTC$_4$ was applied at 160 nM for 40 min and ionomycin (ionom) was 2 μM.

(K) Histogram summarizes data for the various conditions shown. Rest denotes the unstimulated state. N1 and N4 indicate NFAT1-GFP and NFAT4-GFP. Open bars above filled histograms denote the rescue of NFAT movement following stimulation with ionomycin for 20 min. Each bar is the mean from three independent experiments.

In (B), (E), (F), (H), (I), and (K), data are represented as mean ± SEM. See also Figure S3.

In some studies, the nuclear Ca^{2+} rise is secondary to the cytoplasmic Ca^{2+} increase, which rapidly propagates into the nucleus (Lin et al., 1994; Lipp et al., 1997). On the other hand, nuclear Ca^{2+} can increase independently of cytoplasmic Ca^{2+} (Echevarría et al., 2003; Humbert et al., 1996; Zima et al., 2007). In our experiments with cysLT1 receptor activation, cytoplasmic Ca^{2+} oscillations did not invade the nucleoplasm to the extent needed to maintain NFAT4 residency because InsP$_3$ buffer-NLS-RFP greatly reduced the nuclear Ca^{2+} signals (Figures 3G–3I) and suppressed transcription factor accumulation (Figure 3K), despite cytoplasmic Ca^{2+} signaling remaining intact (Figures 3D–3F; Figure S3). Furthermore, buffering cytoplasmic Ca^{2+} with parvalbumin-NES abolished cytoplasmic calcium oscillations to LTC$_4$ (Figures 1F–1H), yet nuclear Ca^{2+} signaling

CellPress

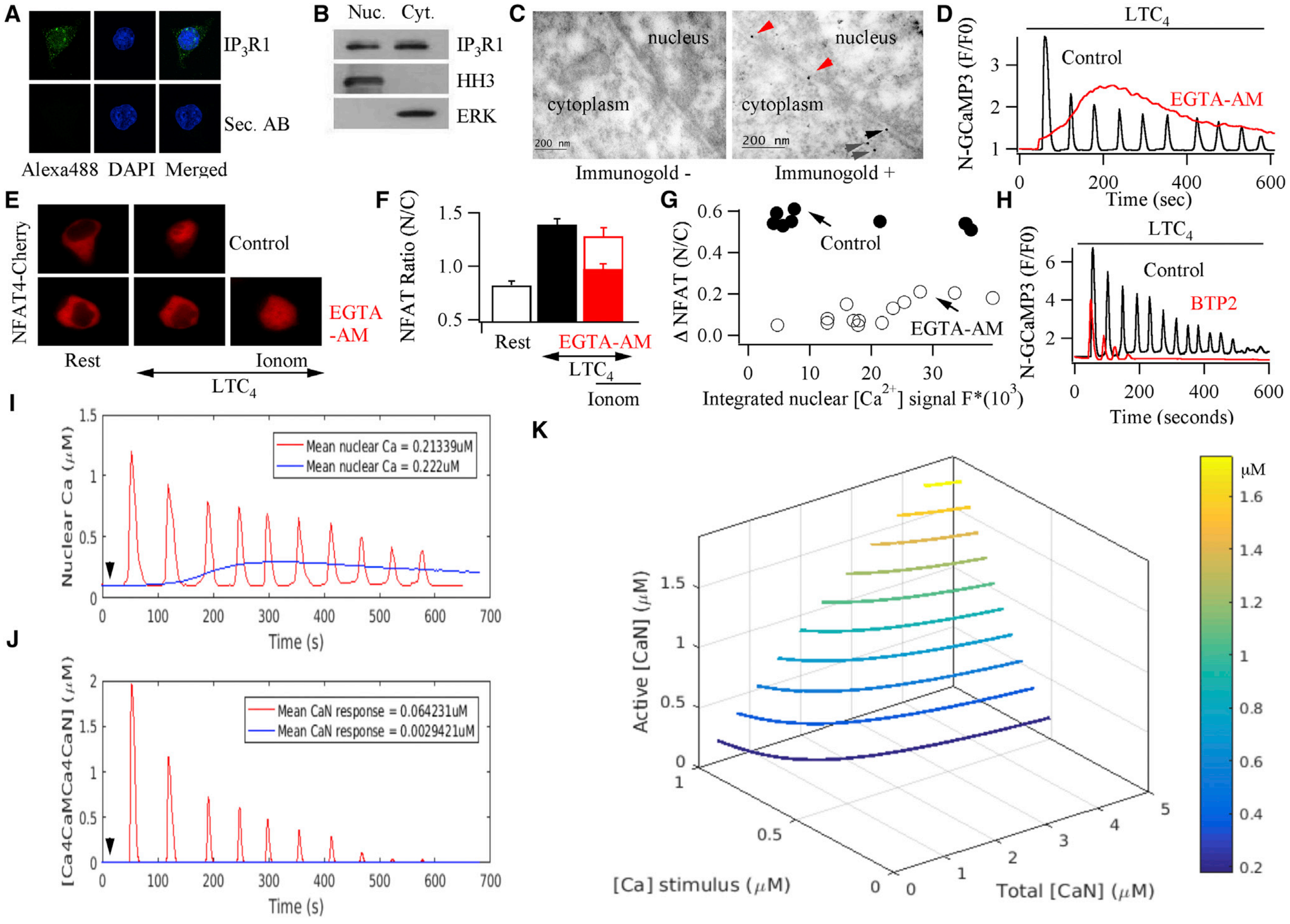

Figure 4. Nuclear Ca^{2+} Oscillations Are Generated by Nuclear $InsP_3$ Receptors and Are Effective in Maintaining NFAT4 Activity

(A) Immunostaining reveals the presence of type I $InsP_3$ receptors in the cytoplasm and nucleus. No staining is seen when secondary antibody (Sec. AB) alone is used. Secondary antibody was conjugated with Alexa 488 for visualization.

(B) Western blots of cytoplasmic and nuclear extract show the presence of type I $InsP_3$ receptors in both compartments. Similar results were obtained in two independent experiments.

(C) Immunogold labeling reveals the presence of type I $InsP_3$ receptors in the inner nuclear membrane. Left-hand panel denotes a control micrograph and the right-hand one after immunogold labeling of type I $InsP_3$ receptors. Red arrows denote $InsP_3R$ on the inner nuclear membrane, black arrow shows receptor on the outer nuclear membrane, and gray ones denote receptor in the endoplasmic reticulum.

(D) Comparison of nuclear Ca^{2+} signals to LTC_4 in a control cell and in one in which the cytoplasm and nucleoplasm had been loaded with EGTA via the form EGTA-AM.

(E) Loading the cell with EGTA reduces NFAT4-cherry nuclear migration in response to LTC_4. Migration can be rescued by stimulating cells with ionomycin (2 μM; 20 min).

(F) Aggregate data from several experiments as in (E) are summarized. Open bar above EGTA-AM denotes the extent of rescue by ionomycin. Data are represented as mean ± SEM.

(G) Graph plots NFAT4-cherry nuclear accumulation against the integrated nuclear Ca^{2+} signal in control cells and cells loaded with EGTA. Each point depicts NFAT4 movement and integrated nuclear Ca^{2+} from the same cell. The y axis represents peak NFAT nuclear/cytoplasmic ratio after stimulation with LTC_4 minus the NFAT nuclear/cytoplasmic ratio before stimulation.

(H) Nuclear Ca^{2+} oscillations evoked by LTC_4 run down quickly when Ca^{2+} entry through CRAC channels is blocked with 10 μM BTP2.

(I) Estimated nuclear Ca^{2+} concentration for an oscillatory response in a control cell is compared with a plateau-type response in an EGTA-loaded cell, following stimulation with 160 nM LTC_4. To calibrate the nuclear Ca^{2+} signal in intact cells, following expression of NGCaMP3, Rmin and Rmax were obtained by stimulating cells with 5 μM ionomycin either in Ca^{2+}-free external solution (with fluorescence measured ~15 min later) or 10 mM Ca^{2+} external solution, respectively. The K_D for nuclear GCaMP3 was taken as 660 nM from the literature. The estimated Ca^{2+} concentrations are, at best, only an approximation (see text). Arrow denotes LTC_4 application.

(J) The graph plots the predicted build-up of nuclear Ca^{2+}-calmodulin-calcineurin following stimulation with LTC_4 in a control cell (red trace) and in one loaded with EGTA (blue trace).

(K) Contour plot depicts active nuclear calcineurin as a function of nuclear Ca^{2+} concentration and total nuclear calcineurin available.

See also Figure S4, Table S1, and description of the model in Supplemental information.

(Figures 1K–1M) and NFAT4 activation (Figure 1C) were unaffected. We also considered the possibility that $InsP_3$ released Ca^{2+} into the cytoplasm from receptors located in the outer nuclear membrane and Ca^{2+} then diffused into the nucleus through nuclear pores. Because PV-NES had no inhibitory effect on nuclear Ca^{2+} signals evoked by agonist, this possibility is unlikely.

How does cysLT1 receptor activation increase nuclear Ca^{2+} independently of cytoplasmic Ca^{2+}? Our finding that $InsP_3$ buffer-NES-RFP suppressed the nuclear Ca^{2+} rise to LTC_4 demonstrates that $InsP_3$ is first produced in the cytoplasm and then diffuses into the nucleus, rather than local generation of $InsP_3$ within the nucleus itself. The lifetime of $InsP_3$ in the cytoplasm is sufficiently long for it to diffuse many times across a cell with dimensions similar to that of a HEK cell before it is broken down (Allbritton et al., 1992).

For the Same Integrated Nuclear Ca^{2+} Signal, Nuclear Ca^{2+} Oscillations Are More Effective in Maintaining NFAT4 Activation than a Bulk Nuclear Ca^{2+} Rise

We asked whether nuclear Ca^{2+} oscillations conferred a signaling advantage to sustained NFAT4 activation compared with a non-oscillatory Ca^{2+} increase that raised nucleoplasmic Ca^{2+} to a similar extent over the same stimulation period (integrated Ca^{2+} signal). Simultaneous measurements of nuclear Ca^{2+} using GCaMP3-NLS and nuclear accumulation of NFAT4-cherry showed that oscillatory nuclear Ca^{2+} signals induced by LTC_4 (Figure 4D) evoked robust nuclear accumulation of NFAT4 (Figures 4E and 4F) for only a small integrated nuclear Ca^{2+} signal (Figure 4G; labeled control). In cells loaded with the slow Ca^{2+} chelator EGTA, which increases the buffering capacity of the nucleoplasm (Figure S3 in Kar and Parekh, 2015), nuclear Ca^{2+} oscillations to LTC_4 were replaced by a steady increase in nuclear Ca^{2+} (Figure 4D). Integration of these signals showed similar or greater total nuclear Ca^{2+} over the same time period compared with oscillatory responses (Figure 4G), yet NFAT4 accumulation was substantially less (Figures 4E–4G).

The Nuclear Ca^{2+} Store Is Replenished by Store-Operated Ca^{2+} Entry

Ca^{2+} entry through CRAC channels is required to refill the endoplasmic reticulum with Ca^{2+}. Since the endoplasmic reticulum is contiguous with the outer nuclear membrane, we asked whether Ca^{2+} entry through CRAC channels was likewise essential for repletion of the nuclear store. Whereas numerous nuclear Ca^{2+} oscillations were seen in control cells stimulated with LTC_4, the Ca^{2+} signals ran down quickly in the presence of the CRAC channel blocker BTP2 (Figure 4H; Figure S4). Similar results were obtained when cells were stimulated either in the presence of another CRAC channel blocker (Synta66) or in the absence of external Ca^{2+} (data not shown).

Ca^{2+} influx contributes little to the size or decay rate of each cytoplasmic Ca^{2+} oscillation evoked by LTC_4 (Di Capite et al., 2009). The finding that the nuclear store is replenished by Ca^{2+} flux through CRAC channels at the cell surface is therefore a remarkable example of Ca^{2+} tunneling (Mogami et al., 1997), where local Ca^{2+} entry can be transported to distant cytoplasmic regions via uptake into the endoplasmic reticulum.

Mathematical Modeling of Nuclear Calcineurin Activation

We simulated active calcineurin build-up in the nucleus (Figures 4I–4K) for different patterns of nuclear Ca^{2+} signal, using a reduction of the Saucerman and Bers model (Saucerman and Bers, 2008), as suggested by Bazzazi et al. (Bazzazi et al., 2015; see Supplemental Information for further details). To model active calcineurin levels, an estimate of nuclear Ca^{2+} concentration was needed. We therefore attempted to calibrate the GCaMP3 signal, as described in the legend to Figure 4I. The concentrations shown in Figure 4I are, however, only a rough estimate because of the inherent difficulties in calibrating a fluorescent probe within the nucleoplasm of an intact cell (Bootman et al., 2009), coupled with GCaMP3 being a non-ratiometric probe. Notwithstanding these limitations, the model suggests calcineurin activity oscillates in the nucleus (Figure 4J) with a pattern similar to the nuclear Ca^{2+} oscillations (Figure 4I). By contrast, nuclear calcineurin activity is very low when a small-amplitude but long-lasting nuclear Ca^{2+} rise occurs. A contour plot comparing active calcineurin with Ca^{2+} concentration and total nuclear calcineurin (Figure 4K) revealed that the same level of active calcineurin could be achieved by having either high total nuclear calcineurin combined with low nuclear Ca^{2+} or low total nuclear calcineurin coupled with high nuclear Ca^{2+}. The contour plot also shows that large nuclear Ca^{2+} spikes enable robust activation of a small number of nuclear calcineurin molecules, whereas more sustained smaller Ca^{2+} rises are much less effective. This suggests an advantage to large oscillatory Ca^{2+} signaling: in an individual cell having a fixed total number of effector molecules like calcineurin, a small number of effectors will be activated strongly by large-amplitude Ca^{2+} oscillations, compared with weak recruitment of a greater number of effectors in response to smaller-amplitude but more prolonged Ca^{2+} signals. For an effector that is an enzyme like calcineurin, a small fraction of active molecules should be sufficient to engender a strong downstream response.

NFAT4 Is Rephosphorylated within the Nucleus Much Faster than NFAT1

NFAT4 is exported out of the nucleus ~8 times faster than NFAT1 (Kar and Parekh, 2015; Yissachar et al., 2013), an effect due partly to differences in the respective SP-3 motifs (Kar and Parekh, 2015). The nuclear export of each isoform is a two-step process involving rephosphorylation within the nucleoplasm followed by exit through the nuclear pores in a complex with shuttle proteins CRM1 and Ran-GTP. The relative contributions of these steps are unknown. We hypothesized that the main difference in NFAT nuclear export rates was due to differences in nuclear rephosphorylation rather than nuclear transport, and this was why nuclear Ca^{2+} and thus active calcineurin was so important for NFAT4 accumulation. To address this, we exposed cells to leptomycin B, an effective inhibitor of CRM1-dependent export (Fornerod et al., 1997). In the presence of leptomycin B, NFAT4 accumulated within the nucleus following CRAC channel activation at a similar rate to control cells (Figures 5A and 5B). Following nuclear accumulation, we triggered nuclear export by termination of Ca^{2+} influx combined with inhibition of calcineurin with cyclosporine A (Kar et al., 2011). Compared with

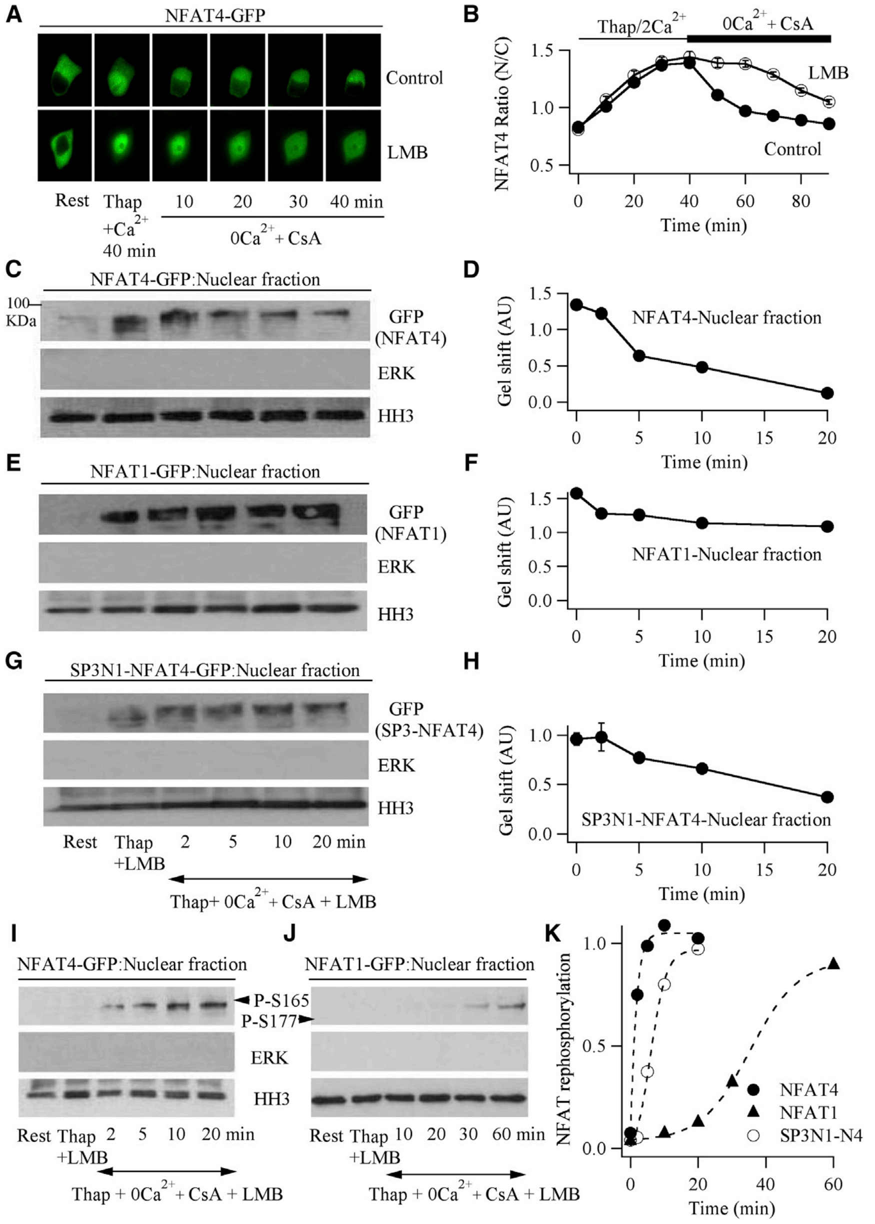

Figure 5. NFAT4 Is Rephosphorylated within the Nucleus Considerably More Quickly Than NFAT1

(A) Images compare nuclear export of NFAT4-GFP between a control cell and one exposed to leptomycin B (LMB; 100 nM). Nuclear export was initiated by removal of external Ca^{2+} in the presence of cyclosporine A.

(B) Aggregate data from several experiments are compared.

(C) Western blot shows gel shift of NFAT4-GFP from nuclear extract after stimulation and then at different times (2–20 min) after initiation of export (see bottom of G for timings).

(D) Graph plots relative gel shift as a function of time up to 20 min after the initiation of nuclear export. A decreased gel shift denotes rephosphorylation.

(E) Gel shift for nuclear NFAT1-GFP under conditions identical to (C).

(F) Graph shows relative gel shift for NFAT1-GFP, as in (D).

(G) Gel shift for the SP3NFAT1-NFAT4-GFP construct.

(H) Graph plots relative gel shift for SP3NFAT1-NFAT4-GFP, as in (D). For the gels in (C), (E), and (G), conditions for each lane are shown only in (G). For the resting condition (indicated as Rest), no detectable NFAT was seen in any of the gels because the gels show nuclear extract and, at rest, there is very little NFAT in the nucleus.

(I) Blot compares kinetics of phosphorylation of serine 165 in NFAT4 from the nuclear fraction, denoted as P-S165.

(J) As in (I), but phosphorylation of serine 177 (P-S177) in NFAT1 was measured.

(K) Graphs plot the kinetics of phosphorylation of serine 165 NFAT4 and SP3N1-N4 or serine 177 in NFAT1.

In (C), (E), (G), (I), and (J), HH3 (histone H3) was used as a nuclear marker and ERK was taken as a cytoplasmic marker. Rest denotes the non-stimulated state. Thap + LMB represents the gel shift seen 40 min after stimulation with thapsigargin in the presence of LMB and external Ca^{2+}, when NFAT had fully accumulated in the nucleus. Nuclear extract was isolated at the different times indicated. In all panels, 100 nM leptomycin B was added 10 min before thapsigargin and then maintained throughout. In the graphs, data are represented as mean ± SEM. See also Figure S5.

control cells, where NFAT4 exited the nucleus with a time constant of ~10 min (Kar and Parekh, 2015), nuclear export was suppressed for ~20 min in leptomycin B-treated cells (Figures 5A and 5B). Thereafter, NFAT4 export proceeded gradually, reflecting either delayed leptomycin B-insensitive transport or loss of leptomycin B activity over time. Regardless, the 20 min hiatus in NFAT4 export in leptomycin B enabled us to measure the kinetics of NFAT4 rephosphorylation within the nucleus in the absence of export. Gel shifts from western blots of nuclear extract revealed that NFAT4 was rephosphorylated quickly (Figure 5C), with a half-time of ~4.5 min (Figure 5D). By contrast, only modest nuclear phosphorylation of NFAT1 occurred within 20 min (Figures 5E and 5F). An NFAT4 construct in which the SP-3 region of NFAT4 had been replaced with that from NFAT1 was rephosphorylated more slowly than NFAT4 but faster than NFAT1 (Figures 5G and 5H; half-time of ~15 min), supporting the view that the SP-3 region is an important determinant of nuclear NFAT rephosphorylation (Kar and Parekh, 2015). For the 20 min that NFAT-GFP export was suppressed (Figure 5B), no increase in cytoplasmic NFAT-GFP was detectable (Figures S5A–S5C). Hence, over this time frame, leptomycin B fully inhibits NFAT nuclear export.

In the Ar-5 T cell clone, significant rephosphorylation of NFAT1 occurs within 15 min (Loh et al., 1996), which is considerably

faster than the rate we find in HEK293 cells (Figure 5F). In RBL-2H3 cells, NFAT1 was exported out of the nucleus with a rate constant of ~0.06 min^{-1} (Yissachar et al., 2013), which is ~4-fold faster than the rate constant (~0.014 min^{-1}) for the same isoform in HEK293 cells. Hence, the kinetics of NFAT1 export varies between different cell types, perhaps reflecting the activities of nuclear kinases.

Although gel-shift analysis is widely used to measure NFAT phosphorylation status, shifts can be induced by other post-translational modifications. Additionally, since multiple serines are phosphorylated and then dephosphorylated (Okamura et al., 2000), the shift is a smear rather than a discrete change, which renders quantification somewhat selective. Because we analyzed the shifts for NFAT1 and NFAT4 in the same way, the relative difference in dephosphorylation/rephosphorylation kinetics seems valid. Nevertheless, we utilized an alternative approach to compare the kinetics of NFAT4 rephosphorylation/dephosphorylation with that of NFAT1. Serine 165 on NFAT4 is close to serine 177 on NFAT1; both are phosphorylated at rest and dephosphorylated by calcineurin, and commercial antibodies to these phosphorylated residues are available. We therefore tracked the phosphorylated state of each NFAT isoform using phospho-specific antibodies. At rest, both isoforms were phosphorylated and restricted to the cytoplasmic fraction (Figures S5D and S5E), with no detectable phosphorylated form in the nucleus (Figures 5I and 5J). Stimulation with thapsigargin for 30 min in the presence of leptomycin B led to a marked decrease in phosphorylation of both NFAT isoforms in cytoplasmic extract (Figures S5D and S5E), with no detectable change in the phosphorylated nuclear levels (Figures 5I and 5J). This is because only dephosphorylated NFAT migrates into the nucleus, and we are tracking phosphorylated residues. However, subsequent removal of external Ca^{2+} coupled with addition of cyclosporine A in the continued presence of leptomycin B led to rapid rephosphorylation of nuclear NFAT4 (Figure 5I) with a half-time of ~1.1 min (Figure 5K). Serine 177 on NFAT1, by contrast, was phosphorylated considerably more slowly (Figures 5J and 5K), with a half-time of ~35 min. In the SP3N1-NFAT4 chimera, serine 165 was rephosphorylated more slowly than NFAT4 but faster than NFAT1 (Figure 5K and Figures S5F and S5G; half-time of ~6.4 min), consistent with the gel-shift data in Figure 5H.

It is important to note that these phosphorylation studies detect only one phosphorylated residue in each NFAT isoform and do not represent the kinetics of total rephosphorylation. It is possible that serine 165 is the first residue in NFAT4 to be rephosphorylated, whereas serine 177 in NFAT1 is among the last, which could explain the different kinetics. The fact that serine 165 in the SP3N1-NFAT4 chimera was phosphorylated ~6-fold more slowly than in NFAT4, which is broadly similar to the 3.5-fold slower kinetics seen in the gel-shift assay, suggests NFAT4 is indeed phosphorylated more quickly than NFAT1 within the nucleus.

Differences in Cytoplasmic Rephosphorylation Rates of NFAT Isoforms

Taking advantage of leptomcyin B, our new data in Figure 5 have enabled us to quantify the kinetics of NFAT rephosphorylation within the nucleus (k6 reaction in Figure 6A). We therefore designed experiments to measure NFAT rephosphorylation kinetics in the cytoplasm (k_2 reaction in Figure 6A). Acetate inhibits NFAT migration into the nucleus by preventing association of the NFAT-importin β complex with tubulin α (Ishiguro et al., 2011). NFAT dephosphorylation proceeded normally, but the dephosphorylated protein was trapped within the cytoplasm (Ishiguro et al., 2011). We reasoned that this approach might enable us to measure the kinetics of NFAT rephosphorylation within the cytoplasm, independent of any nuclear flux. We first confirmed that acetate inhibited translocation of NFAT1 and NFAT4 into the nucleus (Figures 6B and 6C). We then activated CRAC channels with thapsigargin in the presence of acetate to drive NFAT dephosphorylation. After 20 min, a time sufficient for NFAT dephosphorylation (Kar et al., 2011), we rapidly inhibited calcineurin activity by terminating Ca^{2+} influx in the presence of cyclosporine A. This enabled the measure of NFAT rephosphorylation within the cytoplasm (k_2). NFAT4 was rephosphorylated quickly, with a half-time of ~4 min (Figures 6D and 6E). By contrast, NFAT1 was rephosphorylated considerably more slowly, with only ~40% recovery by 20 min (Figures 6D and 6E).

Similar results were seen with the phospho-serine antibodies against NFAT1 and NFAT4. NFAT4 was rephosphorylated significantly more quickly than NFAT1 (Figures 6F and 6G), consistent with the gel-shift data (Figure 6E).

NFAT1 but Not NFAT4 Nuclear Accumulation Shows Paired-Pulse Facilitation

A consequence of the faster k_2 reaction for NFAT4 than NFAT1 is that NFAT4 will be rephosphorylated in the cytoplasm considerably more quickly following deactivation of calcineurin. NFAT proteins are extensively phosphorylated by protein kinases (Okamura et al., 2000). A slower k_2 for NFAT1 means that, following a brief pulse of Ca^{2+} that is insufficient for complete NFAT1 dephosphorylation, the rephosphorylation process would proceed slowly. Hence, a second identical Ca^{2+} pulse a few minutes later should result in further net dephosphorylation and thus complete activation of NFAT1, imparting a form of paired-pulse facilitation to NFAT1 nuclear migration. Our previous experiments demonstrated robust paired-pulse facilitation to NFAT1 (Kar et al., 2012), although the underlying molecular basis was unclear. On the other hand, the faster rate of rephosphorylation of NFAT4 would result in more extensive rephosphorylation during the interpulse interval, and this would not result in additional dephosphorylation when a second identical Ca^{2+} pulse is applied, preventing paired-pulse facilitation. To test this, we measured NFAT4 nuclear dynamics following brief single or paired pulses of LTC_4. Activation of cysLT1 receptors to a single 2 min pulse failed to induce any detectable NFAT1 or NFAT4 nuclear migration over a subsequent 38 min period (Kar et al., 2012; Figure 6H). However, two identical LTC_4 pulses, applied 8 min apart, resulted in prominent NFAT1 but not NFAT4 nuclear accumulation 28 min later (38 min after the first pulse; Figure 6H). Similar paired-pulse facilitation was obtained when two identical Ca^{2+} pulses were applied to thapsigargin-treated cells instead (Figure S6).

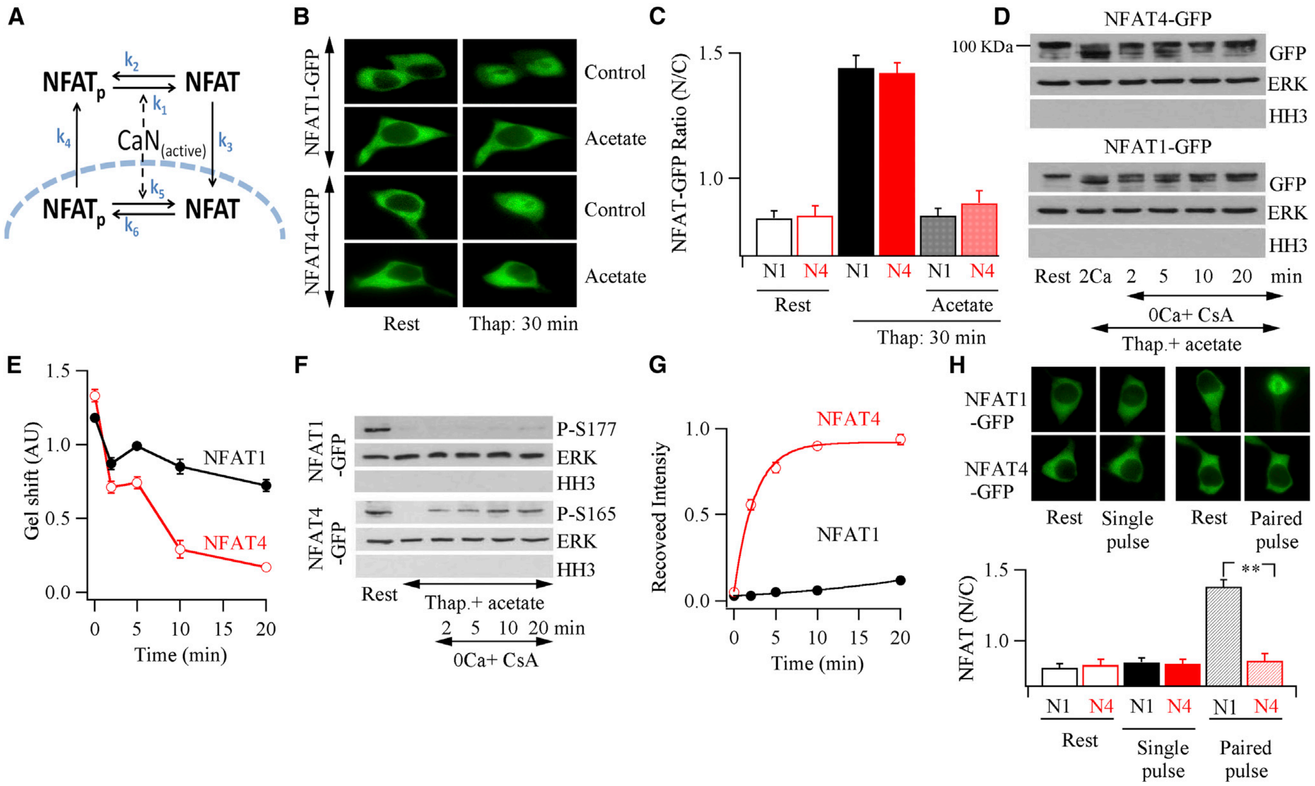

Figure 6. Cytoplasmic Rephosphorylation of NFAT Isoforms

(A) The various steps regulating NFAT dynamics are shown.

(B) Treatment with acetate (5 mM) inhibits movement of NFAT1-GFP or NFAT4-GFP to the nucleus in response to thapsigargin stimulation.

(C) Aggregate data are compared. Each bar represents mean data from three independent experiments. Acetate was applied 10 min before stimulation and then maintained throughout.

(D) NFAT isoform rephosphorylation was measured in cytoplasmic extract at different times after termination of calcineurin activity.

(E) Graphs summarize averaged data from two independent experiments, as in (D).

(F) Blots compare the kinetics of reposhosphorylation of Ser165 on NFAT4 and Ser177 on NFAT1 in cytoplasmic extract.

(G) The graph summarizes data from two independent gels, as in (F). Zero intensity at time 0 corresponds to full dephosphorylation of NFAT after thapsigargin stimulation (F).

(H) Images compare NFAT1-GFP and NFAT4-GFP nuclear accumulation in experiments using a two-pulse protocol. In the single-pulse protocol, LTC_4 was applied for 2 min in Ca^{2+}-containing external solution (pulse 1) and then washed for 38 min in Ca^{2+}-free solution (when the image was taken). In the paired-pulse protocol, after the first pulse, cells were washed for 8 min in Ca^{2+}-free solution and then a second identical LTC_4 pulse was applied for 2 min in external Ca^{2+}. Cells were then exposed to Ca^{2+}-free solution for a further 28 min before images were taken. Aggregate data from several independent experiments are shown in the histogram. Each bar is the mean of between 7 and 15 cells.

In (C), (E), (G), and (H), data are represented as mean ± SEM. See also Figure S6.

Cytokine Expression Is Differentially Regulated by Nuclear Ca^{2+} in a Cell-Specific Context

An important physiological ramification of our findings is that expression of the same gene induced by the same agonist should be differentially regulated by nuclear Ca^{2+} in different cell types, depending on which NFAT protein dominates. 16-HBE bronchial airway epithelial cells express significantly more NFAT4 than NFAT1 (Samanta et al., 2014), whereas the converse is the case in RBL-1 mast cells (data not shown). We therefore stimulated 16-HBE and RBL-1 mast cells with LTC_4 at a concentration that evoked oscillatory Ca^{2+} signals and measured expression of the interleukin-5 (IL-5) gene, which contains the GGAA core nucleotides for an NFAT binding site within the promoter (De Boer et al., 1999), in the absence or presence of nuclear $InsP_3$ buffer. In 16-HBE cells, LTC_4 stimulation led to a robust increase in transcription of IL-5 (Figure 7A) as well as in protein expression (Figure 7C). Pre-treatment with the calcineurin inhibitor cyclosporine A suppressed these responses (Figures 7A and 7C), demonstrating transcription was through the calcineurin-NFAT pathway. Expression of nuclear $InsP_3$ buffer prevented LTC_4-induced IL-5 transcription and subsequent protein expression (Figures 7A and 7C), consistent with a major role for nuclear Ca^{2+} signaling. By contrast, thapsigargin-evoked expression of IL-5 was unaffected by nuclear $InsP_3$ buffer but remained sensitive to cyclosporine A (Figures 7B and 7D). Therefore $InsP_3$ nuclear buffer does not interfere with the transcriptional process itself.

In RBL-1 cells, where NFAT1 dominates, LTC_4 also increased expression of IL-5 (Figures 7E and 7G). These responses were unaffected by nuclear $InsP_3$ buffer but were suppressed by

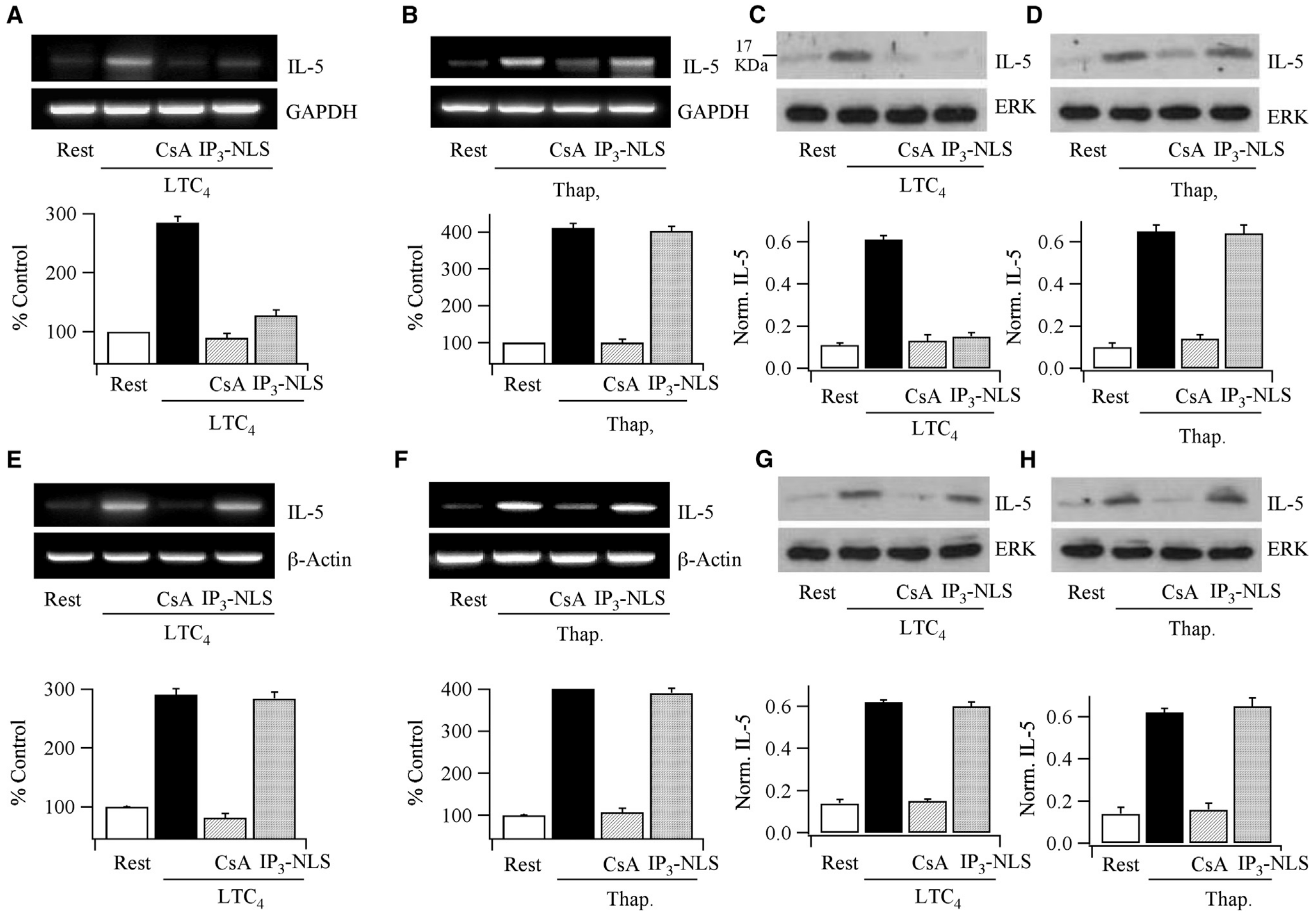

Figure 7. Cytokine Expression Is Regulated By nuclear Ca^{2+} in a Cell-Specific Context

Data in (A)–(D) are from 16-HBE bronchial epithelial cells.

(A) RT-PCR shows IL-5 expression is increased by LTC_4 and this is prevented by cyclosporine A or after expression of the IP_3-NLS buffer. Data have been normalized to rest levels (% control).

(B) As in (A), but now thapsigargin is the stimulus.

(C) Western blot compares IL-5 protein expression following stimulation with LTC_4. IL-5 expression has been normalized to ERK levels.

(D) As in (C), but the stimulus is thapsigargin. Data in (E)–(H) are from RBL-1 mast cells.

(E) LTC_4 increases IL-5 transcription, and this is unaffected by IP_3-NLS but suppressed by cyclosporine A.

(F) As in (E), but thapsigargin is the stimulus.

(G) Western blot compares IL-5 protein expression following stimulation with LTC_4.

(H) As in (F), but thapsigargin is the stimulus.

In all panels, histograms summarize data from three independent experiments. In all histograms, data are represented as mean ± SEM.

cyclosporine A (Figures 7E and 7G). Thapsigargin-induced IL-5 expression was also unaffected by the presence of nuclear $InsP_3$ buffer (Figures 7F and 7H).

Although NFAT and c-*fos* often interact synergistically to regulate gene expression, block of the non-receptor tyrosine kinase Syk, a maneuver that abolishes cysLT1 receptor activation of c-*fos* expression (Ng et al., 2012), had no effect on IL-5 gene expression to LTC_4 in RBL-1 cells (3.73-fold ± 0.10-fold increase in IL-5 mRNA relative to basal following LTC_4 stimulation in control cells and 3.69-fold ± 0.13-fold increase in the presence of Syk inhibitor, $p > 0.1$; corresponding changes in protein expression were 6.14 ± 0.42 and 6.29 ± 0.57; $p > 0.1$). Collectively, the data indicate that NFAT-driven gene expression in different cell types can be differentially regulated by nuclear Ca^{2+}. Moreover, the same gene (IL-5) can be differentially controlled by nuclear Ca^{2+}, depending on the NFAT isoform present.

Conclusion

Stimulation of cysLT1 receptors increases cytoplasmic $InsP_3$, which lowers the endoplasmic reticulum Ca^{2+} content sufficiently for CRAC channels in the plasma membrane to open, a step necessary and sufficient for NFAT1 activation (Kar et al., 2011; Kar and Parekh, 2015). The closely related NFAT4 protein additionally requires a nuclear Ca^{2+} rise (Kar and Parekh, 2015). Our new data demonstrate that cytoplasmic $InsP_3$ diffuses into the nucleus to activate $InsP_3$ receptors spanning the inner

nuclear membrane, which then increase nucleoplasmic Ca^{2+}, enabling prolonged NFAT4 activation. NFAT4 activation is therefore dependent on two spatially segregated Ca^{2+} signals: one at the plasma membrane and the other in the nucleus several micrometers away. The different requirements for nuclear Ca^{2+} and thus nuclear Ca^{2+}-calcineurin reflect the different rates of rephosphorylation of NFAT1 and NFAT4 in the nucleus, with rephosphorylation of the latter proceeding several-fold more quickly. The slower rephosphorylation rate of NFAT1 suggests this family member would be considerably better suited for imparting short-term memory to gene expression (Kar et al., 2012). Similar differences in cytoplasmic rephosphorylation rates are also important, playing a major role in the development of paired-pulse facilitation for NFAT1 only.

Remarkably, the spatially restricted Ca^{2+} signals activate NFAT4 without a role for bulk oscillatory Ca^{2+} signals in the relatively vast expanse of the enveloping cytoplasm. A corollary of this is that cytoplasmic Ca^{2+} oscillations, long considered the universal currency of physiological Ca^{2+} signaling, are not essential for the form of receptor-driven excitation-transcription coupling we describe. The oscillatory cytoplasmic Ca^{2+} signal here may be a consequence of the need to deplete the Ca^{2+} stores sufficiently for CRAC channels to activate (Bird et al., 2009; Brandman et al., 2007; Parekh et al., 1997), coupled with other roles such as the enhancement of mitochondrial ATP production that is needed to support energy-dependent Ca^{2+} responses (Hajnóczky et al., 1995).

Our data also show that expression of the same gene in response to the same agonist is controlled differently, depending on which NFAT protein dominates. Whereas NFAT1 activation is governed only by Ca^{2+} microdomains near CRAC channels, a stronger stimulus is needed for NFAT4 nuclear accumulation, because $InsP_3$ has to overcome breakdown by cytoplasmic enzymes to enter the nucleus and release nuclear Ca^{2+}. The dependence of different NFAT proteins on different stimulus intensities (Kar and Parekh, 2015) will both increase the bandwidth of excitation-transcription coupling and recruit different gene expression profiles. Finally, our results demonstrate how a ubiquitous and globally diffusible second messenger like $InsP_3$ can nevertheless be used to activate different transcription factor isoforms through generation of coordinated yet spatially distinct subcellular Ca^{2+} signals.

EXPERIMENTAL PROCEDURES

Cells
HEK293 (Kar et al., 2011), RBL-1 (Kar et al., 2012), and 16-HBE (Samanta et al., 2014) cells were cultured as described. HEK293 cells were used in Figures 1–6 and RBL-1 and 16-HBE cells in Figure 7.

cDNA Constructs and Transfection
HEK293 and 16-HBE cells were transfected using lipofectamine, as described (Kar et al., 2011; Samanta et al., 2014). RBL-1 cells were transfected using the Amaxa system (Kar et al., 2012). cDNA for NFAT4-GFP, parvalbumin constructs, and cytoplasmic GCaMP3 were from Addgene (deposited by Dr. Anjana Rao, Dr. Anton Bennett, and Dr. Jin Zhang, respectively). Nuclear and cytoplasmic $InsP_3$ buffer were gifts from Dr. Maria de Fatima Leite, Federal University of Minas Gerais, Brazil. NFAT1-GFP was a gift from Dr. Jennings Worley. Alterations to these constructs were done by Mutagenex. All plasmids were used at 1 μg, and experiments commenced 24–48 hr after transfection.

NFAT Migration
Tagged NFAT protein levels in the cytoplasm and nucleus were measured using the IMAGO charge-coupled device camera-based system from TILL Photonics, with a × 100 oil immersion objective (numerical aperture 1.3) (Kar and Parekh, 2015). Regions of interest of identical size were drawn in the cytoplasm and nucleus of each cell, and fluorescence was computed. Nuclear localization was confirmed by costaining with the nuclear dye DAPI. Unless otherwise indicated, we calculated the nuclear/cytoplasmic ratio of tagged NFAT as a function of stimulus time.

Cytoplasmic and Nuclear Ca^{2+} Measurements
Cytoplasmic and nuclear Ca^{2+} measurements, using GCaMP3 constructs, were carried out at room temperature by using the IMAGO charge-coupled device camera-based system from TILL Photonics (Kar et al., 2012). Cells were placed in standard external solution composed of 145 mM NaCl, 2.8 mM KCl, 2 mM $CaCl_2$, 2 mM $MgCl_2$, 10 mM D-glucose, and 10 mM HEPES at pH 7.4, with NaOH and were excited at 489 nm (typically 100 ms exposure), at 0.5 Hz. Fura-2 experiments (Figure S1) were carried out as described (Kar et al., 2012). Ca^{2+} signals are plotted either as F/F0, which denotes the increase in fluorescence divided by the resting level for the GCaMP3 reporters, or *R*, which denotes the 356/380 nm ratio, for Fura-2. Simultaneous measurements of cytoplasmic and nuclear Ca^{2+} were performed with fluo-4, as described (Kar and Parekh, 2015).

Immunogold Electron Microscopy
Briefly, cells were fixed with 3% paraformaldehyde/0.05% glutaraldehyde in 0.1 M phosphate buffer for 10 min, stained with uranyl acetate (2% w/v in distilled water), dehydrated through increasing concentrations of methanol (70%–100%), and embedded in LR Gold (London Resin Company). Ultrathin sections (50–80 nm) were prepared by use of a Reichert Ultracut S ultramicrotome (Leica), mounted on 200-mesh nickel grids, incubated at room temperature for 2 hr with a monoclonal anti-$InsP_3$ receptor type I antibody (dilution 1:100; Abcam) and for 1 hr with anti-mouse IgG −15 nm gold complex (British Biocell International). All antisera were diluted in 0.1 M phosphate buffer containing 0.1% egg albumin (pH 7.2). As a control the primary antibody was replaced with non-immune sera. After immunolabeling, sections were lightly counterstained with lead citrate and uranyl acetate and examined with a JEOL transmission electron microscope (JEM-1010, JEOL), and images were collected with a Gatan Orius digital camera (Gatan).

Cytoplasmic Staining
HEK293 cells were fixed with paraformaldehyde (4%) at room temperature and permeabilized with PBS/Triton X-100 (0.5%). Cells were then stained overnight with anti-$InsP_3$ receptor type I antibody (Abcam), used at 1:100 dilution. Primary antibody was then visualized with Alexa Fluor 488 goat anti-mouse secondary antibody (1:500)

Western Blot
Total cell lysates (40 μg) were analyzed by SDS-PAGE on a 10% or 12% (IL-5) gel. Membranes were blocked with 5% nonfat dry milk in PBS plus 0.1% Tween 20 (PBST) buffer for 1 hr at room temperature (Kar and Parekh, 2015). For phospho-NFAT blots, we used TBST (Tris buffer saline with Tween 20). Membranes were washed with PBST three times and then incubated with primary antibody for 24 hr at 4°C. Antibodies against ERK (1:5,000, rabbit polyclonal; Santa Cruz Biotechnology), GFP (1:2,000, rabbit polyclonal; Cell Signaling), HH3 (Histone H3; 1:5,000, rabbit polyclonal; Abcam), $InsP_3$ type I receptor (1:1,000, mouse monoclonal; Abcam), anti-phospho Ser177 NFAT1 (Santa Cruz; 1:2,000 dilution), anti-phospho Ser165 NFAT4 (Abcam; 1:2,000), and IL-5 (Santa Cruz; 1:500) were used. Secondary antibodies for rabbit or mouse were used at 1:5,000 or 1:3,000 dilutions. The membranes were then washed with PBST again and incubated with peroxidase-linked anti-rabbit IgG from Santa Cruz Biotechnology or anti-mouse IgG from BD Bioscience for 1 hr at room temperature. After washing with PBST, the bands were detected by an enhanced chemiluminescence (ECL) plus western blotting detection system (Amersham Biosciences). Blots were analyzed by UN-Scan software. Nuclear and cytoplasmic extracts were separated as before (Kar and Parekh, 2015) and

assessed by the presence of HH3 in nuclear but not cytoplasmic extract and ERK2 in the cytoplasmic but not nuclear extract.

Gel shifts were quantified in ImageJ. Intensity of the region corresponding to the dephosphorylated state (lower band; obtained after stimulation with thapsigargin in 2 mM external Ca^{2+} for 20 min; see relevant figure legends) was measured and the region (same dimensions and position) was transposed to adjacent lanes. Data are presented relative to the intensity of the dephosphorylated state. Gel analysis was carried out on blots from at least two independent experiments.

RT-PCR

16-HBE cells transfected with cys LT1 receptors or wild-type RBL-1 cells were stimulated with thapsigargin or LTC_4 in DMEM for 20 min, washed five times with DMEM (without thapsigargin or LTC_4), and then kept in an incubator at 37°C for 6–8 hr. Cells were then washed with PBS, and total RNA was extracted using an RNeasy Mini Kit (QIAGEN). RNA was quantified spectrophotometrically by absorbance at 260 nm. Total RNA (1 μg) was reverse transcribed using the iScriptTM cDNA Synthesis Kit (Bio-Rad), according to the manufacturer's instructions. Two-microliter samples (cDNA), from RT-PCR, were subjected to polymerase chain reaction (PCR) with the specific primers and GoTaq Green master mix (Promega). The mRNA levels were normalized to GAPDH for 16-HBE cell and β-actin for RBL-1 cells.

IL-5 (human): Forward-5′-CTGAGGATTCCTGTTCCTGT-3′; Reverse-5′-CAACTTTCTATTATCCACTC-3′
GAPDH (human): Forward-5′-GGTATCGTGGAAGGACTCAT-3′; Reverse-5′-CCACCCTGTTGCTGTAGCCAAATTC-3′
IL-5 (rat): Forward-5′-AGACCGGTCATACATGCACA-3′; Reverse-5′-TCG TCTCATTGCTCGTCAAC-3′
β-actin (rat): Forward- 5′-TTGTAACCAACTGGGACGATATG-3′; Reverse-5′-GATCTTGATCTTCATGGTGCTAGG-3′

Mathematical Modeling

The details of mathematical modeling are explained in the Supplemental Information.

Statistical Analysis

Data are presented as the mean ± SEM. Statistical significance was determined by using Student's t test. *p < 0.05 and **p < 0.01.

ACCESSION NUMBERS

Original imaging data have been deposited to Mendeley Data and are available at http://dx.doi.org/10.17632/v8sfxkh9pw.1.

SUPPLEMENTAL INFORMATION

Supplemental Information includes six figures, one table, supplemental equations, and a zipped data file containing calcineurin code and can be found with this article online at http://dx.doi.org/10.1016/j.molcel.2016.11.011.

AUTHOR CONTRIBUTIONS

P.K. and A.B.P. designed experiments. P.K. carried out the experiments. Electron microscopy was conducted by H.C.C. Analyses of data were performed by P.K. and A.B.P. Discussion of the model and how to identify various rate constants involved P.K., G.R.M., and A.B.P. The modeling was executed by G.R.M. A.B.P. wrote the manuscript.

ACKNOWLEDGMENTS

This work was supported by an MRC Programme grant to A.B.P. (grant number LO1047X). G.R.M is supported by a Sir Henry Dale Fellowship jointly funded by the Wellcome Trust and Royal Society (grant number 101222/Z/13/Z). We thank Dr. Juris Galvanovskis for help with confocal microscopy measurements of nuclear Ca^{2+}.

Received: March 25, 2016
Revised: August 22, 2016
Accepted: November 3, 2016
Published: November 17, 2016

REFERENCES

Allbritton, N.L., Meyer, T., and Stryer, L. (1992). Range of messenger action of calcium ion and inositol 1,4,5-trisphosphate. Science *258*, 1812–1815.

Alonso, M.T., and García-Sancho, J. (2011). Nuclear Ca(2+) signalling. Cell Calcium *49*, 280–289.

Bazzazi, H., Sang, L., Dick, I.E., Joshi-Mukherjee, R., Yang, W., and Yue, D.T. (2015). Novel fluorescence resonance energy transfer-based reporter reveals differential calcineurin activation in neonatal and adult cardiomyocytes. J. Physiol. *593*, 3865–3884.

Bettelli, E., Dastrange, M., and Oukka, M. (2005). Foxp3 interacts with nuclear factor of activated T cells and NF-kappa B to repress cytokine gene expression and effector functions of T helper cells. Proc. Natl. Acad. Sci. USA *102*, 5138–5143.

Bird, G.S., Hwang, S.Y., Smyth, J.T., Fukushima, M., Boyles, R.R., and Putney, J.W.J., Jr. (2009). STIM1 is a calcium sensor specialized for digital signaling. Curr. Biol. *19*, 1724–1729.

Bootman, M.D., Fearnley, C., Smyrnias, I., MacDonald, F., and Roderick, H.L. (2009). An update on nuclear calcium signalling. J. Cell Sci. *122*, 2337–2350.

Brandman, O., Liou, J., Park, W.S., and Meyer, T. (2007). STIM2 is a feedback regulator that stabilizes basal cytosolic and endoplasmic reticulum Ca2+ levels. Cell *131*, 1327–1339.

De Boer, M.L., Mordvinov, V.A., Thomas, M.A., and Sanderson, C.J. (1999). Role of nuclear factor of activated T cells (NFAT) in the expression of interleukin-5 and other cytokines involved in the regulation of hemopoetic cells. Int. J. Biochem. Cell Biol. *31*, 1221–1236.

Di Capite, J., Ng, S.-W., and Parekh, A.B. (2009). Decoding of cytoplasmic Ca(2+) oscillations through the spatial signature drives gene expression. Curr. Biol. *19*, 853–858.

Echevarría, W., Leite, M.F., Guerra, M.T., Zipfel, W.R., and Nathanson, M.H. (2003). Regulation of calcium signals in the nucleus by a nucleoplasmic reticulum. Nat. Cell Biol. *5*, 440–446.

Fornerod, M., Ohno, M., Yoshida, M., and Mattaj, I.W. (1997). CRM1 is an export receptor for leucine-rich nuclear export signals. Cell *90*, 1051–1060.

Gerasimenko, O.V., Gerasimenko, J.V., Tepikin, A.V., and Petersen, O.H. (1996). Calcium transport pathways in the nucleus. Pflugers Arch. *432*, 1–6.

Gwack, Y., Sharma, S., Nardone, J., Tanasa, B., Iuga, A., Srikanth, S., Okamura, H., Bolton, D., Feske, S., Hogan, P.G., and Rao, A. (2006). A genome-wide Drosophila RNAi screen identifies DYRK-family kinases as regulators of NFAT. Nature *441*, 646–650.

Hajnóczky, G., Robb-Gaspers, L.D., Seitz, M.B., and Thomas, A.P. (1995). Decoding of cytosolic calcium oscillations in the mitochondria. Cell *82*, 415–424.

Humbert, J.P., Matter, N., Artault, J.C., Köppler, P., and Malviya, A.N. (1996). Inositol 1,4,5-trisphosphate receptor is located to the inner nuclear membrane vindicating regulation of nuclear calcium signaling by inositol 1,4,5-trisphosphate. Discrete distribution of inositol phosphate receptors to inner and outer nuclear membranes. J. Biol. Chem. *271*, 478–485.

Ishiguro, K., Ando, T., Maeda, O., Watanabe, O., and Goto, H. (2011). Cutting edge: tubulin α functions as an adaptor in NFAT-importin β interaction. J. Immunol. *186*, 2710–2713.

Kar, P., and Parekh, A.B. (2015). Distinct spatial Ca2+ signatures selectively activate different NFAT transcription factor isoforms. Mol. Cell *58*, 232–243.

Kar, P., Nelson, C., and Parekh, A.B. (2011). Selective activation of the transcription factor NFAT1 by calcium microdomains near Ca2+ release-activated Ca2+ (CRAC) channels. J. Biol. Chem. *286*, 14795–14803.

Kar, P., Nelson, C., and Parekh, A.B. (2012). CRAC channels drive digital activation and provide analog control and synergy to Ca(2+)-dependent gene regulation. Curr. Biol. *22*, 242–247.

Kehlenbach, R.H., Dickmanns, A., and Gerace, L. (1998). Nucleocytoplasmic shuttling factors including Ran and CRM1 mediate nuclear export of NFAT In vitro. J. Cell Biol. *141*, 863–874.

Lin, C., Hajnóczky, G., and Thomas, A.P. (1994). Propagation of cytosolic calcium waves into the nuclei of hepatocytes. Cell Calcium *16*, 247–258.

Lipp, P., Thomas, D., Berridge, M.J., and Bootman, M.D. (1997). Nuclear calcium signalling by individual cytoplasmic calcium puffs. EMBO J. *16*, 7166–7173.

Loh, C., Shaw, K.T.-Y., Carew, J., Viola, J.P.B., Luo, C., Perrino, B.A., and Rao, A. (1996). Calcineurin binds the transcription factor NFAT1 and reversibly regulates its activity. J. Biol. Chem. *271*, 10884–10891.

Macián, F., López-Rodríguez, C., and Rao, A. (2001). Partners in transcription: NFAT and AP-1. Oncogene *20*, 2476–2489.

Macián, F., García-Cózar, F., Im, S.H., Horton, H.F., Byrne, M.C., and Rao, A. (2002). Transcriptional mechanisms underlying lymphocyte tolerance. Cell *109*, 719–731.

Martinez, G.J., Pereira, R.M., Äijö, T., Kim, E.Y., Marangoni, F., Pipkin, M.E., Togher, S., Heissmeyer, V., Zhang, Y.C., Crotty, S., et al. (2015). The transcription factor NFAT promotes exhaustion of activated $CD8^+$ T cells. Immunity *42*, 265–278.

Mogami, H., Nakano, K., Tepikin, A.V., and Petersen, O.H. (1997). Ca2+ flow via tunnels in polarized cells: recharging of apical Ca2+ stores by focal Ca2+ entry through basal membrane patch. Cell *88*, 49–55.

Müller, M.R., and Rao, A. (2010). NFAT, immunity and cancer: a transcription factor comes of age. Nat. Rev. Immunol. *10*, 645–656.

Ng, S.W., Bakowski, D., Nelson, C., Mehta, R., Almeyda, R., Bates, G., and Parekh, A.B. (2012). Cysteinyl leukotriene type I receptor desensitization sustains Ca2+-dependent gene expression. Nature *482*, 111–115.

Okamura, H., Aramburu, J., García-Rodríguez, C., Viola, J.P., Raghavan, A., Tahiliani, M., Zhang, X., Qin, J., Hogan, P.G., and Rao, A. (2000). Concerted dephosphorylation of the transcription factor NFAT1 induces a conformational switch that regulates transcriptional activity. Mol. Cell *6*, 539–550.

Parekh, A.B., Fleig, A., and Penner, R. (1997). The store-operated calcium current I(CRAC): nonlinear activation by InsP3 and dissociation from calcium release. Cell *89*, 973–980.

Rao, A., Luo, C., and Hogan, P.G. (1997). Transcription factors of the NFAT family: regulation and function. Annu. Rev. Immunol. *15*, 707–747.

Samanta, K., Bakowski, D., and Parekh, A.B. (2014). Key role for store-operated Ca2+ channels in activating gene expression in human airway bronchial epithelial cells. PLoS ONE *9*, e105586.

Saucerman, J.J., and Bers, D.M. (2008). Calmodulin mediates differential sensitivity of CaMKII and calcineurin to local Ca2+ in cardiac myocytes. Biophys. J. *95*, 4597–4612.

Thomas, A.P., Bird, G.S., Hajnóczky, G., Robb-Gaspers, L.D., and Putney, J.W.J., Jr. (1996). Spatial and temporal aspects of cellular calcium signaling. FASEB J. *10*, 1505–1517.

Yissachar, N., Sharar Fischler, T., Cohen, A.A., Reich-Zeliger, S., Russ, D., Shifrut, E., Porat, Z., and Friedman, N. (2013). Dynamic response diversity of NFAT isoforms in individual living cells. Mol. Cell *49*, 322–330.

Zima, A.V., Bare, D.J., Mignery, G.A., and Blatter, L.A. (2007). IP3-dependent nuclear Ca2+ signalling in the mammalian heart. J. Physiol. *584*, 601–611.

Molecular Cell, Volume *64*

Supplemental Information

Control of NFAT Isoform Activation and NFAT-Dependent Gene Expression through Two Coincident and Spatially Segregated Intracellular Ca^{2+} Signals

Pulak Kar, Gary R. Mirams, Helen C. Christian, and Anant B. Parekh

Figure S1

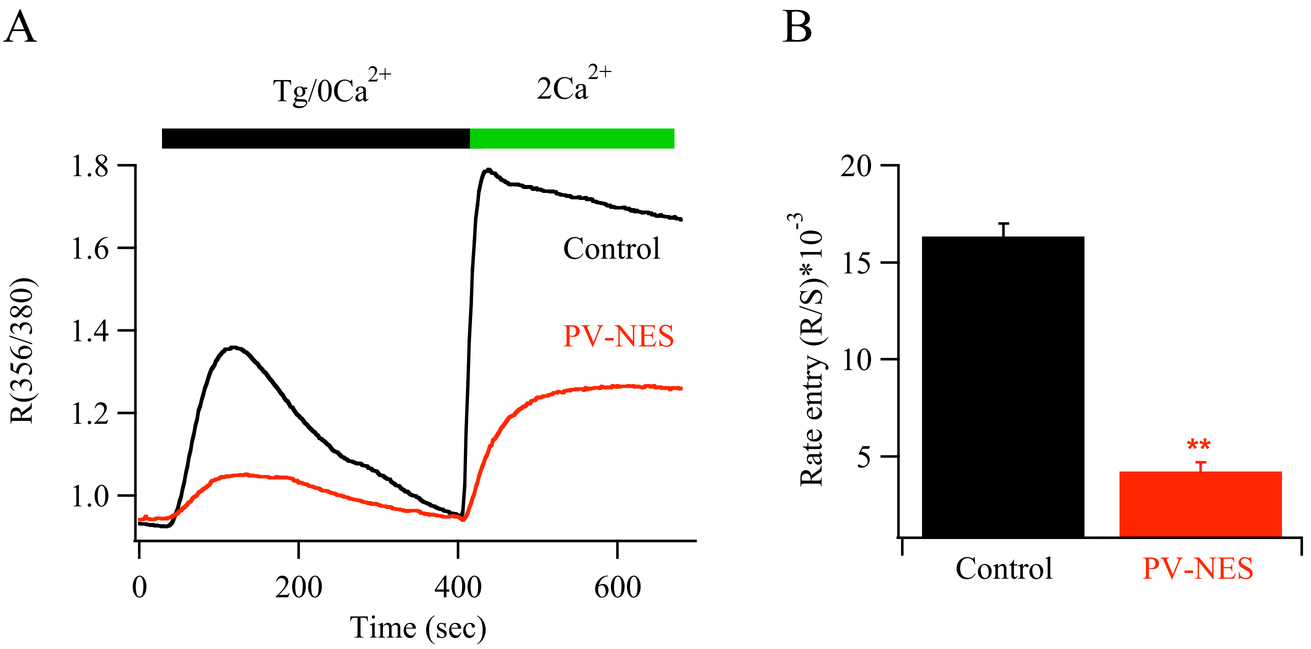

A
Tg/0Ca2+
2Ca2+
1.8
1.6
1.4
1.2
1.0
R(356/380)
Control
PV-NES
0
200
400
600
Time (sec)
B
20
15
10
5
Rate entry (R/S)*10-3
**
Control
PV-NES

Figure S2

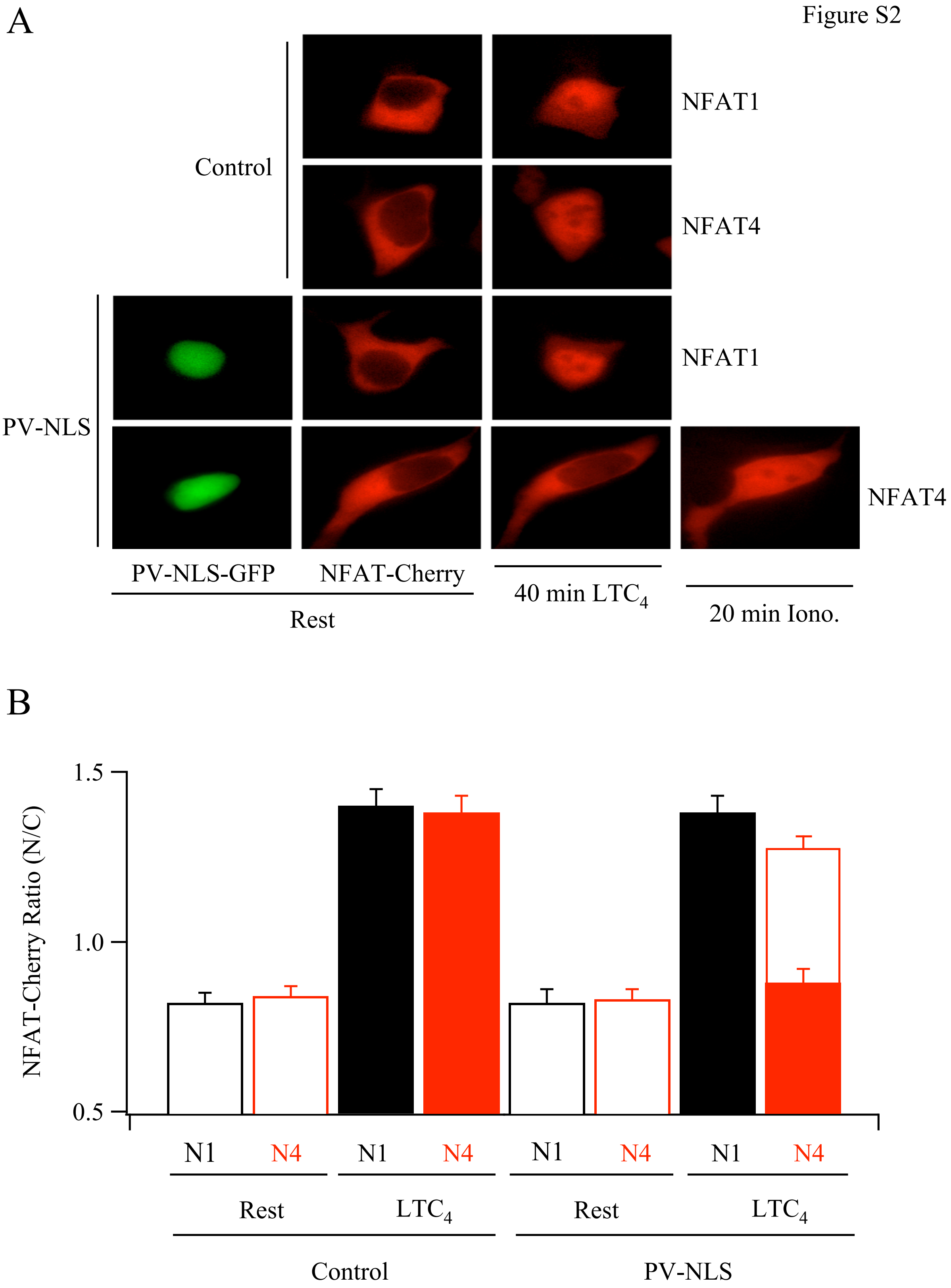
A
Control
NFAT1
NFAT4
PV-NLS
NFAT1
NFAT4
PV-NLS-GFP
NFAT-Cherry
Rest
40 min LTC4
20 min Iono.
B
NFAT-Cherry Ratio (N/C)
1.5
1.0
0.5
N1
N4
N1
N4
N1
N4
N1
N4
Rest
LTC4
Rest
LTC4
Control
PV-NLS

Figure S3

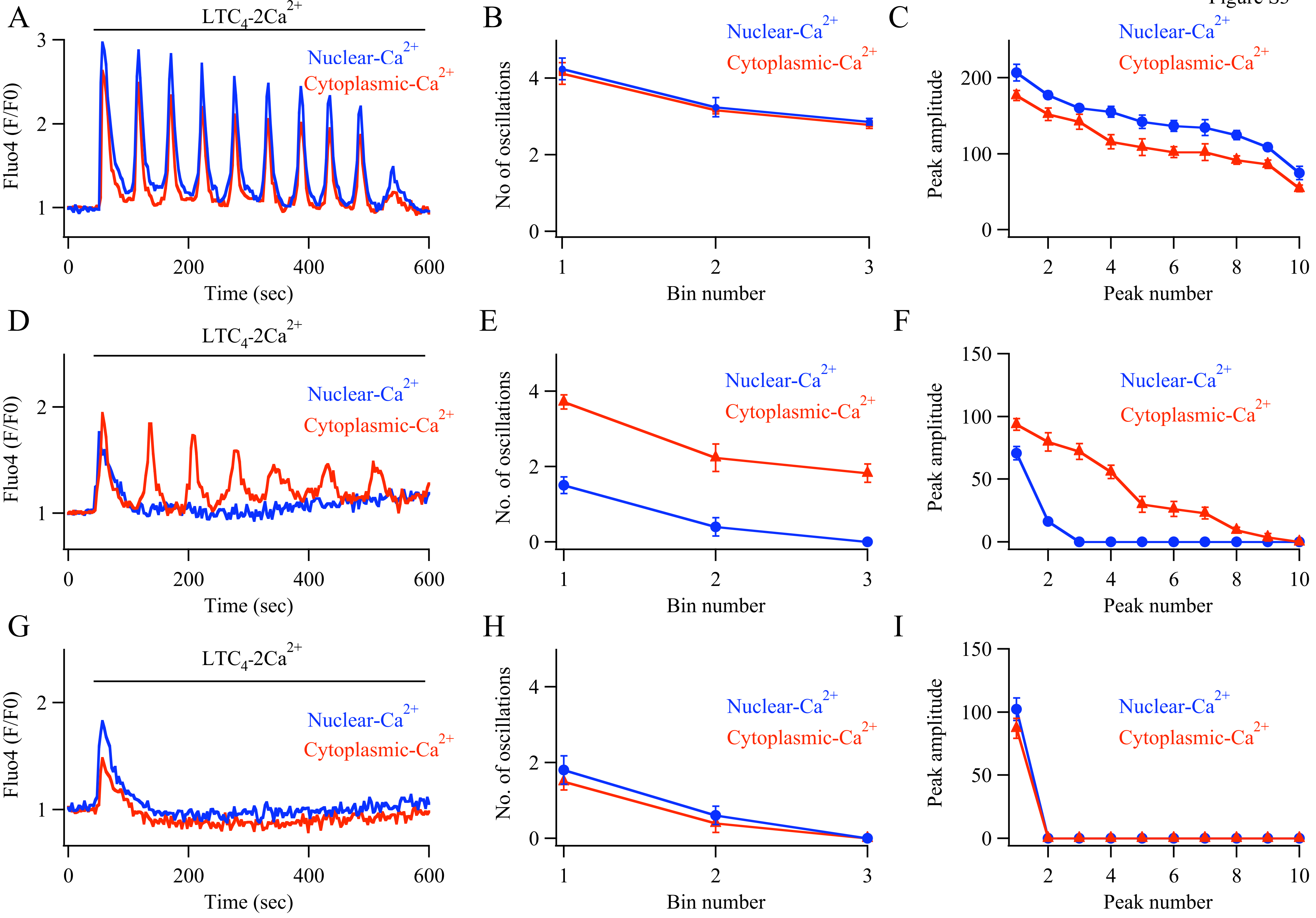
A
LTC4-2Ca2+
Nuclear-Ca2+
Cytoplasmic-Ca2+
Fluo4 (F/F0)
Time (sec)
B
No of oscillations
Bin number
C
Peak amplitude
Peak number
D
LTC4-2Ca2+
Nuclear-Ca2+
Cytoplasmic-Ca2+
Fluo4 (F/F0)
Time (sec)
E
No. of oscillations
Bin number
F
Peak amplitude
Peak number
G
LTC4-2Ca2+
Nuclear-Ca2+
Cytoplasmic-Ca2+
Fluo4 (F/F0)
Time (sec)
H
No. of oscillations
Bin number
I
Peak amplitude
Peak number

Figure S4

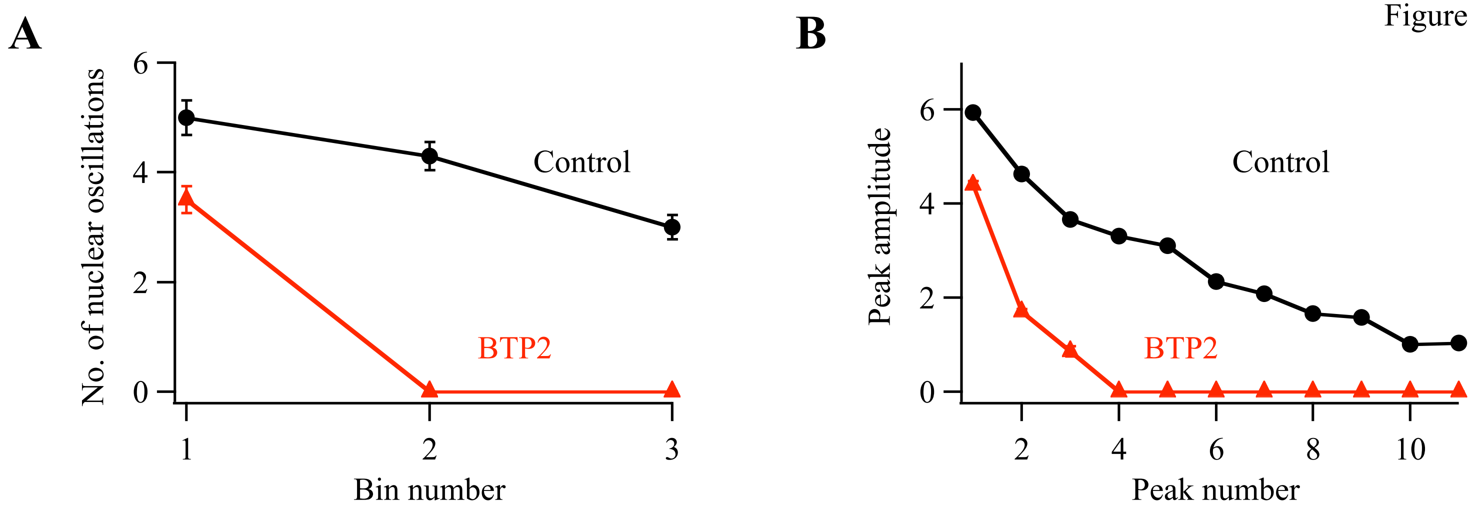
A
No. of nuclear oscillations
6
4
2
0
1
2
3
Bin number
Control
BTP2
B
Peak amplitude
6
4
2
0
2
4
6
8
10
Peak number
Control
BTP2

Figure S5

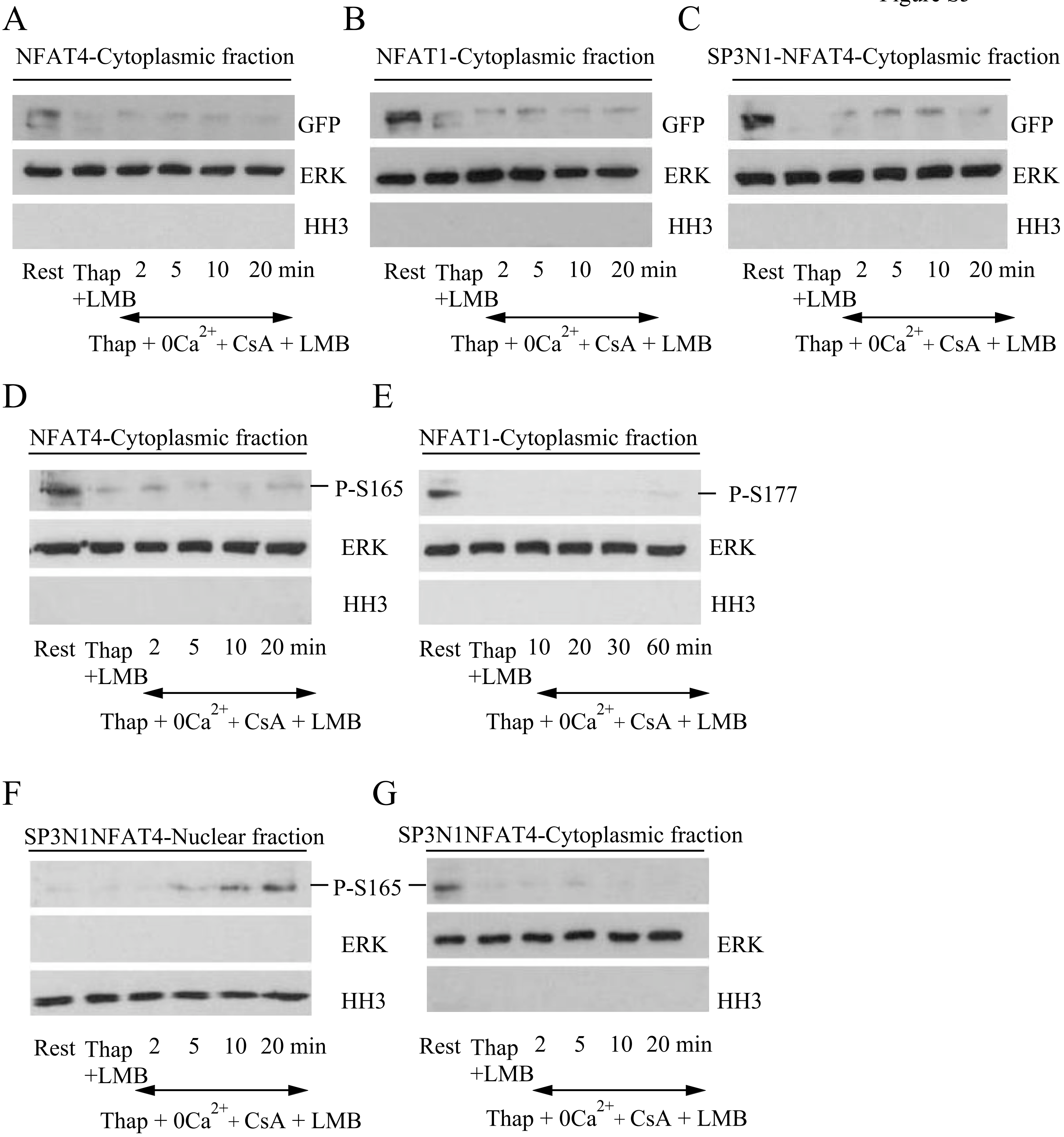
A
NFAT4-Cytoplasmic fraction
GFP
ERK
HH3
Rest Thap +LMB 2 5 10 20 min
Thap + 0Ca2+ + CsA + LMB
B
NFAT1-Cytoplasmic fraction
GFP
ERK
HH3
Rest Thap +LMB 2 5 10 20 min
Thap + 0Ca2+ + CsA + LMB
C
SP3N1-NFAT4-Cytoplasmic fraction
GFP
ERK
HH3
Rest Thap +LMB 2 5 10 20 min
Thap + 0Ca2+ + CsA + LMB
D
NFAT4-Cytoplasmic fraction
P-S165
ERK
HH3
Rest Thap +LMB 2 5 10 20 min
Thap + 0Ca2+ + CsA + LMB
E
NFAT1-Cytoplasmic fraction
P-S177
ERK
HH3
Rest Thap +LMB 10 20 30 60 min
Thap + 0Ca2+ + CsA + LMB
F
SP3N1NFAT4-Nuclear fraction
P-S165
ERK
HH3
Rest Thap +LMB 2 5 10 20 min
Thap + 0Ca2+ + CsA + LMB
G
SP3N1NFAT4-Cytoplasmic fraction
P-S165
ERK
HH3
Rest Thap +LMB 2 5 10 20 min
Thap + 0Ca2+ + CsA + LMB

Figure S6

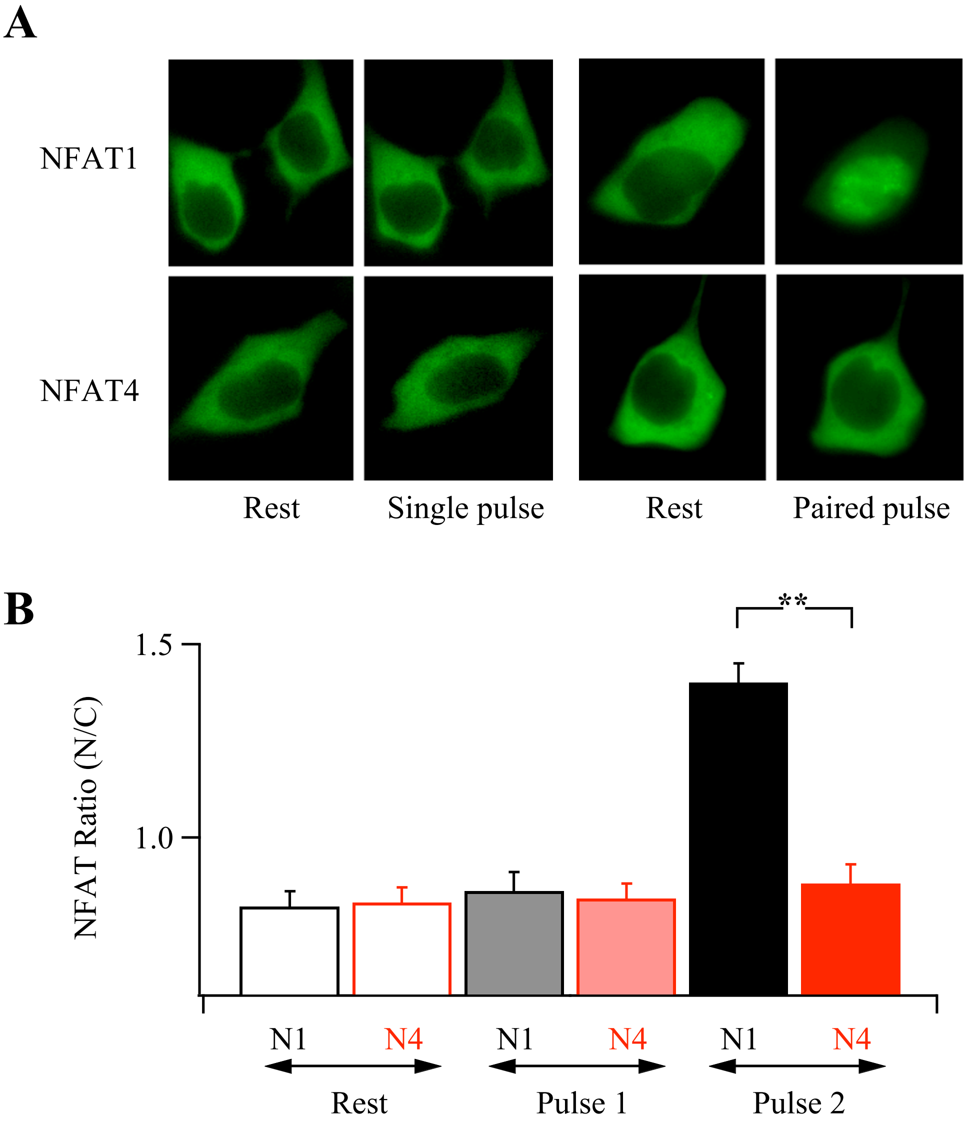
A
NFAT1
NFAT4
Rest
Single pulse
Rest
Paired pulse
B
NFAT Ratio (N/C)
1.5
1.0
**
N1
N4
N1
N4
N1
N4
Rest
Pulse 1
Pulse 2

Figure S1, related to Figure 1. PV-NES reduces intracellular Ca^{2+} signals in fura 2-loaded cells. A, Expression of parvalbumin tagged with a nuclear export sequence (PV-NES) reduces the cytoplasmic Ca^{2+} rise following both Ca^{2+} release and then Ca^{2+} influx after thapsigargin stimulation. Thapsigargin was applied in Ca^{2+}-free solution (labelled Tg/0Ca^{2+}). B, The rate of rise of cytoplasmic Ca^{2+} following Ca^{2+} readmission to cells pre-treated with thapisgargin (2 μM) for 10 minutes in Ca^{2+}-free external solution is compared between control (mock-transfected) cells and cells expressing PV-NES. Each bar is the average of > 26 cells. ** denotes $p<0.01$. In Panel B, data are represented as mean±SEM.

Figure S2, related to Figure 1. Nuclear-targeted parvalbumin (PV-NLS) inhibits NFAT4 but not NFAT1 nuclear accumulation. A, Images compare NFAT nuclear migration between control cells and cells expressing PV-NLS. Upper images, marked control, show the distribution of NFAT1- or NFAT4-cherry at rest, and then after stimulation with LTC_4 (160 nM for 40 minutes). Nuclear migration occurs for both NFAT proteins. The lower images show the effects of PV-NLS-GFP on NFAT-cherry migration. PV-NLS-GFP localized to the nucleus (left hand panels) and NFAT1-cherry or NFAT4-cherry was cytoplasmic at rest. LTC_4 induced movement of NFAT1 but not NFAT4 in cells expressing PV-NLS, both measured 40 minutes after stimulation. Movement of NFAT4 was subsequently recovered by ionomycin application (20 minutes; 2 μM). B, Aggregate data from several experiments as in Panel A are summarised. N1 denotes NFAT1 and N4 NFAT4. Each bar is the mean of between 9 and 17 cells. Open bar above LTC_4/N4 bar in PV-NLS shows the extent of rescue by ionomycin in these cells, applied 40 minutes after LTC_4. In Panel B, data are represented as mean±SEM.

Figure S3, related to Figure 3. Simultaneous measurements of cytoplasmic and nuclear Ca^{2+} signals in fluo-4-loaded cells. A, Ca^{2+} oscillations in the cytoplasm and nucleus to LTC_4 in a control cell are shown. B-C, Aggregate data comparing the number of oscillations per 200 seconds bin (B) and peak amplitude of each oscillation (C) are

depicted. D-F, As in panels A-C but cells now expressed IP_3-NLS. G-I, As in panels A-C but cells expressed both IP_3-NES and IP_3-NLS. In the graphs, data are represented as mean±SEM.

Figure S4, related to Figure 4. Nuclear Ca^{2+} oscillations induced by LTC_4 run down quickly when CRAC channels are blocked. A, Aggregate data compare the number of oscillations per 200 seconds bin for the conditions shown. B, The peak amplitude of each oscillation in control cells and cells exposed to BTP2 are compared. Each point is the mean of between 17 and 25 cells. Cells were exposed to 10 μM BTP2 for 10 minutes prior to stimulation with LTC_4. In the graphs, data are represented as mean±SEM.

Figure S5, related to Figure 5. Leptomycin B (LMB) traps NFAT in the nucleus for up to 20 minutes. A, Gel shows that after stimulation with thapsigargin in LMB, very little NFAT4-GFP remains in the cytoplasmic fraction. Even after initiation of nuclear export (Ca^{2+}-free external solution containing cyclosporine A), very little NFAT4-GFP returns to the cytoplasm in the presence of LMB for up to 20 minutes. B, As in panel A but NFAT1-GFP was expressed instead. C, As in panel A but the SP3N1-NFAT4-GFP construct was expressed instead. D, Phosphorylated serine 165 on NFAT4 is prominent in the cytoplasmic fraction at rest but very little remains in the cytoplasm after stimulation with thapsigargin in LMB and no detectable recovery of cytoplasmic phosphorylated serine 165 occurs after nuclear export is initiated in the presence of LMB. E, As in panel D but NFAT1 was expressed and phosphorylation was assessed on serine 177. F, Nuclear extract from cells expressing SP3N1-NFAT4 shows undetectable phosphorylated serine 165 at rest or after thapsigargin stimulation (30 minutes in external Ca^{2+}), but a time-dependent increase following exposure to Ca^{2+}-free external solution supplemented with cyclosporine A in LMB. G, As in panel F but the cytoplasmic fraction is shown. Note the lack of SP3N1-NFAT4 in the cytoplasm for up to 20 minutes in the presence of LMB after nuclear export had been initiated, confirming LMB blocks nuclear exit.

Figure S6, related to Figure 6. NFAT1 but not NFAT4 exhibits paired pulse facilitation to Ca^{2+} pulses following thapsigargin stimulation. A, Images compare NFAT1-GFP and NFAT4-GFP distribution at rest,

after one Ca^{2+} pulse (single pulse) and after a paired pulse protocol. Cells were pre-treated with thapsigargin in Ca^{2+}-free solution for 15 minutes to deplete stores. Single pulse denotes NFAT distribution following a Ca^{2+} pulse for two minutes followed by exposure to Ca^{2+}-free solution for 38 minutes. Paired pulse represents two identical pulses (two minutes each), spaced 8 minutes apart. Cells were in Ca^{2+}-free solution during the interpulse interval and then for 28 minutes after the second pulse had been applied (total of 38 minutes after the first pulse). B, Aggregate data for the conditions indicated are compared. ** denotes $p < 0.01$. N1 denotes NFAT1 and N4 NFAT4. Data are represented as mean±SEM.

Table S1, related to Figure 4: Parameter values for mathematical model, these are taken from Saucerman et al. (2008) and we keep the same notation, that paper also provides literature references for these values. Note $[CaN]_{Total}$ is varied in some of our simulations, and $[Ca^{2+}]$ varies as an input into all simulations.

Rate constant	Value	Units
k_{20}	10	s^{-1}
K_{d02}	3.1	μM
k_{02}	k_{20}/K_{d02}	$\mu M^{-2}\ s^{-1}$
k_{42}	500	s^{-1}
K_{d24}	24	μM
k_{24}	k_{42}/K_{d24}	$\mu M^{-2}\ s^{-1}$
$k_{CaN4Off}$	2.0	s^{-1}
k_{CaN4On}	46	$\mu M^{-1}\ s^{-1}$
$k_{CaNCaOff}$	1.0	s^{-1}
$k_{CaNCaOn}$	$k_{CaNCaOff}/0.5$	$\mu M^{-2}\ s^{-1}$
$[CaN]_{Total}$	3.62	μM
$[CaM]_{Total}$	6	μM

Zipped data file (related to Figure 4) executes the model based on the parameters in Table 1.The model, in cartoon form, is:

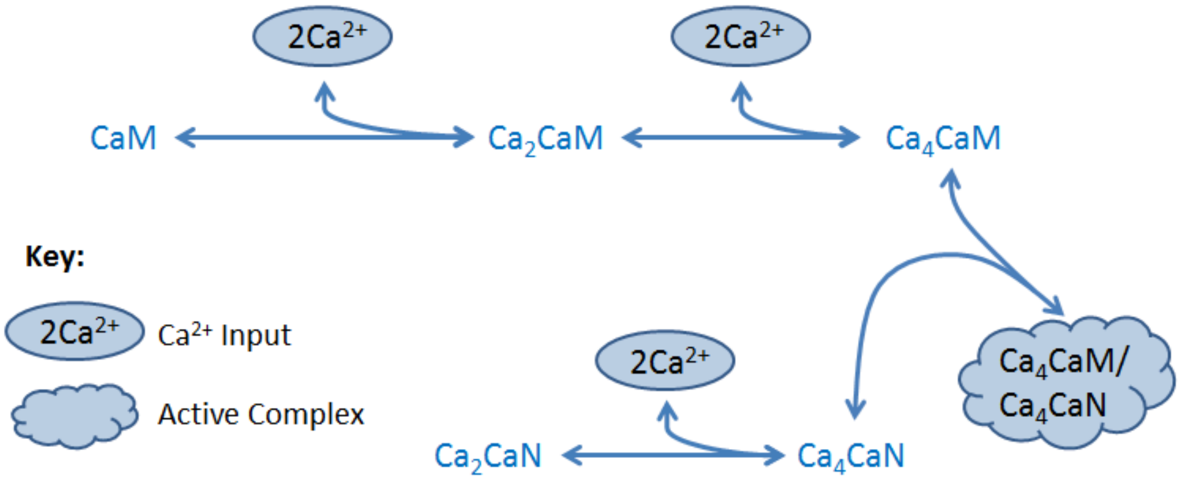

This schematic is the reduced version of the Saucerman et al. (2008), as used by Bazzazi et al. (2015).

The following equations were proposed by Bazzazi et al. (2015) as a simplification of those found in an earlier model of calcineurin activation (Saucerman & Bers 2008). We have followed Bazzazi et al. in using these simpler equations. The equations are derived using mass-action kinetics, with two conservation relationships eliminating the ordinary differential equations (ODEs) for [CaM] and [Ca_2CaN], leaving the following differential & algebraic equation (DAE) system:

$$
\begin{aligned}
[\mathrm{CaM}] &= [\mathrm{CaM}]_{\mathrm{Total}} - \left([\mathrm{Ca_2CaM}] + [\mathrm{Ca_4CaM}] + [\mathrm{Ca_4CaMCa_4CaN}]\right), \\
[\mathrm{Ca_2CaN}] &= [\mathrm{CaN}]_{\mathrm{Total}} - \left([\mathrm{Ca_4CaN}] + [\mathrm{Ca_4CaMCa_4CaN}]\right), \\
\frac{\mathrm{d}[\mathrm{Ca_2CaM}]}{\mathrm{d}t} &= k_{02}[\mathrm{CaM}][\mathrm{Ca^{2+}}]^2 - k_{20}[\mathrm{Ca_2CaM}] + k_{42}[\mathrm{Ca_4CaM}] - k_{24}[\mathrm{Ca_2CaM}][\mathrm{Ca^{2+}}]^2, \\
\frac{\mathrm{d}[\mathrm{Ca_4CaM}]}{\mathrm{d}t} &= k_{24}[\mathrm{Ca^{2+}}]^2[\mathrm{Ca_2CaM}] - k_{42}[\mathrm{Ca_4CaM}] + k_{CaN4Off}[\mathrm{Ca_4CaMCa_4CaN}] \\
&\quad - k_{CaN4On}[\mathrm{Ca_4CaM}][\mathrm{Ca_4CaN}], \\
\frac{\mathrm{d}[\mathrm{Ca_4CaN}]}{\mathrm{d}t} &= k_{CaNCaOn}[\mathrm{Ca^{2+}}]^2[\mathrm{Ca_2CaN}] - k_{CaNCaOff}[\mathrm{Ca_4CaN}] \\
&\quad + k_{CaN4Off}[\mathrm{Ca_4CaMCa_4CaN}] - k_{CaN4On}[\mathrm{Ca_4CaM}][\mathrm{Ca_4CaN}], \\
\frac{\mathrm{d}[\mathrm{Ca_4CaMCa_4CaN}]}{\mathrm{d}t} &= k_{CaN4On}[\mathrm{Ca_4CaM}][\mathrm{Ca_4CaN}] - k_{CaN4Off}[\mathrm{Ca_4CaMCa_4CaN}].
\end{aligned}
$$

Here, square brackets denote concentrations, and rate parameter values are given in Table S1, using the same notation and parameter values as Saucerman & Bers (2008).

References

Bazzazi, H. et al., (2015). Novel fluorescence resonance energy transfer-based reporter reveals differential calcineurin activation in neonatal and adult cardiomyocytes. *The Journal of Physiology* 593, 3865–84.

Kar, P. and Parekh, A. B. (2015). Distinct spatial Ca2+ signatures selectively activate different NFAT transcription factor isoforms. *Molecular Cell* 58, 232-243.

Saucerman, J.J. & Bers, D.M. (2008). Calmodulin mediates differential sensitivity of CaMKII and calcineurin to local Ca2+ in cardiac myocytes. *Biophysical Journal* 95, 4597–612.

Tomida T, Hirose K, Takizawa A, Shibasaki F, Iino M. (2003). NFAT functions as a working memory of Ca2+ signals in decoding Ca2+ oscillation. *EMBO Journal* 22, 3825–32.
